# Linking the composition of cryoconite prokaryotic communities in the Arctic, Antarctic, and Central Caucasus with their chemical characteristics

**DOI:** 10.1038/s41598-024-64452-3

**Published:** 2024-07-09

**Authors:** Grigory V. Gladkov, Anastasiia K. Kimeklis, Rustam Kh. Tembotov, Mikhail N. Ivanov, Evgeny E. Andronov, Evgeny V. Abakumov

**Affiliations:** 1 Department of Applied Ecology, St. Petersburg State University, Saint-Petersburg, Russia 199034; 2 Laboratory of Microbiological Monitoring and Bioremediation of Soils, All-Russian Research Institute for Agricultural Microbiology, Pushkin, Russia 196608; 3 Tembotov Institute of Ecology of Mountain Territories, Russian Academy of Sciences, Nalchik, Russia 360051; 4 Department of Cryolithology and Glaciology, Lomonosov Moscow State University, GSP-1, Leninskie Gory, Moscow, Russia 119991; 5 V.V. Dokuchaev Soil Science Institute, Moscow, Russia 119017

**Keywords:** Cryoconite, Prokaryotic community, Chemical parameters, Geographic location, Amplicon sequencing, 16S rRNA, Microbial ecology, Environmental chemistry, High-throughput screening, Boreal ecology

## Abstract

Cryoconites are the deposits on the surface of glaciers that create specific ecological niches for the development of microorganism communities. The sediment material can vary in origin, structure, and nutrient content, creating local variations in the growth conditions. An additional factor of variability is the location of the glaciers, as they are found in different climatic zones in the high mountain regions and closer to the poles. Here, using the analysis of amplicon sequencing of the 16S rRNA gene, we studied the taxonomic composition of the prokaryotic communities from glaciers from remote regions, including the Arctic (Mushketova on the Severnaya Zemlya, IGAN in Polar Ural), Antarctic (Pimpirev on the Livingstone Island) and Central Caucasus (Skhelda and Garabashi) and connected it with the variation of the physicochemical characteristics of the substrate: pH, carbon, nitrogen, macro- and microelements. The cryoconite microbiomes were comprised of specific for this environment phyla (mostly Pseudomonadota, Cyanobacteria, Bacteroidota, Acidobacteriota, and Actinobacteriota), but each glacier had a unique taxonomic imprint. The core microbiome between regions was composed of only a few ASVs, among which the most likely globally distributed ones attributed to *Polaromonas* sp., *Rhodoferax* sp., *Cryobacterium* sp., and *Hymenobacter frigidus*. The WGSNA defined clusters of co-occurring ASVs between microbiomes, that significantly change their abundance corresponding with the variation of chemical parameters of cryoconites, but do not fully coincide with their regional separation. Thus, our work demonstrates that the chemical characteristics of the sediment material can explain the variation in the cryoconite prokaryotic community which is not always linked to geographic isolation.

## Introduction

Recently, the concept of glaciers as sterile ecosystems has changed to the perception of glaciers as environments that are inhabited by various organisms^1,2^. Due to climatic conditions and high biological activity, glaciers are considered the coldest biome on the planet^3^. The surface represents the most biologically active part of glaciers, which leads to the proliferation of microbial communities^4–6^. Microbial habitats on the surface of glaciers are mainly cгyoconite holes—water-filled depressions with cryoconite material at the bottom^7,8^. Cryoconite is a widespread, black-colored sediment formed on the glacier surface as a result of interaction between mineral particles present on the ice and complex microbial communities that develop on the glacier surface. Cryoconite consists of mineral and organic components, they contain archaea, fungi, algae, cyano-, and heterotrophic bacteria as the basis^1,9,10^. Cryoconite on the surface of glaciers accumulates in a pseudo cylindrical or bowl-shaped form, forming a lump, which under the influence of melting of the darkly colored mass can deepen downward and expand horizontally. Deepening downward and enlarging, cryoconite contributes to the acceleration of glacier melting^4,9,11^. Cryoconite holes represent a special ecosystem on the glacier surface, where numerous microorganisms can live in an active state.

One of the important properties of cryoconites is polydispersity—they contain mineral and organic particles of various sizes, i.e., ecological micro-niches^12^. In this context, considering cryoconite holes as geochemical predictors of periglacial soil formation and as soil-like bodies is a promising approach in soil science and soil geography^13^. It was shown that cryoconites of glaciers and ice-marginal environments are quite different in terms of microbiome composition^14^. The ecological niches of cryoconites are the first to encounter the effects of external impact on glaciers. Later, when glaciers melt, components of the microbiome can enter geochemically subordinate landscapes, affecting glacial soils, as well as the microbiome of mountain rivers. In this regard, the study of cryoconite ecosystems, including their microbial diversity, is the most important issue of modern ecological and microbiological science. Thus, the creation of a database of the cryoconites microbiota is a frontier of the current glacial microbiology^15^.

The studies of the microbiome of cryoconites from glaciers of polar regions have recently received increasing attention. The application of high-throughput sequencing techniques revealed that the most common taxa in cryoconite microbiomes of various regions, including the East Antarctic Anuchin Glacier (Untersee oasis)^16^, the Taylor Valley^17^ are Pseudomonadota, Cyanobacteria, Bacteroidota, Actinobacteriota, and Acidobacteriota. The study of the anaerobic component of a cryoconite community from the Ecology Glacier on King George Island revealed a lack of Cyanobacteria and a presence of Planctomycetota^18^. During the extensive investigation of several locations across the Arctic and Antarctic, it was shown that while the same biotic niches were occupied in both regions, each maintained a distinct core microbial community^19^. Yet, it was also shown that microniches play an important role in the formation of the biodiversity of cryoconites on the local scale^20^. The study in Greenland^21^ showed that the bacterial community varied depending on the size of cryoconites particles, due to different nutrient availability: it became more similar as the size of cryoconite fractions increased, while the smallest fractions contained more unique genera in each glacier. Thereby, high sequence similarities between samples within one region indicate the possibility of long-range atmospheric transport mechanisms that complement local inoculation sources^16^, proposing the idea that the microbiome of cryoconites is affected by both external and internal (local) factors, as it was shown on the cryoconites of Asian glaciers^22^. Thus, the study of the cryoconite microbiome is currently very active in the polar regions of the Earth. But, in addition to polar regions, it is also important to study the microbiome in cryoconites of high-mountain glaciers, which, along with polar regions, are among the most vulnerable regions due to unprecedented climate change.

Several works were devoted to the microbiome of cryoconite communities on the high-altitude glaciers of Asia—the significant reserves of the Earth's ice masses. It has been shown that the cryoconite microbiome on the high-altitude Asian glacier demonstrates taxonomic and metabolic distinction from polar regions, connected with regional or glacier-specific physicochemical conditions^22^. Similar study was carried out in the Tien Shan Mountains, which are the largest mountain systems in Central Asia with glaciers over 3500 m above sea level^4,23^. They showed the metabolic importance of cyanobacteria in cryoconite microbiomes and their global distribution at the OTU level but also highlighted that most phylotypes are fundamentally endemic to certain areas at the population level, indicating limited migration between regions. A study of the bacterial community of cryoconites from glaciers conducted on the Tibetan Plateau showed the importance of rare taxa in the community assembly process after a snowfall disturbance^24^.

The microbiome of cryoconite from glaciers of the European Alps has been studied in sufficient detail^7,11,25^. It was shown that cryoconite from the Stubaier Glacier (Alps, Austria) is a habitat for a significant number of heterotrophic microorganisms capable of growing and surviving in extreme environmental conditions^26^. Interesting results were published about the relationship between the microbiome of cryoconites and the microbiome of tardigrades; the presence of these invertebrates can affect the structure of the microbiome of the “cryoconite ecosystem”^27^. A strong anthropogenic impact on the alpine glacier cryoconite microbial communities was shown^28^.

Some of the works cover the Andes^29^, the results of which showed that bacterial communities in cryoconites of South American glaciers were similar to those observed on other continents but they have fewer percentages of Cyanobacteria. They also revealed the linkage of the cryoconite bacterial community composition differences with geographical isolation and chemical characteristics of the substrate.

The Caucasus Mountain range is one of the largest in Europe, stretching for over 1200 km with Mount Elbrus (5642 m) being its highest peak. Despite this, very little attention has been paid to the study of the microbiome of cryoconite from the glaciers of the Caucasus, probably due to its borderline position with Asia. Thus, the scientific literature contains a limited number of works devoted to the studies of Caucasian cryoconites^30,31^. These works were carried out in Transcaucasia, on glaciers in Georgia. As for the Central Caucasus, one paper was published for this territory^20^, in which a metaproteomic assessment of cryoconite in this territory was carried out. In this work, cryoconite communities from the less polluted glaciers of the Central Caucasus were compared with the heavily polluted glaciers of Novaya Zemlya. It was revealed that cyanobacteria proteins prevailed in both sites, but the authors found a slight shift towards heterotrophic bacteria in the Caucasus. A short report^32^, devoted to the structure and diversity of the microbiome of cryoconite from glaciers of the Central Caucasus, was also published. The scope of this research is extremely small for such a large and important territory as the Caucasus. As for the glaciers of the Polar Urals, there are no studies of the cryoconite microbiome for this area. Even more scarce are the studies connecting the microbial diversity of cryoconites with the chemical composition of the substrate on the global and local scale. An earlier study used pH and O_2_ concentrations of cryoconite holes for comparing the composition of their microbial communities across five glaciers, all from the same mountain range^29^.

Based on the above, this work aimed to unite in one study the analysis of the cryoconite microbiome of glaciers located in different, including poorly covered in literature, geographical locations and to link their composition with the primary physicochemical parameters of the habitat, including pH, carbon, nitrogen, macro, and microelements content. The following objectives have been settled: (1) to analyze the physicochemical properties of the cryoconite materials, (2) to analyze the taxonomy composition of the microbiome of cryoconites collected from various glaciers across the globe using 16S rRNA gene amplicon sequencing, and (3) to connect the variation in the prokaryotic composition of cryoconites with physicochemical and geographical factors.

## Materials and methods

### Sampling of the cryoconite material

The study covers cryoconites from five glaciers from three parts of the globe—the Arctic, Antarctic, and the Caucasus (Table 1, Figure S1). The Arctic destination includes glaciers IGAN (Polar Ural Mountains) and Mushketova (Severnaya Zemlya archipelago). The Antarctic is presented by the Pimpirev Glacier from Livingstone Island. The Caucasus includes the Garabashi and Skhelda glaciers. These locations were accessed via separate expeditions in 2020–2021. The distances between glaciers range from 20 km (Garabashi and Skhelda glaciers) up to 18,000 km (Pimpirev and Mushketova) (Figure S1). Glaciers, as well as sampling plots, varied in height above sea level, ranging from 800 m for Pimpirev, to 2000–3000 m on the Caucasus. On each glacier, several plots (2–5) were selected for sampling, depending on the accessibility of the material. In total, 17 sampling plots have been included in the analysis of the microbiome composition of cryoconite material (Table S1). For this research, samples from different plots from one glacier were considered biological replicates. As an exception, the samples from Pimpirev Glacier were divided into two isolated sites (PAG/PAGOr), because two of the four plots carried cryoconite material of ornithogenic origin, which related to the fact that skua colonies were located on the nunatak directly above this part of the glacier. Additional information about the plots' characteristics was described previously^33–35^. Thus, when processing the data, all pots were divided into six sites isolated from each other. Cryoconite material in all locations was collected at the bottom of well-like depressions on the ice surface and then stored at − 20 °C until transportation to the laboratory for the subsequent analysis. All the chemical analyses were conducted from grounded fine material, while microbiological research was conducted from the bulk frozen samples.

**Table 1:** List of glaciers with the general characteristics of cryoconite sampling sites.

Sampling site/code	General characteristics of the site	Coordinates	Elevation, MAMSL	Date of expedition
Pimpirev glacier, mineral material/PAG	Western Antarctica, South Shetland archipelago, Livingstone Island, receding glacier	62.41.08 S, 60.24.20 W	109	January 2020
Pimpirev glacier, mineral material with ornithogenic effect/PAOr	Western Antarctica, South Shetland archipelago, Livingstone Island, receding glacier	62.41.23 S, 60.25.04 W	88	January 2020
IGAN Glacier/IGAN	Polar Ural cow-valley glacier	67.34.32 N, 66.02.00 E	1100	August 2021
Mushketova glacier/Mush	Severnaya Zemlya, mountain-valley glacier	79.05.46 N, 101.51.25 E	420	August 2021
Garabashi glacier/Garabashi	Central Caucasus, receding glacier	43.18.18 N, 42.27.50 E	3400	September 2021
Skhelda glacier (Baksan gorge)/Skhelda	Central Caucasus, alpine valley glacier	43.11.27 N, 42.38.45 E	2422	September 2021

*MAMSL* metres above mean sea level. IGAN is abbreviated after the name of the Institute of Geography in Moscow, Russia.

### Chemical analyses of the cryoconite material

Prior to the chemical analyses, cryoconite material was grounded and air-dried. The pH of the cryoconite water extract was measured by the pH meter Milwaukee Mi106 (Milwaukee Electronics (USA). The total carbon (TOC) and nitrogen (TON) contents were determined by the method of dry combustion with the use of an elementary analyzer LECO TruSpec MICRO (LECO Corporation, St Joseph, MI, USA). Quantitative determination of mobile forms of nitrogen (N–NH_4_ and N–NO_3_) was carried out using potassium chloride solution according to the standard^36^. Determination of mobile forms of potassium (K_2_O) and phosphorus (P_2_O_5_) was carried out by the Kirsanov method, based on extraction of mobile forms of potassium and phosphorus from samples using 0.2 M HCl solution followed by quantitative determination of phosphorus content on photoelectric colorimeter and potassium content on flame photometer^37^. Concentrations of trace elements (Cu, Pb, Ni, Cd, and Zn) were determined by the atomic absorption spectroscopy (AAS) and atomic emission spectroscopy (AES) methods on the Spectrophotometer Kvant 2M (Moscow, Russia). The extraction of elements was carried out using 1 M HNO_3_ and acetate-ammonium buffer solution with pH 4.8^38^. The contents of primary macro- and microelements were determined for each sample in one to four replicates, depending on the availability of the substrate. More detailed information on determining chemical characteristics was described earlier^35^. Significant differences in chemical parameters between sites were accessed by ANOVA with Tukey’s HSD test^39^. The z-scored values (relative to the means) of the results were clustered using the UPGMA algorithm in the R software environment v. 4.2^40^.

### Analysis of taxonomic composition of cryoconite material

From each cryoconite sample DNA was extracted in four replicates (68 in total) from 0.1 g of the material using the NucleoSpin Soil Kit (Macherei Nagel GmbH & Co. KG, Germany) which was used for the amplification of the V4 region of the 16S rRNA gene with primers adapted for Illumina sequencing based on 515f. (GTGCCAGCMGCCGCGGTAA) and 806r (GGACTACVSGGGTATCTAAT)^41^. PCR was performed in 15 μl of a reaction mix containing 0.5 U of Q5® High-Fidelity DNA Polymerase (New England BioLabs, USA), 1X Q5 Reaction Buffer, 5 pM of each primer, 3.5 mM dNTP (Evrogen, Russia), and 1–10 ng of DNA template on a T100 Thermal Cycler (BIO-RAD Laboratories, USA) with the following protocol: 94 °C for 1 min; 25 cycles of 94 °C for 30 s, 55 °C for 30 s, 72 °C for 1 min; and 72 °C for 3 min. The product was cleaned and prepared for pair-end sequencing on the Illumina Miseq platform (Illumina, Inc., San Diego, CA, USA) according to the manufacturer’s recommendations.

The data from the sequenced 16S rRNA gene amplicon libraries were processed into an amplicon sequence variant (ASV) table, using the DADA2 pipeline^42^ in the R software environment v. 4.2^40^ as described earlier^43,44^. Taxonomy was assigned to the output sequences using the Silva 138.1 database^45^. The alpha-diversity (within the microbiomes) of the rarified data was accessed by the following indices: observed (number of ASVs, indicates richness), Shannon^46^ (indicates both richness and evenness), and inverted Simpson^47^ (indicates evenness). Significant differences in the indices were accessed by ANOVA with Tukey’s HSD test. The beta-diversity (between microbiomes) for the CLR-transformed^48^ data was visualized with NMDS^49^ with the Bray–Curtis distance matrix^50^. The Canonical Correspondence Analysis (CCA) in vegan^51^ was performed to connect the microbiome composition of the cryoconite material with the variability of its chemical characteristics. PERMANOVA was used to evaluate the effects of the region, sampling site, and chemical parameters on the microbiome composition^52^. The Weighted Correlation Network Analysis (WGCNA)^53^ was used for the clusterization of co-occurrent ASVs for the cryoconite microbiomes in the whole dataset. Before analysis, the ASV table was filtered from rare reads, so only those that had at least 10 reads in at least more than 10% of the libraries were left. The Analysis of Compositions of Microbiomes with Bias Correction (ANCOM-BC)^54^ was used for the search for structural zeros^55^ in the core microbiome, the normalization of data before WGCNA, and the differential abundance test for all the samples on the phylum level and for samples from the Pimpirev Glacier on the ASV level. The analyses were carried out using the phyloseq^56^ and ampvis2^57^ packages. The code is available in the Supplement.

## Results

### Chemical characteristics of the cryoconites

The relative data on the chemical characteristics of studied cryoconite deposits were visualized via UPGMA clustering; the absolute values are available in the supplement (Table S1). The pH values between geographic locations varied essentially: the lowest pH was revealed for Antarctic (PAG, PAOr) and Arctic (IGAN, Mush) cryoconites, while cryoconites from the Central Caucasus (Skhelda, Garabashi) were less acidic. Total organic carbon (TOC) and nitrogen (TON) contents were low across all samples, except for ornithogenically affected cryoconites in the Antarctic. The cryoconites from Antarctic and Garabashi had increased ammonium nitrogen (N-NH_4_) and potassium (K_2_O) quantities. A characteristic feature of all cryoconite samples was the almost complete absence of nitrate nitrogen (N-NO_3_), with the highest values detected in Mushketova. In terms of macroelements, the cryoconites of Central Caucasus were more enriched by phosphorus (P_2_O_5_). The content of trace elements in the studied materials was quite variable, most of which were found in the Arctic. The highest values of Zn, Pb, Cu, and Cd were detected in the IGAN, and Ni—in the Mushketova.

**Figure 1:**
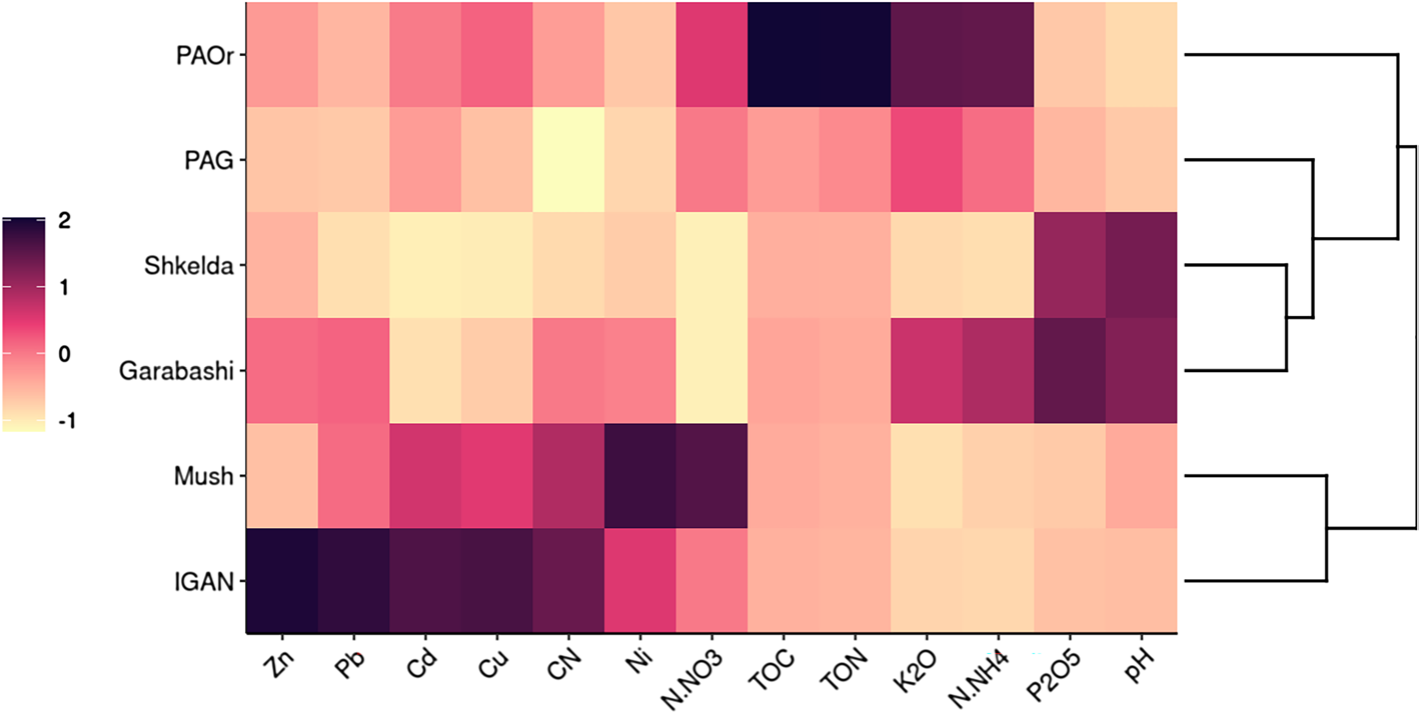
Heatmap of z-scored chemical characteristics of the cryoconite material. Average values are in orange (medium color), values above average are violet (darker), and below average—yellow (lighter). The clusterization of samples based on these parameters was performed by the UPGMA. Sample location: PAG/PAOR - Antarctic, Shkelda/Garabashi - Central Caucasus, Mush/IGAN - Arctic.

The UPGMA clustered the cryoconite samples by their chemical parameters into groups with defining features, almost corresponding with their geographic regions: (1) Pimpirev ornithogenic (PAOr) samples with high values of TOC and N-NH_4_; (2) Skhelda and Garabashi samples with acidic pH and high P_2_O_5_ content; (3) IGAN and Mushketova samples with relatively high concentrations of N-NO_3_ and microelements (Fig. 1). PAG samples had intermediate chemical values and were clustered between Antarctic and Caucasus samples.

### Alpha and beta-diversity of the prokaryotic community of cryoconite sediments

The 16S rRNA gene libraries sequencing retrieved 1,284,969 reads, classified into 5077 ASVs. More than 81% of ASVs and 94% of the reads were unique for each of the sampling regions (Arctic, Antarctic, Caucasus) (Figure S2).

**Figure 2:**
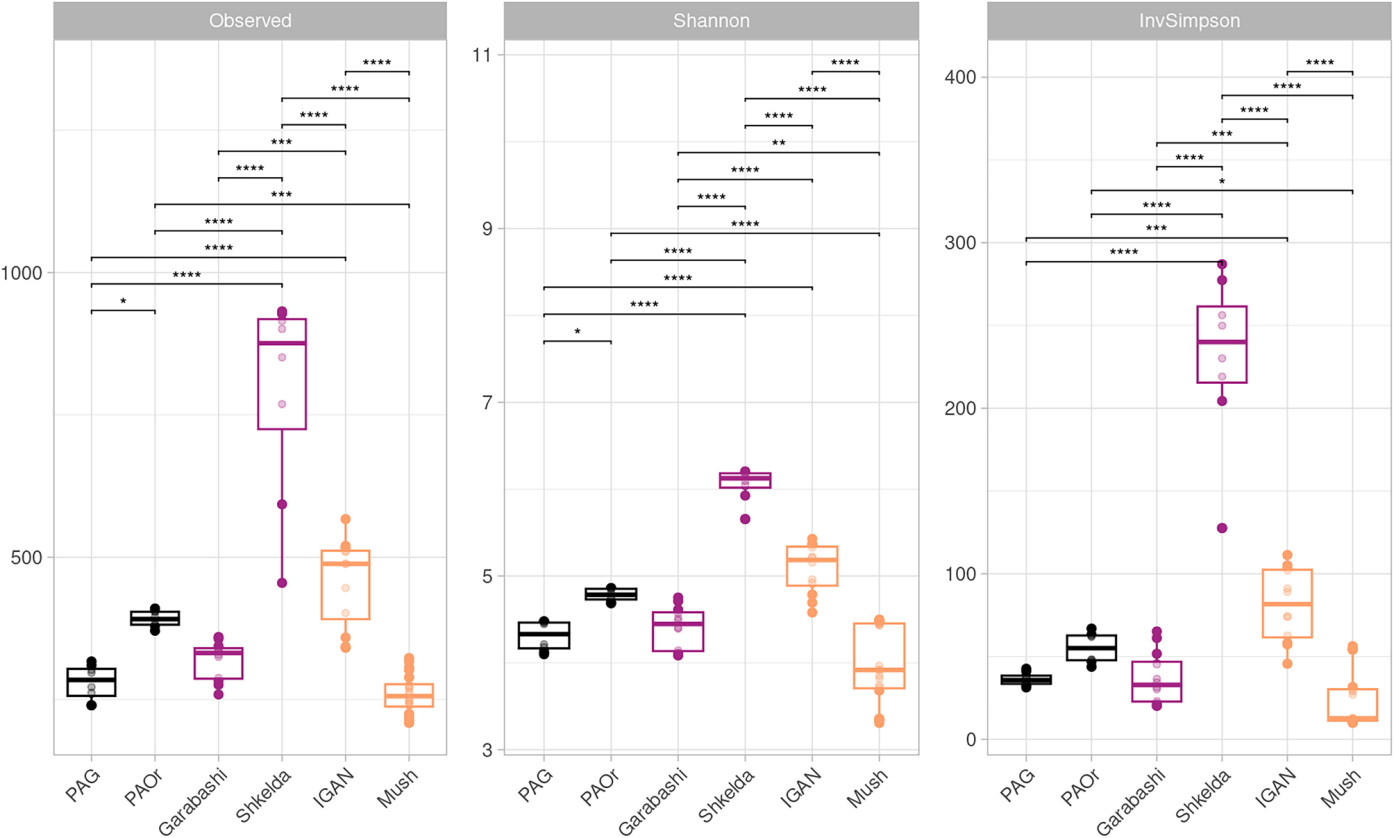
Alpha diversity indices (Observed, Shannon, InvSimpson) of prokaryotic communities of cryoconites, based on the data from 16S rRNA gene amplicon libraries. Significant differences were assessed by ANOVA with Tukey HSD test: (*) *p* value < 0.05; (**) *p* value < 0.01; (***) *p* value < 0.001; (****) *p* value < 0.0001. Colors represent the location of sampling: Antarctic (PAG, PAOr)—black, Caucasus (Skhelda, Garabashi, Mud, Soil)—violet, Arctic (IGAN, Mushketova)—yellow.

The rarified indices of alpha diversity demonstrated significant differences in the richness (observed) and evenness (Inv. Simpson, Shannon) of the deposit microbiomes from different sites both between and within geographical regions (Fig. 2). The richness values varied between 289 and 844, with the lower ASV count detected in PAG, Garabashi, and Mushketova, slightly higher numbers in PAOr and IGAN cryoconites, and the highest in Skhelda. The same trends were observed for the Inv. Simpson and Shannon indices. Thus, all sites in the dataset had different values of microbial diversity, which could not be directly linked with the geographical location (Arctic, Antarctic, Central Caucasus).

**Figure 3:**
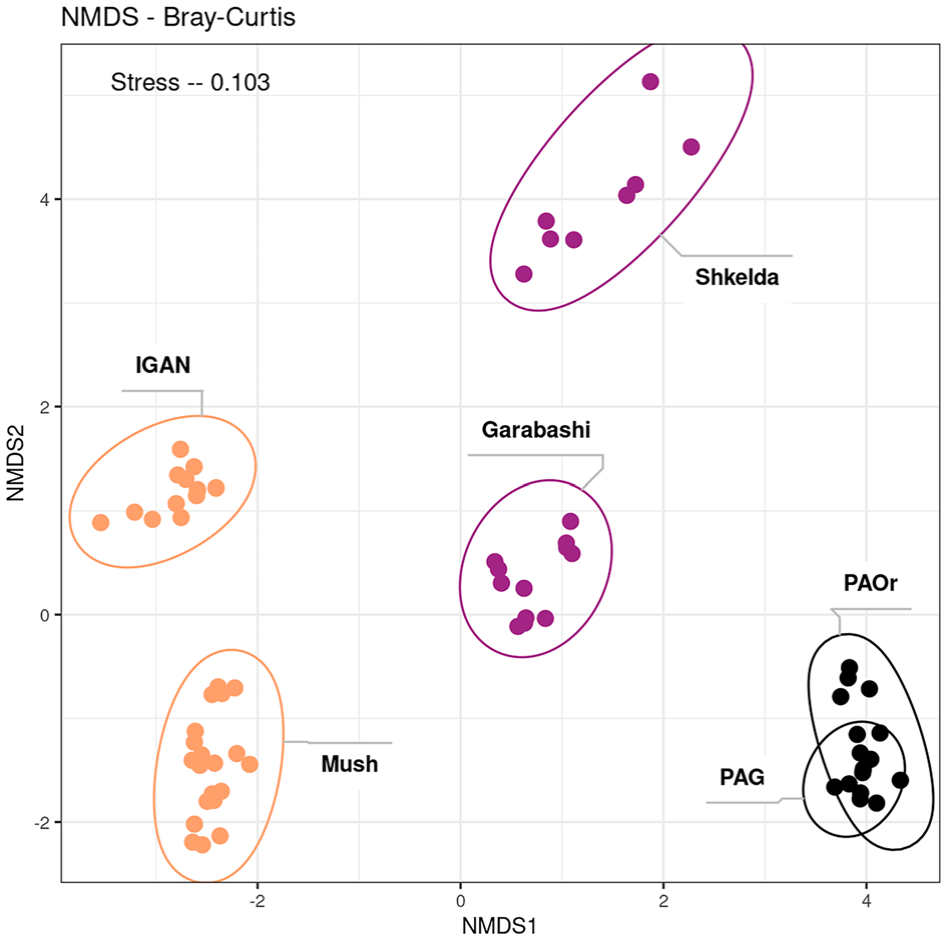
NMDS plot of the beta-diversity of the prokaryotic community accessed with Bray–Curtis, based on the 16S rRNA gene amplicon sequencing data. Samples from one site are surrounded by ellipses. Colors represent the location of sampling: Antarctic (PAG, PAOr)—black, Caucasus (Skhelda, Garabashi, Mud, Soil)—violet, Arctic (IGAN, Mushketova)—yellow.

On the NMDS plot of the beta-diversity plot, the placement of cryoconite microbiomes from different sites reflected their mutual geographical position (Fig. 3). On the left side of the plot, there are cryoconite samples from Arctic locations (IGAN, Mushketova), on the right side—samples from the Antarctic (PAG, PAOr), and in the middle are two sampling sites from Central Caucasus (Garabashi, Skhelda). Despite the remoteness of sampling regions, according to PERMANOVA, the region was a less significant predictor (R^2^ = 0.52, *p* value < 0.001) of the distances between microbiomes than the sampling site (R^2^ = 0.79, *p* value < 0.001) (Table S3). So, cryoconite microbiomes from different glaciers retain significant differences even though they were isolated from the nearby regions. In the case of Skhelda and Garabashi, this distance could correspond with the differences in the richness of these samples. Though mineral and ornithogenic samples of cryoconites from Pimpirev Glacier (PAG/PAOr) have different chemical properties and alpha indices values, they don't diverge from each other on this scale and demonstrate a low value of R-squared (R^2^ = 0.25, *p* value < 0.002).

### Taxonomic composition of the major bacterial component and the core ASVs

**Figure 4:**
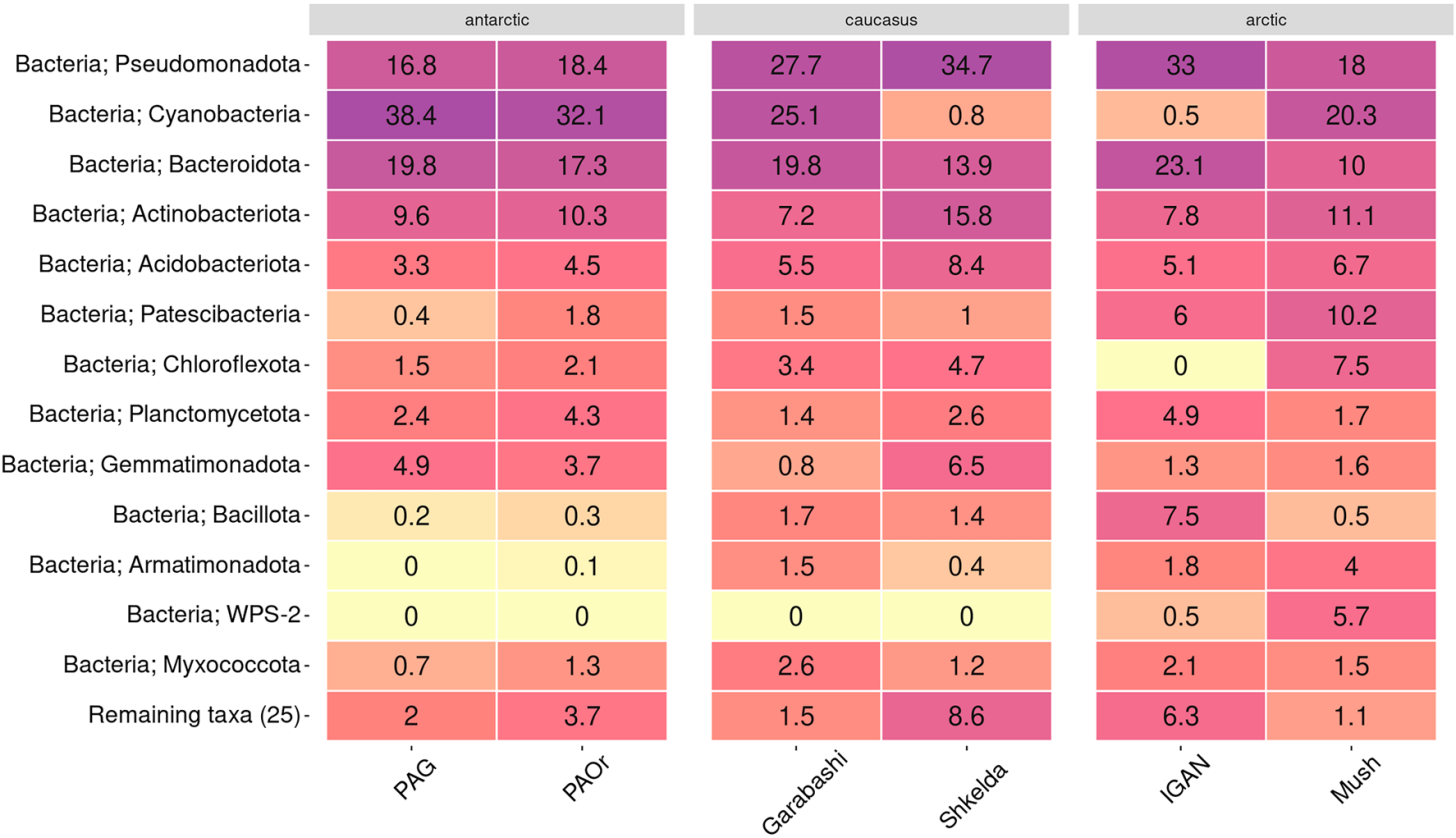
The heatmap of the 13 most abundant phyla in the prokaryotic community of cryoconites, based on the 16S rRNA gene amplicon sequencing data. Numbers show relative abundance in % of total read count in the sampling set. A darker color shows higher values, and a lighter—lower. Columns are grouped according to the region of sampling.

Most of the ASVs of the dataset were attributed to bacteria, while archaea were a minor component of cryoconites microbiomes (Fig. 4, Figure S3). The most prevalent phyla in the cryoconites were Cyanobacteria, Pseudomonadota, Bacteroidota, Actinobacteriota, Acidobacteriota, Patescibacteria, and Chloroflexota (Fig. 4). The composition of all phyla, except Actinobacteriota, Acidobacteriota, and Myxococcota, in the microbiomes of cryoconites differed significantly between each other depending on the sampling region and didn’t follow a clear geographical pattern (Figure S4). Cyanobacteria were dominant in PAG, PAOr, Garabashi, and Mushketova and almost absent in Skhelda and IGAN. Instead, the latter two locations were abundant in Pseudomonadota. The IGAN was lacking Chloroflexota but had a higher representation of Bacillota and Patescibacteria. In comparison with other sites, PAG, PAOr, and Mushketova had a lower representation of Bacillota.

On the lower taxonomic resolution, the ASVs from the cryoconite prokaryotic communities were attributed to various genera across the regions, the most numerous being *Phormidesmis*, *Ferruginibacter*, *Chamaesiphon*, *Tychonema*, *Polaromonas*, *Hymenobacter*, *Sphingomonas*, and *Gemmatimonas*. The relative abundance of these taxa shifted between regions, additionally, some other genera were present only in some locations, for example, *Granulicella* in the Arctic (Figure S5).

Because of the uniqueness of cryoconite as a habitat, we decided to seek out the most likely universally distributed ASVs across all regions. For this, we did the search and the exclusion of the structural zeros in the dataset aimed at reducing the effect of possible cross-contamination between samples during the sequencing process. The core microbiome of all cryoconite samples between regions before filtering consisted of only 254 ASVs, which accounted for less than 1% of the total read number (Figure S2). After the removal of structural zeros, only 62 ASVs were left (Figure S6), four of which, attributed to *Polaromonas* sp., *Cryobacterium* sp., *Rhodoferax* sp., and *Hymenobacter frigidus*, were found to be ubiquitous in all regions. So, even if the same genera were detected in cryoconites from distant geographic locations, they were mostly presented by ASVs, unique for each region.

To conclude, the following patterns about cryoconite microbiomes can be stated: (1) cryoconite ecological niche facilitates the growth of specific microbiota; (2) each geographical location is characterized by its unique set of genera; (3) concurrent genera in distant locations are presented by different ASVs; (4) the global core of the cryoconite microbiomes is minuscule and consists of a few ASVs.

### The influence of geographical location and chemical composition of cryoconites on their prokaryotic community composition

The variations in the composition of ASVs could be explained by differences in the conditions of the environment. To assess these variations of cryoconite microbiome composition between locations we tried to link them with their chemical characteristics using the CCA and WGCNA. The analysis included all parameters, except TON and K_2_O because they were collinear with other data

**Figure 5:**
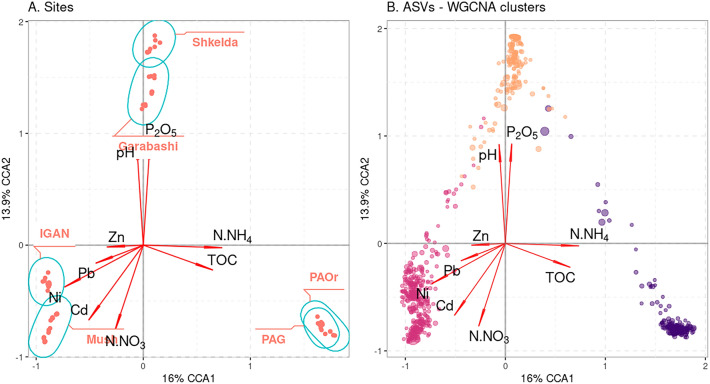
The CCA plots, which show the correspondence of chemical parameters of cryoconites (red arrows) with: (**A**) cryoconite prokaryotic communities from different sites (surrounded by ellipses), (**B**) individual ASVs, clustered by WGCNA. TOC—total organic carbon. Violet group—ASVs prevalent in Antarctic locations (IGAN, Mushketova) with high organic carbon and ammonium content, pink—Arctic (Pimpirev) with high content of microelements and nitrates, orange—Caucasus (Skhelda, Garabashi) with alkaline pH and high phosphorus content.

The CCA plot demonstrates a strong correspondence of cryoconite microbiomes both with geographical location and chemical characteristics (Fig. 5A). For the microbiomes of the Antarctic samples (PAG, PAOr), the influence of TOC and N-NH_4_ was shown; for the Caucasus samples (Skhelda and Garabashi), the main microbiome composition factors were pH and P_2_O_5_; for the microbiome from the Arctic samples (IGAN and Mushketova), the positive impact of trace elements (Ni, Cu, Cd) was more pronounced. This grouping coincides with the UPGMA clustering of cryoconite chemical characteristics performed above. However, according to PERMANOVA, the sum of all chemical parameters used in this dataset was a stronger predictor of microbiome composition than their isolation site (R^2^ = 0.84, *p* value < 0.001).

The CCA plot with the 50 most abundant ASVs from the dataset has shown how their abundance corresponds with chemical factors (Figure S7). For Pimpirev these were ASVs from Cyanobacteria (*Phormidium*, *Chamaesiphon*), Bacteroidota (*Cytophagales*, *Hymenobacter*), *Gemmatimonas*, for Caucasus samples—Cyanobacteria (*Tychonema*, *Chamaesiphon*), Pseudomonadota (*Polaromonas*, *Rhizobacter*, *Sphingomonas*), Bacteroidota (*Ferruginibacter*), for Arctic—various representatives of Bacteroidota, Cyanobacteria, Chloroflexota, Pseudomonadota.

The WGCNA was applied to define clusters of co-occurring ASVs in the dataset and test their response to regional and chemical factors. After filtering the data, around 700 ASVs were left and divided into 9 clusters with a strong (R > 0.5) and significant (*p* value < 0.05) correlation with chemical parameters (Figure S8). They were further combined into three groups, as presented on the CCA plot with ASVs: pink—connected to the high content of microelements and nitrates, orange—to alkaline pH and high phosphorus content, violet—to high TOC and ammonium content (Fig. 5B). Comparison of data on chemical characteristics, CCA, and WGCNA plots suggests that these groups of ASVs correlate with the locations of sampling as well as differences in their chemical characteristics, but not entirely (Table S2). For example, there were ASVs, which correspond with NH_4_ content and were detected both in Antarctic and Caucasus locations. There were also ASVs, present in both Arctic and Caucasus regions, though none of the calculated metrics explain this dispersion. Remarkably, from 700 ASVs used in the cluster analysis, none were abundant both in the Arctic and Antarctic regions.

**Figure 6:**
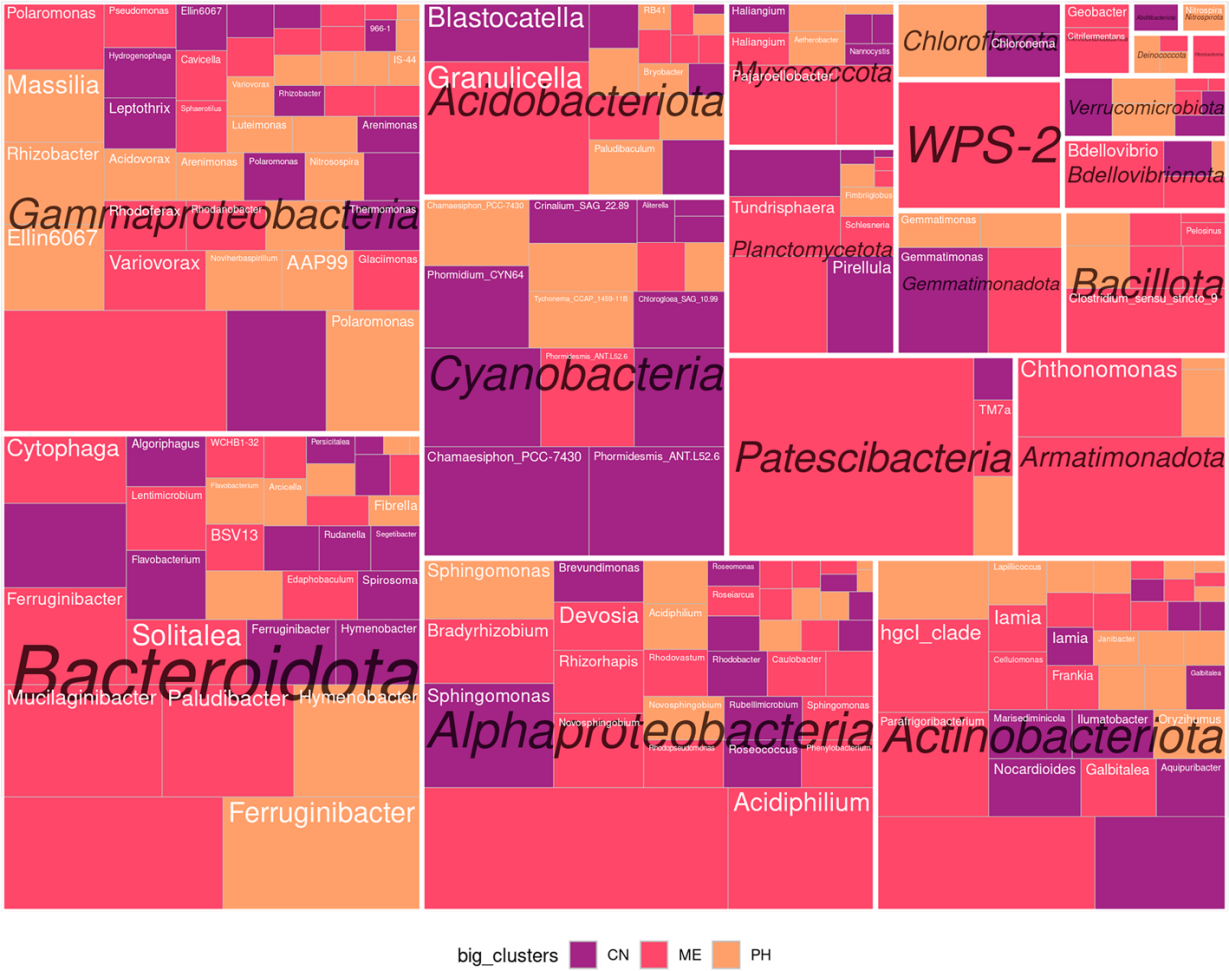
The treemap chart, which shows the abundance of taxa in each of the three clusters of co-occuring ASVs, defined by WGCNA. Violet group—ASVs correlating with TOC and N-NH_4_, pink—with high content of microelements, orange—with alkaline pH and P_2_O content. Blocks represent ASVs, combined by genus and contain the name of the lowest defined taxonomic rank. Blank blocks represent ASVs, not defined on the genus level.

The treemap chart of the taxonomic composition of clusters of co-occurring ASVs, defined by the WGCNA, showed that most of the major phyla were common for all three groups (Fig. 6). According to the above results on the genus composition between samples (Figure S5), the differences between the clusters were often observed at the level of ASVs within the same genus—for example, different representatives of *Phormidesmis*, *Ferruginibacter*, *Polaromonas*, and others were abundant in all clusters (Table S2). Along with this, each cluster had a specific taxonomic response to the chemical parameters of cryoconite material. ASVs, related to TOC and N-NH_4_, were more abundant in Cyanobacteria (*Phormidium*, *Crinalium*, *Chlorogloea*), and were lacking Armatimonadota, Patescibacteria, and Bacillota. ASVs, which reacted to microelements and nitrates, included Acidobacteriota (*Granulicella*, *Solibacter*), Actinobacteriota (*Parafrigoribacterium*, Sporichthyaceae), Acetobacteraceae, Bacillota, Bacteroidota (*Paludibacter*, *Mucilaginibacter*, *Solitalea*). ASVs, reacting to pH and phosphorus, didn't attribute to the unique taxa but were more prevalent in Cyanobacteria (*Tychonema*, *Phormidesmis*), Gammaproteobacteria (*Polaromonas*, *Rhodoferax*, *Rhizobacter*), Bacteroidota (*Ferruginibacter*). So, we can assume that while Arctic and Antarctic cryoconites harbored ASVs of unique taxonomic fingerprints, those from Caucasus belonged to more globally distributed taxa.

To assess the influence of the ornithogenic origin of cryoconite substrate on the microbiome composition we applied a differential abundance test from ANCOM-BC (Figure S9) on samples from Pimpirev Glacier. Initially, more than 96% of reads were common between PAG and PAOr, but the latter had higher richness (Figure S10). Both sites were characterized by a high abundance of Cyanobacteria (*Phormidium*, *Crinalium*, and *Chamaesiphon*). PAOr was generally more abundant in ASVs from most phyla, including Gammaproteobacteria, Alphaproteobacteria, Planctomycetota (*Pirellula*, *Gemmata*, *Fimbriiglobus*), Patescibacteria, and Acidobacteriota, while PAG was more characteristic of several representatives of Bacteroidota, Cyanobacteria, Alpha- and Gammaproteobacteria.

## Discussion

The focus of our research was to study and compare the composition of cryoconite prokaryotic communities from remote regions across the globe, and to connect their variation with the chemical parameters of the sediment material. The studies on cryoconite microbiomes are limited by their inaccessibility and are mostly focused on the Arctic and Antarctic regions, and several mountain ridges, including Alps^7,8,11^ and Andes^29^, while information on other regions is scarce^58^. Here, along with these locations, sampling sites introduced glaciers from the poorly covered areas of Northern Ural and Central Caucasus Mountains.

### Variation of physicochemical parameters

According to the chemical characteristics of the sediment material, each of the six sites had some defining features, but they tended to cluster by regional factor—Arctic, Antarctic, and Caucasus. The differences in pH between Caucasus and other samples could have been caused by the nature of the local mineral substrates that served as sources of cryoconite material. This could also be connected to the available nutrient content, leading to the cryoconites of Central Caucasus being more enriched by P_2_O_5_, and cryoconites from Antarctica having increased K_2_O quantities. A high portion of N-NH_4_ in the cryoconites from the Pimpirev Glacier could be related to the ornithogenic effect, while the accumulation of ammonium could be connected with the acid reaction of these samples. The low concentration of nitrates in cryoconites could be the result of them quickly leaching out in conditions of excess humidity. The various levels of concentration of trace elements in the studied materials could be the origin of mineral materials and accumulation of atmospheric deposits, especially in the case of the glaciers of Central Caucasus. Thus, the chemistry of all studied samples was quite variable and potentially could be an important predictor of the microbiome composition, as it was earlier shown for the soil microorganisms^59^.

### The role of members of the prokaryotic community in the cryoconite microbiomes

Previously, it was shown that one can speak of global similarities in the trophic groups of microorganisms in the cryoconite microbiomes within the Northern and Southern hemispheres^19^. Our data confirms this statement with the addition of cryoconites from Central Caucasus glaciers. The microbiomes of cryoconites from all regions demonstrated common trend in composition, distinct from other environments: they were characterized by a high proportion of a phototrophic component (Cyanobacteria, Chloroflexota)^2,60^, copiotrophs or opportunists (representatives from Pseudomonadota, Bacteroidota, Actinobacteriota)^61^, and specific phylotypes characteristic of psychrotrophic communities (*Polaromonas*, *Parafrigoribacterium*). A high representation of rare phyla, like Patescibacteria^62^, and an almost total absence of Archaea were also shown. The lack of archaea representation can be caused by the bias of the amplification specificity using 16S rRNA gene universal primers towards bacteria^63,64^.

Our results highlight the distinctive role of photosynthetic bacteria from Cyanobacteria and Chloroflexota phyla in the observed ecological niches, with cyanobacteria being often the most abundant group of microorganisms. According to the earlier findings, bacteria from both phyla are the primary producers of organic material in the cryoconite community^65^. A high proportion of bacteria from the copiotrophic-associated phyla is consistent with the previous studies and indicates the high ability of the cryoconite microbiome to adapt to rapidly changing environmental conditions^14,66^. The rare uncultured representatives from the superphylum Patescibacteria were earlier reported to be connected with the alpine permafrost environment^67^.

### Correlation of cryoconite microbiome composition with regional and chemical factors.

Earlier studies assumed that the chemical composition of the substrate is the key to understanding the variation of microbiomes of cryoconites between regions^19^. However, we demonstrated that in different regions, the studied chemical parameters had different impacts on microbiome composition. In Antarctic mineral and ornithogenic sites had contrasting contents of TOC, ammonium, and potassium, but their microbiomes on the scale of the whole dataset were the closest to each other. At the same time, cryoconites from Skhelda and Garabashi had very similar values of pH, phosphorus, and TOC, but their microbiomes were significantly dissimilar. Most probably, different chemical characteristics had unequal effects on the microbiome in various environments.

Another observation is that the most remote regions (Arctic and Antarctic) had the most contrasting chemical parameter values and their microbiomes contained the least amount of common ASVs. At the same time, microbiomes between Arctic and Caucasus, Antarctic and Caucasus bore more common ASVs. In the case of the Antarctic and Caucasus, these similarities could be linked to comparable quantities of ammonium and potassium between these regions, while for the Arctic and Caucasus none of our measurements could explain them.

Despite the common trends in the composition of the prokaryotic community of cryoconites on the phylum level, at the lower taxonomic resolution, they demonstrated differences within one phylum connected to the geographical isolation and variation of chemical parameters. For example, despite the high representation of cyanobacteria overall, we observed that different sampling regions are characteristic of a specific set of taxa from Cyanobacteria. These data are consistent with studies on the biogeography of cyanobacteria, which show uneven distribution of different genera across the globe^4,68^. Studies of Arctic and Alpine cryoconites using TRFLP and pyrosequencing techniques have also shown that they are composed of similar taxa from Pseudomonadota and Cyanobacteria, but these habitats retained specific community structures and metabolomes^25^. It is worth noting that most of the ASVs that differed between the sites belonged to the phylum Bacteroidota. The variability of ASVs within the phylum Bacteroidota observed in this study may be associated with the necessity to utilize various resources of organic carbon, determined by the material of the cryoconite^69^.

The richness of the studied cryoconites reached over 800 ASVs, which is relatively high, compared to cryoconite microbial communities from other studies^7^. The Caucasus mountains region demonstrated unusually high dispersion of richness between local glaciers, with Skhelda glacier having the highest number of ASVs among all samples. One of the reasons could be that Garabashi, along with other glaciers from this study, is an open-type glacier, while Skhelda is debris-covered^35^, which may have led to the increased level of diversity due to the uptake of the microbiome from incoming deposit materials. Another explanation of this phenomenon could be that heterotrophic communities of cryoconites tend to be more diverse than autotrophic^9^. It corresponds with the fact that the prokaryotic community of Skhelda was largely devoid of Cyanobacteria, which were the main autotrophic component in the communities of other glaciers.

As a model for comparing cryoconites from the same region, but with different local factors, cryoconites of ornithogenic and non-ornithogenic origin from Pimpirev Island were considered. On the dataset scale, both groups of Pimpirev samples had overlapping major ASVs, including *Phormidesmis*, *Crinalium*, *Chamaesiphon*, *Gemmatimonas*, *Aquipuribacter*, and *Chlorogloea*. These genera have been previously identified as psychrotrophic and specific to Antarctic glaciers^70–73^. Cyanobacteria were a major part of the microbial community in both ornithogenic and non-ornithogenic cryoconites, contributing to the autotrophic component of the microbial community. Thus, the local origin of the cryoconite material contributed to the richness of the microbial community, but the main components were defined by the factors of the higher level. Cross-contamination by biotic and abiotic factors was also possible.

### The core microbiota of cryoconites

The famous saying by Baas Becking states: “Everything is everywhere, but the environment selects”^74^. This figure of speech, specifically about bacteria, refers to their biogeography. It has long been considered that bacteria are cosmopolitan organisms with no geographic barrier, with only environmental factors shaping their communities. But with the accumulation of high-throughput data on bacteria there appears to be evidence, which calls into question this idea^68,75^. Here we analyzed prokaryotic communities of similar habitats, but from distinct regions thousands of kilometers apart. After extensive filtering, aimed at reducing methodological contamination, only four ASVs from the whole dataset were identified as common for cryoconites from all regions—*Polaromonas* sp., *Rhodoferax* sp. (Betaproteobacteria), *Cryobacterium* sp. (Actinomycetota), and *Hymenobacter frigidus* (Bacteroidota). These genera were previously described as occurring in the glaciers, specifically in Eurasia^76,77^ and Antarctica^78^. *Polaromonas* and *Cryobacterium* were described in the literature as psychrophilic microorganisms^16,79^. *Polaromonas* representatives were reported to be globally distributed through air currents, which explains their presence in the locations remoted from the ocean^79^. These aeolian transfers can also act as a pre-selective medium of cryo-tolerant species since some microbes can survive in atmospheric water suspensions^9^. The occurrence of these common ASVs in both polar and non-polar regions, such as the northern Caucasus, indicates that they may have a broad ecological range and are not restricted to specific geographic locations. So, one could speculate that different bacteria have different biogeographic distribution potential.

## Conclusions

Prokaryotic communities of cryoconites were comprised of representatives of phyla, specific to this environment: Cyanobacteria and Chloroflexota as primary producers of organic carbon, Pseudomonadota as fast adapting copiotrophs/opportunists, Bacteroidota as consumers of various forms of carbon. However, remote locations facilitate the presence of distinct genera within these phyla, which can be explained both by geographical isolation and the variations in the chemical composition of the substrate. Each sampling site had its chemical composition portrait, however, they grouped by their relative geographical location—Arctic, Antarctic, and Caucasus. Microbiome composition reflected this distinction, but the differences were detected mostly on the lower taxonomic levels.

The combination of 16S rRNA gene sequencing and physicochemical analysis of cryoconite material allowed us to link differences in microbiome composition between remote glaciers to variations in available nutrient content. Chemical parameters defined variations in microbiome composition of cryoconites on several levels. On the global scale, microbiomes can be separated into regions with their defining chemical parameters, but on the level of ASV, we detected taxa, which correspond with analogous composition of chemical parameters across different locations. To conclude, even though on a large geographical scale the cryoconite microbiome composition is mostly shaped by chemical parameters of sediments, it is evident that geographical location and distance between sites also play a significant role. However, it is challenging to formalize this relationship.

### Supplementary Information

Supplementary Information 1.

Supplementary Information 2.

Supplementary Information 3.

## Data Availability

The amplicon sequencing data is available at SRA (NCBI, Bethesda, MD, USA) under the project ID PRJNA997912.
