# Supplementary material for: Linking the composition of cryoconite prokaryotic communities in the Arctic, Antarctic, and Central Caucasus with their chemical characteristics
Source: Sci Rep. 2024 Jul 9;14:15838. doi: 10.1038/s41598-024-64452-3 (PMC11233692; doi:10.1038/s41598-024-64452-3)
Supplement: Supplementary file 1 — Supplementary Information 1. [file 41598_2024_64452_MOESM1_ESM.html]

Linking the composition of cryoconite prokaryotic communities in the Arctic, Antarctic, and Central Caucasus with their chemical characteristics.


Code 

- Show All Code
- Hide All Code

# Linking the composition of cryoconite prokaryotic communities in the Arctic, Antarctic, and Central Caucasus with their chemical characteristics.

### Revision round 2

#### Grigory Gladkov

#### 2023-05-31

## 0.1 Preprocessing:

**Basic dada2 pipeline for 16S:**

truncLen=c(220, 160)  
trimLeft=c(19,20)  
maxEE=c(2,5)  
truncQ=2  
pool=pseudo  
database - SILVA 138.1  
  
Delete all bad taxa with parameters below:  
non assigned at the phylum level +  
chloroplasts/mitochondria +  
low reads/richness(based on the plot results)

## 0.2 Import

Import libraries and the previously generated phyloseq object

```
library(readxl)
library(tidyverse)
library(phyloseq)
library(tidyverse)
library(ggpubr)
library(ampvis2)
library(ANCOMBC)
library(heatmaply)
library(compositions)
library(WGCNA)
require(phyloseq)

ps.f2 <- readRDS("ps.f2")
```

### 0.2.1 Metadata preprocessing

```
ps.liv <- prune_samples(sample_data(ps.f2)$Group %in% "Pimpirev", ps.f2)
ps.liv  <- prune_taxa(taxa_sums(ps.liv) > 0, ps.liv)

ps.liv <- prune_samples(!sample_names(ps.liv) %in% paste0("Abakumov.Krio2.",seq(9, 12)), ps.liv)
ps.liv  <- prune_taxa(taxa_sums(ps.liv) > 0, ps.liv)

# maj rev 1st round SciRep
ps.f2 <- prune_samples(sample_data(ps.f2)$Cryoconite == "cryoconite", ps.f2)
ps.f2  <- prune_taxa(taxa_sums(ps.f2) > 0, ps.f2)


# ps.cr <- prune_samples(sample_data(ps.f2)$Cryoconite == "cryoconite", ps.f2)
# ps.cr  <- prune_taxa(taxa_sums(ps.cr) > 0, ps.cr)

ps.f2@sam_data <- read.csv('new.meta.csv') %>% 
  arrange(ID) %>% 
  column_to_rownames('ID') %>% 
  mutate_if(is.character, as.factor) %>% 
  mutate_if(is.integer, as.double) %>% 
  mutate(Region = fct_recode(Region, 
                             'arctic' = 'polar ural', 
                             'arctic' = 'severnaya zemlya', 
                             'antarctic' = 'antarctica')) %>% 
  mutate(Type=forcats::fct_relevel(Type, c("PAG", "PAOr" ,"Garabashi", "Shkelda","IGAN", "Mush"))) %>% 
  mutate(Region=forcats::fct_relevel(Region, c("antarctic" ,"caucasus","arctic"))) %>% 
  sample_data()

ps.f2@otu_table <- ps.f2@otu_table %>% 
  data.frame() %>% 
  rownames_to_column('ID') %>% 
  arrange(ID) %>% 
  column_to_rownames('ID') %>% 
  as.matrix() %>% 
  otu_table(taxa_are_rows = FALSE) 


ps.f2@tax_table <- ps.f2@tax_table@.Data %>% 
  data.frame() %>% 
  mutate(Phylum = str_replace_all(Phylum, c("Cyanobacteriota" = "Cyanobacteria"))) %>% 
  as.matrix() %>% 
  tax_table()


ps.cr <- ps.f2
```

## 0.3 Functions

```
plot_rich_reads_samlenames_lm <- function(physeq, group = "Site", label = "Repeat"){
  rish <- estimate_richness(physeq, measures = "Observed")
  reads.sum <- as.data.frame(sample_sums(physeq))
  reads.summary <- cbind(rish, reads.sum)
  colnames(reads.summary) <- c("otus","reads")
  reads.summary["Repeat"] <-unlist(purrr::map(stringr::str_split(rownames(physeq@sam_data), "\\.", 2), function(x) x[[2]]))
  reads.summary["Site"] <- physeq@sam_data[[group]]
  library(ggrepel)
  require(ggforce)
  p1 <- ggplot(data=reads.summary) + 
    geom_point(aes(y=otus, x=log2(reads), color=Site),size=3) + 
    geom_text_repel(aes(y=otus, x=log2(reads), label=paste0(Repeat))) + 
    theme_bw() +
    geom_smooth(aes(y=otus, x=log2(reads), fill=Site, color=Site),method=lm, se=FALSE, ymin = 1) + 
    scale_x_continuous(sec.axis = sec_axis(sec.axis ~ 2**.)) 

  return(p1)
}


beta_custom_norm_NMDS_elli_w <- function(ps, seed = 7888, normtype="vst", Color="What", Group="Repeat"){
  require(phyloseq)
  require(ggplot2)
  require(ggpubr)
  library(ggforce)
  
  ps@otu_table[ps@otu_table < 0] <- 0
  ordination.b <- ordinate(ps, "NMDS", "bray")
  mds <- as.data.frame(ordination.b$points)
  p  <-  plot_ordination(ps,
                         ordination.b,
                         type="sample",
                         color = Color,
                         title="NMDS - Bray-Curtis",
                         # title=NULL,
                         axes = c(1,2) ) + 
    theme_bw() + 
    theme(text = element_text(size = 10)) + 
    geom_point(size = 3) +
    annotate("text",
    x=min(mds$MDS1) + abs(min(mds$MDS1))/4,
    y=max(mds$MDS2),
    label=paste0("Stress -- ", round(ordination.b$stress, 3))) +
    geom_mark_ellipse(aes_string(group = Group, label = Group),
                      label.fontsize = 10,
                      label.buffer = unit(2, "mm"),
                      label.minwidth = unit(5, "mm"),
                      con.cap = unit(0.1, "mm"),
                      con.colour='gray') +
    theme(legend.position = "none") +
    scale_colour_viridis_d(option = "magma", 
                       aesthetics = "color", 
                       begin = 0, 
                       end = 0.8)
  
  return(p)
}


plot_alpha_w_toc_mod <- function(ps, group, metric) {
  
  require(phyloseq)
  require(ggplot2)
  
  ps_a <- prune_taxa(taxa_sums(ps) > 0, ps)
  
  er <- estimate_richness(ps_a)
  df_er <- cbind(ps_a@sam_data, er)
  df_er <- df_er %>% select(c(group, metric))
  stat.test <- aov(as.formula(paste0(metric, "~", group)), data = df_er) %>%
    rstatix::tukey_hsd() %>%
    filter(p.adj.signif != "ns")
  y <-  seq(max(er[[metric]]), length=length(stat.test$p.adj), by=max(er[[metric]]/20))

  plot_richness(ps_a, x=group, measures=metric, color="Region") + 
    geom_boxplot() +
    geom_point(size=1.2, alpha=0.3) +
    ggpubr::stat_pvalue_manual(
      stat.test, 
      label = "p.adj.signif", 
      y.position = y,
      tip.length = 0.005, 
      bracket.nudge.y = 1.5,
      vjust = 0.6, 
      size = 3) +
    theme_light() + 
        scale_colour_viridis_d(option = "magma", 
                       aesthetics = "color", 
                       begin = 0, 
                       end = 0.8) +
    theme(axis.text.x = element_text(angle = 45, hjust=1),
          axis.title.x=element_blank(),
          axis.title.y=element_blank(),
          legend.position = "None") +
    labs(y=paste(metric, "index")) 
}

phyloseq_to_ampvis2 <- function(physeq) {
  #check object for class
  if(!any(class(physeq) %in% "phyloseq"))
    stop("physeq object must be of class \"phyloseq\"", call. = FALSE)
  
  #ampvis2 requires taxonomy and abundance table, phyloseq checks for the latter
  if(is.null(physeq@tax_table))
    stop("No taxonomy found in the phyloseq object and is required for ampvis2", call. = FALSE)
  
  #OTUs must be in rows, not columns
  if(phyloseq::taxa_are_rows(physeq))
    abund <- as.data.frame(phyloseq::otu_table(physeq)@.Data)
  else
    abund <- as.data.frame(t(phyloseq::otu_table(physeq)@.Data))
  
  #tax_table is assumed to have OTUs in rows too
  tax <- phyloseq::tax_table(physeq)@.Data
  
  #merge by rownames (OTUs)
  otutable <- merge(
    abund,
    tax,
    by = 0,
    all.x = TRUE,
    all.y = FALSE,
    sort = FALSE
  )
  colnames(otutable)[1] <- "OTU"
  
  #extract sample_data (metadata)
  if(!is.null(physeq@sam_data)) {
    metadata <- data.frame(
      phyloseq::sample_data(physeq),
      row.names = phyloseq::sample_names(physeq), 
      stringsAsFactors = FALSE, 
      check.names = FALSE
    )
    
    #check if any columns match exactly with rownames
    #if none matched assume row names are sample identifiers
    samplesCol <- unlist(lapply(metadata, function(x) {
      identical(x, rownames(metadata))}))
    
    if(any(samplesCol)) {
      #error if a column matched and it's not the first
      if(!samplesCol[[1]])
        stop("Sample ID's must be in the first column in the sample metadata, please reorder", call. = FALSE)
    } else {
      #assume rownames are sample identifiers, merge at the end with name "SampleID"
      if(any(colnames(metadata) %in% "SampleID"))
        stop("A column in the sample metadata is already named \"SampleID\" but does not seem to contain sample ID's", call. = FALSE)
      metadata$SampleID <- rownames(metadata)
      
      #reorder columns so SampleID is the first
      metadata <- metadata[, c(which(colnames(metadata) %in% "SampleID"), 1:(ncol(metadata)-1L)), drop = FALSE]
    }
  } else
    metadata <- NULL
  
  #extract phylogenetic tree, assumed to be of class "phylo"
  if(!is.null(physeq@phy_tree)) {
    tree <- phyloseq::phy_tree(physeq)
  } else
    tree <- NULL
  
  #extract OTU DNA sequences, assumed to be of class "XStringSet"
  if(!is.null(physeq@refseq)) {
    #convert XStringSet to DNAbin using a temporary file (easiest)
    fastaTempFile <- tempfile(pattern = "ampvis2_", fileext = ".fa")
    Biostrings::writeXStringSet(physeq@refseq, filepath = fastaTempFile)
  } else
    fastaTempFile <- NULL
  
  #load as normally with amp_load
  ampvis2::amp_load(
    otutable = otutable,
    metadata = metadata,
    tree = tree,
    fasta = fastaTempFile
  )
}


detachAllPackages <- function() {

  basic.packages <- c("package:stats","package:graphics","package:grDevices","package:utils","package:datasets","package:methods","package:base")

  package.list <- search()[ifelse(unlist(gregexpr("package:",search()))==1,TRUE,FALSE)]

  package.list <- setdiff(package.list,basic.packages)

  if (length(package.list)>0)  for (package in package.list) detach(package, character.only=TRUE, force = TRUE)

}

get_list_from_ps <- function(physeq, amaz_fac) {
  my_keys <- levels(sample_data(physeq)[[amaz_fac]])
  mylist <- vector(mode="list", length=length(my_keys))
  names(mylist) <- my_keys
  for (i in levels(sample_data(physeq)[[amaz_fac]])) { 
    x <- prune_samples(sample_data(physeq)[[amaz_fac]] %in% i, physeq)
    x <- prune_taxa(taxa_sums(x) > 0, x)
    mylist[[i]] <- taxa_names(x)
  }
  return(mylist)
}

unregister <- function() {
  env <- foreach:::.foreachGlobals
  rm(list=ls(name=env), pos=env)
}


lsf.str()
```

```
## beta_custom_norm_NMDS_elli_w : function (ps, seed = 7888, normtype = "vst", Color = "What", Group = "Repeat")  
## detachAllPackages : function ()  
## get_list_from_ps : function (physeq, amaz_fac)  
## phyloseq_to_ampvis2 : function (physeq)  
## plot_alpha_w_toc_mod : function (ps, group, metric)  
## plot_rich_reads_samlenames_lm : function (physeq, group = "Site", label = "Repeat")  
## unregister : function ()
```

```
plot_rich_reads_samlenames_lm(ps.f2, group = "Group", label = "Group")
```

## 0.4 Alpha

```
ps.f2.r <- rarefy_even_depth(ps.f2, rngseed = 1312)

p_a1 <-  plot_alpha_w_toc_mod(ps.f2.r, group="Type", metric="Observed") 
p_a2 <- plot_alpha_w_toc_mod(ps.f2.r, group="Type", metric="Shannon") 
p_a3 <- plot_alpha_w_toc_mod(ps.f2.r, group="Type", metric="InvSimpson") 

p_alpha <- ggpubr::ggarrange(p_a1, p_a2, p_a3, nrow = 1)
# p_alpha <- ggpubr::ggarrange(p_a1, p_a3, nrow = 1)
p_alpha
```

```
estimate_richness(ps.f2) %>% 
  cbind2(sample_data(ps.f2) %>% 
           data.frame() %>% 
           dplyr::select(Type)) %>%
  relocate(Type) %>% 
  group_by(Type) %>%
  dplyr::select(c("Observed", "Shannon", "InvSimpson")) %>%
  summarise_all(mean) %>% 
  mutate_if(is.numeric, ~round(., digits = 2)) %>%
  DT::datatable(caption = "Alpha diversity metrics")
```

## 0.5 Beta

clr transformation from compositions library

https://www.rdocumentation.org/packages/compositions/versions/2.0-6/topics/clr

```
ps.f2.clr <- ps.f2
clr.f2.otu <- as.data.frame(t(ps.f2@otu_table)) %>% 
  compositions::clr() %>% 
  t()

ps.f2.clr@otu_table <-  otu_table(clr.f2.otu, taxa_are_rows = FALSE)

beta_all <- beta_custom_norm_NMDS_elli_w(ps.f2.clr, Group = "Type", Color = "Region") + 
  # labs(title = "A. all samples") + 
  labs(title = "NMDS - Bray-Curtis") +
          scale_colour_viridis_d(option = "magma", 
                       aesthetics = "color", 
                       begin = 0, 
                       end = 0.8)
```

```
## Run 0 stress 0.103213 
## Run 1 stress 0.1031834 
## ... New best solution
## ... Procrustes: rmse 0.003154436  max resid 0.01215111 
## Run 2 stress 0.1740892 
## Run 3 stress 0.1766943 
## Run 4 stress 0.162827 
## Run 5 stress 0.1398861 
## Run 6 stress 0.1420068 
## Run 7 stress 0.1746074 
## Run 8 stress 0.1881988 
## Run 9 stress 0.103213 
## ... Procrustes: rmse 0.00315464  max resid 0.01214528 
## Run 10 stress 0.1984924 
## Run 11 stress 0.1438684 
## Run 12 stress 0.1031837 
## ... Procrustes: rmse 0.00008327394  max resid 0.0006060514 
## ... Similar to previous best
## Run 13 stress 0.1731015 
## Run 14 stress 0.1804824 
## Run 15 stress 0.1753375 
## Run 16 stress 0.1609314 
## Run 17 stress 0.1691253 
## Run 18 stress 0.1215908 
## Run 19 stress 0.1607353 
## Run 20 stress 0.1831832 
## *** Best solution repeated 1 times
```

```
beta_all
```

### 0.5.1 heatmaps

phylum

```
amp_f2 <- phyloseq_to_ampvis2(ps.f2)


amp_heatmap(amp_f2,
            tax_show = 13,
            group_by = "Type",
            facet_by = "Region",
            tax_aggregate = "Phylum",
            tax_add = "Kingdom",
            normalise=TRUE,
            plot_values_size = 5.5,
            showRemainingTaxa = TRUE,
            color_vector = colorspace::lighten(viridis::magma(n = 3, begin = 0.35, direction = -1), 0.2)) +
            theme(axis.text.x = element_text(size=14, angle=45, hjust=0.4),
              axis.text.y = element_text(size=14))
```

Genus

```
amp_heatmap(amp_f2,
            tax_show = 40,
            group_by = "Type",
            facet_by = "Region",
            tax_aggregate = "Genus",
            tax_add = "Phylum",
            normalise=TRUE,
            plot_values_size = 4.5,
            showRemainingTaxa = TRUE,
            color_vector = colorspace::lighten(viridis::magma(n = 3, begin = 0.35, direction = -1), 0.2)) +
            theme(axis.text.x = element_text(size=14, angle=45, hjust=0.4),
              axis.text.y = element_text(size=13))
```

### 0.5.2 agrochemestry

Scaled heatmap - UPGMA clusteriasation based on the euclidian distances

```
ps.f2@sam_data %>% 
  data.frame() %>%
  # rownames_to_column("shit") %>% 
  # column_to_rownames("Name") %>% 
  dplyr::select(which(sapply(., is.numeric)), Type) %>% 
  dplyr::select(-c("lat", "long")) %>%
  group_by(Type) %>% 
  summarise(across(everything(), median),
            .groups = 'drop') %>% 
  column_to_rownames("Type") %>% 
  scale() %>% 
  heatmaply::ggheatmap(color = viridis::magma(n = 3, begin = 0.1, direction = -1), 
                       show_dendrogram = c(TRUE, FALSE), 
                       border=TRUE,
                       dist_method =  "euclidian", 
                       hclust_method = "average",
                       margins = c(1, 1, 1, 1))
```

### 0.5.3 Anova for agrochemestry

```
# library(multcomp)

met <- ps.f2@sam_data %>%
  data.frame() %>%
  dplyr::select(which(sapply(., is.numeric)), Type) %>%
  dplyr::select(-c("lat", "long")) %>%
  distinct(. , .keep_all = TRUE)
# # 
# mod <- nnet::multinom(data = met, Type ~ + .)
# mod <- glm(data = met, Type ~ + ., family = 'binomial')
# stats::anova(mod)
# res <- emmeans::emmeans(mod, ~Type)
# summary(res)
# pairs(res)
# 
# aov(data = met, Type ~ TOC + TON)
# 
# z <- summary(mod)$coefficients/summary(mod)$standard.errors
# p <- (1 - pnorm(abs(z), 0, 1)) * 2
# p
# stats::anova(mod, test='Chisq')
# met
# aov_res
# summary(aov_res)

# met %>% 
  # mutate_if(is.numeric, scale) %>%
  # pivot_longer(!Type, names_to='Agro', values_to='Vars') %>% 
  # group_by(Agro) %>%
  # rstatix::anova_test(Vars ~ Type) 
  
# aov(data = met, TOC ~ Type) %>% summary()  

met %>% 
  mutate_if(is.numeric, scale) %>%
  pivot_longer(!Type, names_to='Agro', values_to='Vars') %>% 
  rstatix::tukey_hsd(Vars ~ Type)
```

```
## # A tibble: 15 × 9
##    term  group1    group2    null.value estimate conf.low conf.high        p.adj
##  * <chr> <chr>     <chr>          <dbl>    <dbl>    <dbl>     <dbl>        <dbl>
##  1 Type  PAG       PAOr               0   0.836    0.432     1.24   0.0000000688
##  2 Type  PAG       Garabashi          0   0.575    0.171     0.979  0.000742    
##  3 Type  PAG       Shkelda            0  -0.0366  -0.479     0.406  1           
##  4 Type  PAG       IGAN               0   0.709    0.350     1.07   0.000000349 
##  5 Type  PAG       Mush               0   0.353    0.0211    0.684  0.0295      
##  6 Type  PAOr      Garabashi          0  -0.261   -0.665     0.142  0.435       
##  7 Type  PAOr      Shkelda            0  -0.873   -1.31     -0.430  0.000000358 
##  8 Type  PAOr      IGAN               0  -0.127   -0.486     0.232  0.915       
##  9 Type  PAOr      Mush               0  -0.483   -0.815    -0.152  0.000498    
## 10 Type  Garabashi Shkelda            0  -0.611   -1.05     -0.169  0.0012      
## 11 Type  Garabashi IGAN               0   0.135   -0.225     0.494  0.893       
## 12 Type  Garabashi Mush               0  -0.222   -0.554     0.110  0.395       
## 13 Type  Shkelda   IGAN               0   0.746    0.344     1.15   0.00000224  
## 14 Type  Shkelda   Mush               0   0.389    0.0117    0.767  0.0388      
## 15 Type  IGAN      Mush               0  -0.357   -0.632    -0.0809 0.00322     
## # ℹ 1 more variable: p.adj.signif <chr>
```

## 0.6 Pimpirev

A small stand-alone investigation of the ornitogenic and nonornitogenic samples from Antarctica

```
ancom_liv_asv <-  ANCOMBC::ancombc2(data = ps.liv, 
  fix_formula = "Type",
  rand_formula = NULL,
  p_adj_method = "fdr",
  pseudo = 0, 
  pseudo_sens = TRUE,
  prv_cut = 0.10,
  s0_perc = 0.05,
  group = "Type",
  struc_zero = TRUE,
  neg_lb = TRUE,
  alpha = 0.05,
  n_cl = 20, 
  tax_level = NULL,
  verbose = TRUE,
  global = TRUE, 
  pairwise = TRUE, 
  dunnet = FALSE, 
  trend = FALSE,
  iter_control = list(tol = 1e-2, max_iter = 20, verbose = TRUE),
  em_control = list(tol = 1e-5, max_iter = 100),
  lme_control = lme4::lmerControl(),
  mdfdr_control = list(fwer_ctrl_method = "holm", B = 100),
  trend_control = list(contrast = list(matrix(c(1, 0, -1, 1),
    nrow = 2,
    byrow = TRUE),
    matrix(c(-1, 0, 1, -1),
    nrow = 2,
    byrow = TRUE)),
   node = list(2, 2),
   solver = "ECOS",
  B = 100)
)
```

```
amp_liv <- phyloseq_to_ampvis2(ps.liv)

amp_heatmap(amp_liv,
            tax_show = 40,
            group_by = "Type",
            tax_aggregate = "Species",
            tax_add = "Genus",
            normalise=TRUE,
            showRemainingTaxa = TRUE)
```

```
p.venn.cc <- amp_venn(amp_liv,
         group_by = "Type",
         normalise = TRUE,
         cut_a = 0.000001,
         cut_f=0.000001
        )

p.venn.cc
```

Library normalization. The procedure is described in

http://www.bioconductor.org/packages/release/bioc/vignettes/ANCOMBC/inst/doc/ANCOMBC.html

chapter “Bias-corrected abundances”

An article with theory(the math is kind of sapplemented there):

Lin, H., Peddada, S.D. Analysis of compositions of microbiomes with bias correction. Nat Commun 11, 3514 (2020). https://doi.org/10.1038/s41467-020-17041-7  
ps.anc.liv - phyloseq object with corrected reads( and log-transformation also)  
  
use euclidean distance for plot

```
out.liv <-  ANCOMBC::ancombc2(data=ps.liv, 
  fix_formula = "Type",
  prv_cut = 0,
  p_adj_method = "BH",
  tax_level = NULL,
  rand_formula= NULL,
  pseudo = 0, 
  pseudo_sens = FALSE,
  s0_perc = 0.05,
  group = "Type",
  struc_zero = FALSE,
  neg_lb = TRUE,
  alpha = 0.05,
  n_cl = 20, 
  verbose = TRUE,
  global = FALSE, 
  pairwise = FALSE, 
  dunnet = FALSE, 
  trend = FALSE
  )
  
ps.anc.liv <- ps.liv 

samp_frac <-  out.liv$samp_frac
samp_frac[is.na(samp_frac)] <-  0 
# Add pesudo-count (1) to avoid taking the log of 0
log_obs_abn = log(out.liv$feature_table + 1)
# Adjust the log observed abundances
log_corr_abn = t(t(log_obs_abn) - samp_frac)
otu_table(ps.anc.liv) <- otu_table(t(log_corr_abn), taxa_are_rows = FALSE)

beta_custom_norm_NMDS_elli_w_eu <- function(ps, seed = 7888, normtype="vst", Color="What", Group="Repeat"){
  require(phyloseq)
  require(ggplot2)
  require(ggpubr)
  library(ggforce)
  
  ps@otu_table[ps@otu_table < 0] <- 0
  ordination.b <- ordinate(ps, "NMDS", "euclidean")
  mds <- as.data.frame(ordination.b$points)
  p  <-  plot_ordination(ps,
                         ordination.b,
                         type="sample",
                         color = Color,
                         title="NMDS - euclidean",
                         # title=NULL,
                         axes = c(1,2) ) + 
    theme_bw() + 
    theme(text = element_text(size = 10)) + 
    geom_point(size = 3) +
    annotate("text",
    x=min(mds$MDS1) + abs(min(mds$MDS1))/4,
    y=max(mds$MDS2),
    label=paste0("Stress -- ", round(ordination.b$stress, 3))) +
    geom_mark_ellipse(aes_string(group = Group, label = Group),
                      label.fontsize = 10,
                      label.buffer = unit(2, "mm"),
                      label.minwidth = unit(5, "mm"),
                      con.cap = unit(0.1, "mm"),
                      con.colour='gray') +
    theme(legend.position = "none") +
    scale_colour_viridis_d(option = "magma", 
                       aesthetics = "color", 
                       begin = 0, 
                       end = 0.8)
  
  return(p)
}

beta_custom_norm_NMDS_elli_w_eu(ps.anc.liv, Color = "Type", Group = "Type")
```

```
## Run 0 stress 0.0318379 
## Run 1 stress 0.0318379 
## ... New best solution
## ... Procrustes: rmse 0.000001449171  max resid 0.000003308049 
## ... Similar to previous best
## Run 2 stress 0.0315685 
## ... New best solution
## ... Procrustes: rmse 0.006169769  max resid 0.01591174 
## Run 3 stress 0.169102 
## Run 4 stress 0.0315685 
## ... Procrustes: rmse 0.000005193761  max resid 0.000008465896 
## ... Similar to previous best
## Run 5 stress 0.0318379 
## ... Procrustes: rmse 0.006170266  max resid 0.01592457 
## Run 6 stress 0.1713064 
## Run 7 stress 0.03168697 
## ... Procrustes: rmse 0.008960098  max resid 0.01982354 
## Run 8 stress 0.03168693 
## ... Procrustes: rmse 0.008968884  max resid 0.0198475 
## Run 9 stress 0.03147742 
## ... New best solution
## ... Procrustes: rmse 0.006138033  max resid 0.01747141 
## Run 10 stress 0.03147742 
## ... Procrustes: rmse 0.000002141618  max resid 0.000003669908 
## ... Similar to previous best
## Run 11 stress 0.03147742 
## ... New best solution
## ... Procrustes: rmse 0.0000003164085  max resid 0.0000006379316 
## ... Similar to previous best
## Run 12 stress 0.2061961 
## Run 13 stress 0.0315685 
## ... Procrustes: rmse 0.006137672  max resid 0.01746992 
## Run 14 stress 0.03168692 
## ... Procrustes: rmse 0.006575128  max resid 0.01706886 
## Run 15 stress 0.0315685 
## ... Procrustes: rmse 0.006136567  max resid 0.01746617 
## Run 16 stress 0.0315685 
## ... Procrustes: rmse 0.006136607  max resid 0.01746632 
## Run 17 stress 0.0318379 
## ... Procrustes: rmse 0.008552759  max resid 0.01703461 
## Run 18 stress 0.0315685 
## ... Procrustes: rmse 0.006138355  max resid 0.01747205 
## Run 19 stress 0.03168692 
## ... Procrustes: rmse 0.006575419  max resid 0.01707287 
## Run 20 stress 0.03168693 
## ... Procrustes: rmse 0.00657707  max resid 0.01707485 
## *** Best solution repeated 1 times
```

permanova

```
dist <- phyloseq::distance(ps.anc.liv, "euclidean")
metadata <- as(sample_data(ps.anc.liv@sam_data), "data.frame")
vegan::adonis2(dist ~ Type, data = metadata)
```

```
## Permutation test for adonis under reduced model
## Terms added sequentially (first to last)
## Permutation: free
## Number of permutations: 999
## 
## vegan::adonis2(formula = dist ~ Type, data = metadata)
##          Df SumOfSqs      R2      F Pr(>F)   
## Type      1   3112.4 0.25326 4.7483  0.002 **
## Residual 14   9176.8 0.74674                 
## Total    15  12289.3 1.00000                 
## ---
## Signif. codes:  0 '***' 0.001 '**' 0.01 '*' 0.05 '.' 0.1 ' ' 1
```

DA visualization

```
taxa_family <- ps.anc.liv %>% 
  tax_table() %>% 
  as.data.frame() %>%
  rownames_to_column("ID") %>% 
  add_column(abnd = taxa_sums(ps.anc.liv))

ancom_liv_asv$res %>% 
  filter(diff_TypePAOr == TRUE) %>% 
  select(c("taxon", "lfc_TypePAOr")) %>% 
  dplyr::rename("ID" = "taxon") %>% 
  left_join(taxa_family, by="ID") %>% 
  mutate(Phylum = case_when(Phylum %in% "Pseudomonadota" ~ Class,
                          !Phylum %in% "Pseudomonadota" ~ Phylum) %>% as.factor()) %>% 
  mutate(`more in` = ifelse(lfc_TypePAOr < 0, "PAG", "PAOr") %>% as.factor()) %>% 
  group_by(Genus, `more in`, Phylum ) %>% 
  summarise(area = sum(abnd)) %>% 
  mutate(Genus = str_replace_na(Genus, replacement = ' ')) %>% 
  ggplot(aes(area = area, 
             fill = `more in`, 
             subgroup = Phylum, 
             label = Genus)) +
  treemapify::geom_treemap() +
  treemapify::geom_treemap_subgroup_border(color = "white") +
  treemapify::geom_treemap_subgroup_text(place = "centre", 
                                         grow = T, 
                                         alpha = 0.5, 
                                         colour = "black", 
                                         fontface = "italic", 
                                         min.size = 0) +
  treemapify::geom_treemap_text(colour = "white", 
                                place = "topleft", 
                                grow = T, 
                                reflow = T, 
                                layout = 'squarified') +
  scale_colour_viridis_d(option = "magma", 
                         aesthetics = "fill", 
                         begin = 0.4, 
                         end = 0.8) +
  theme(legend.position="bottom")
```

## 0.7 Core

Venn without any filtrations  
code from https://github.com/a-zverev/16s-amplicon-processing

```
library(ggVennDiagram)

plot_vienn <- function(ps, group){
  physeq <- prune_taxa(taxa_sums(ps) > 0, ps)
  groups <- levels(sample_data(physeq)[[group]] %>% as.factor())
  data <- merge_samples(physeq, group) %>% 
    psmelt() %>% 
    group_by(Sample, OTU) %>% 
    summarise(ASVs_abund = list(paste(OTU, 1:sum(Abundance))), Abund = sum(Abundance), .groups='keep') %>% 
    filter(Abund > 0)
  
  asvs <- data %>% select(Sample, OTU) %>% group_by(Sample) %>% summarise(ASVs = list(OTU)) %>% as.list()
  d1 <- asvs[[2]]
  names(d1) <- asvs[[1]]
  d1
  
  weighted.asvs <- data %>% select(Sample, ASVs_abund) %>% group_by(Sample) %>% summarise(ASVs = list(unlist(ASVs_abund)))
  d2 <- weighted.asvs[[2]]
  names(d2) <- weighted.asvs[[1]]
  d2
  
  
  list(ggVennDiagram(d1) + ggtitle("ASVs") + scale_fill_distiller(palette = "OrRd", trans = "reverse"),
       ggVennDiagram(d2) + ggtitle("Reads") + scale_fill_distiller(palette = "OrRd", trans = "reverse"))
}

plot_vienn(ps.f2.r, "Region")
```

```
## [[1]]
```

```
## 
## [[2]]
```

Three sampling region core phylotypes.

```
physeq <- ps.cr
l <- get_list_from_ps(physeq, "Region")
core <- Reduce(intersect, l)
ps.core <- prune_taxa(core, ps.cr)
ps.core
```

```
## phyloseq-class experiment-level object
## otu_table()   OTU Table:         [ 356 taxa and 68 samples ]
## sample_data() Sample Data:       [ 68 samples by 21 sample variables ]
## tax_table()   Taxonomy Table:    [ 356 taxa by 7 taxonomic ranks ]
```

Find structural zeros based on function with ancom-bc package

```
ancom_core <-  ancombc2(data = ps.core, 
  fix_formula = "Region",
  rand_formula = NULL,
  p_adj_method = "fdr",
  pseudo_sens = TRUE,
  prv_cut = 0.05,
  s0_perc = 0.05,
  group = "Region",
  struc_zero = TRUE,
  neg_lb = TRUE,
  alpha = 0.05,
  n_cl = 15,
  lib_cut = FALSE,
  tax_level = NULL,
  verbose = TRUE,
  global = FALSE, 
  pairwise = FALSE, 
  dunnet = FALSE, 
  trend = FALSE
 )
```

Only 32 phylotypes remain(from 152) for the core community after filtering based on the concept of structural zeros.

```
non_str_otu <- ancom_core$res$taxon
          

zeros <- ancom_core$zero_ind %>% 
  column_to_rownames("taxon") %>% 
  rowSums() > 0L

zeros.all <- zeros[zeros == FALSE]
zeros.all <- names(zeros.all)

zeros.all %>% 
  length()
```

```
## [1] 62
```

Bottom of the list - Lactobacillus apis, Frischella perrara, Snodgrassella alvi - bees gut endosymbionts, so maybe contamination from another dataset (try to find in documentation for our Illumina Miseq run something related to bees, honey of something - didn’t find anything specific, but I think glaciers and the Antarctic are not regular vomiting places for bees).  
At the top of the list - the “true core”. Validate through https://www.gbif.org/ databases whether these phylotypes are really typical for glaciers. Eliminate phylotypes that look like a cross-contamination from different samples (like Seq1 (I think in real it was only in Mushketova place), Seq35 (Pipmperev reads looks like error) etc.) I think in future I should make threshold in structural zeros a little bit more strict (but I should double check this).  
So I’m going to select only 6 ASVs sequentially: Polaromonas sp.(Seq16 and Seq62), Cryobacterium sp.(Seq82), Rhodoferax sp.(Seq17) and Hymenobacter frigidus(Seq81). This is quite subjective, but I’m of the opinion that regular aplicon sequencing is like a leaky sieve. And I have some articles that prove that -  
https://doi.org/10.1128/msystems.00186-19  
https://doi.org/10.1128%2FmBio.00598-21

add also 2 ASVs of the Pajaroellobacter(Seq251, Seq124)

heatmap and species names for validation

```
ps.core.ancm <- prune_taxa(zeros.all, ps.core)
amp.core.ancm <- phyloseq_to_ampvis2(ps.core.ancm)

amp.core.ancm$tax %>% 
  select(c(Genus, Species))
```

```
##                               Genus          Species
## Seq1         Phormidesmis_ANT.L52.6       priestleyi
## Seq3        Tychonema_CCAP_1459-11B                 
## Seq4               Phormidium_CYN64                 
## Seq5         Phormidesmis_ANT.L52.6                 
## Seq6                   Granulicella                 
## Seq7        Tychonema_CCAP_1459-11B                 
## Seq8           Parafrigoribacterium      amurskyense
## Seq10           Crinalium_SAG_22.89                 
## Seq12                  Acidiphilium                 
## Seq13                                               
## Seq16                   Polaromonas                 
## Seq17                    Rhodoferax                 
## Seq18                                               
## Seq21                 Blastocatella                 
## Seq23                     Solitalea                 
## Seq24   Clostridium_sensu_stricto_9                 
## Seq27                   Thermomonas                 
## Seq28                   Pseudomonas                 
## Seq31         Candidatus_Solibacter                 
## Seq33                Aquipuribacter                 
## Seq35               Ferruginibacter                 
## Seq36                                               
## Seq40               Ferruginibacter                 
## Seq41                   Polaromonas                 
## Seq43                 Cutibacterium                 
## Seq46                                               
## Seq48                  Sphingomonas           jaspsi
## Seq49                  Sphingomonas                 
## Seq51                                               
## Seq53                 Brevundimonas                 
## Seq57                                               
## Seq59                    Variovorax                 
## Seq60                                               
## Seq62                   Polaromonas                 
## Seq67                         AAP99                 
## Seq74                  Gemmatimonas                 
## Seq76                  Acidiphilium                 
## Seq80                                 longiquaesitum
## Seq81                  Hymenobacter         frigidus
## Seq82                 Cryobacterium                 
## Seq89                                               
## Seq124             Pajaroellobacter                 
## Seq139                                              
## Seq166                 Hymenobacter                 
## Seq251             Pajaroellobacter                 
## Seq309                     Massilia eurypsychrophila
## Seq334         Candidatus_Nitrotoga                 
## Seq538                Lactobacillus                 
## Seq548                Lactobacillus                 
## Seq864                Snodgrassella             alvi
## Seq877                   Frischella          perrara
## Seq885                                              
## Seq987                                              
## Seq1123               Lactobacillus             apis
## Seq1137               Lactobacillus                 
## Seq1236   Candidatus_Nitrososphaera                 
## Seq1426                  Arcobacter         butzleri
## Seq1430               Fimbriiglobus                 
## Seq1625               Lactobacillus        johnsonii
## Seq1683                 Megasphaera                 
## Seq1701                 Acetobacter      lovaniensis
## Seq2109                  Prevotella
```

```
amp_heatmap(amp.core.ancm,
            tax_show = 155,
            tax_aggregate = "OTU",
            tax_add = "Genus",
            normalise=FALSE, 
            plot_values_size = 2, 
            facet_by = "Region",
            showRemainingTaxa = TRUE)
```

## 0.8 Phylums DA

```
ancom_cr_phylum <-  ANCOMBC::ancombc2(data = ps.cr, 
  tax_level = "Phylum",
  fix_formula = "Type",
  rand_formula = NULL,
  p_adj_method = "fdr",
  pseudo = 0, 
  pseudo_sens = TRUE,
  prv_cut = 0.10,
  s0_perc = 0.05,
  group = "Type",
  struc_zero = TRUE,
  neg_lb = TRUE,
  alpha = 0.05,
  n_cl = 20, 
  verbose = TRUE,
  global = TRUE, 
  pairwise = TRUE, 
  dunnet = TRUE, 
  trend = FALSE,
  iter_control = list(tol = 1e-2, max_iter = 20, verbose = TRUE),
  em_control = list(tol = 1e-5, max_iter = 100),
  lme_control = lme4::lmerControl(),
  mdfdr_control = list(fwer_ctrl_method = "holm", B = 100),
  trend_control = list(contrast = list(matrix(c(1, 0, -1, 1),
    nrow = 2,
    byrow = TRUE),
    matrix(c(-1, 0, 1, -1),
    nrow = 2,
    byrow = TRUE)),
   node = list(2, 2),
   solver = "ECOS",
  B = 100)
  )
```

```
ancom_cr_phylum$res_pair %>% 
  select("taxon"|starts_with("p_")) %>% 
  rename_all(~ stringr::str_replace(., regex("p_Type", ignore_case = TRUE), "")) %>% 
  rename_all(~ stringr::str_replace(., regex("Type", ignore_case = TRUE), "")) %>% 
  rename_if( !stringr::str_detect(names(.), "_") & !stringr::str_detect(names(.), "taxon"),  ~ paste0(., "_PAG")) %>%  
  reshape2::melt() %>% 
  rstatix::p_format(value, new.col = TRUE, digits = 2, accuracy = 1e-02) %>% 
  mutate(value.format = ifelse(value.format == "1", "", value.format)) %>%
  separate(variable,into=c('Var1', 'Var2'), sep = "_") %>% 
  mutate_if(is.character, as.factor) %>% 
  mutate_at(c('Var1', 'Var2'), ~forcats::fct_relevel(., c("PAG", "PAOr" ,"Garabashi", "Shkelda","IGAN", "Mush"))) %>% 
  ggplot(aes(Var1, Var2, fill = value)) + 
  geom_tile() +
  scale_fill_gradientn(colours = c("#A5317EFF", "#FCFDBFFF",  "#FCFDBFFF" ),
                       values = scales::rescale(c(0, 0.05, 1))) +
  geom_text(aes(label = value.format)) +
  facet_wrap(~taxon) +
  theme_bw() +
  theme(legend.position = "none",
          axis.title.x=element_blank(),
          axis.title.y=element_blank())
```

## 0.9 WGCNA

I’m trying to do variance stabilization with DESeq2 and clr from compositions before using the ancom-bc based normalization. Some of the intermediate plots are shown below.

vst

A caption

clr

### 0.9.1 ancom-bc

Filtration of the minor phylotypes

```
ps.cr.f <- phyloseq::filter_taxa(ps.cr, function(x) sum(x > 10) > (0.1*length(x)), TRUE)
pruned <- setdiff(phyloseq::taxa_names(ps.cr.f), c("Seq396", "Seq321", "Seq66"))
ps.cr.f <- phyloseq::prune_taxa(pruned, ps.cr.f)
full.sum <- ps.cr %>% phyloseq::sampleSums() %>% sum()
filtered.sum <- ps.cr.f %>% 
  phyloseq::sampleSums() %>% 
  sum()
paste0('percent of remaining reads -- ', round(filtered.sum / full.sum * 100, digits = 2) , '%')
```

```
## [1] "percent of remaining reads -- 86.36%"
```

```
ps.core.ancm <- prune_taxa(zeros.all, ps.core)
amp.core.ancm <- phyloseq_to_ampvis2(ps.core.ancm)

# amp.core.ancm$tax %>% 
#   select(c(Genus, Species))

# pruned <- setdiff(phyloseq::taxa_names(ps.cr.f), major_taxa)
# major_taxa <- taxa_names(ps.cr.f)
# ps.cr.remains <- phyloseq::prune_taxa(pruned, ps.cr.f)
# amp.remains <- phyloseq_to_ampvis2(ps.cr.remains)
# amp_heatmap(amp.remains,
#             tax_show = 60,
#             tax_aggregate = "OTU",
#             tax_add = "Genus",
#             normalise=FALSE, 
#             plot_values_size = 2, 
#             facet_by = "Region",
            # showRemainingTaxa = TRUE)
```

```
out.f <-  ANCOMBC::ancombc2(data=ps.cr.f, 
  fix_formula = "Type",
  prv_cut = 0,
  p_adj_method = "BH",
  tax_level = NULL,
  rand_formula= NULL,
  pseudo = 0, 
  pseudo_sens = FALSE,
  s0_perc = 0.05,
  group = "Type",
  struc_zero = FALSE,
  neg_lb = TRUE,
  alpha = 0.05,
  n_cl = 20, 
  verbose = TRUE,
  global = FALSE, 
  pairwise = FALSE, 
  dunnet = FALSE, 
  trend = FALSE
  )
```

```
ps.anc.f <- ps.cr.f

samp_frac <-  out.f$samp_frac
samp_frac[is.na(samp_frac)] <-  0 
# Add pesudo-count (1) to avoid taking the log of 0
log_obs_abn = log(out.f$feature_table + 1)
# Adjust the log observed abundances
log_corr_abn = t(t(log_obs_abn) - samp_frac)
otu_table(ps.anc.f) <- otu_table(t(log_corr_abn), taxa_are_rows = FALSE)

beta_custom_norm_NMDS_elli_w(ps.anc.f, Color = "Type", Group = "Type")
```

```
## Run 0 stress 0.06790316 
## Run 1 stress 0.06790327 
## ... Procrustes: rmse 0.0002316303  max resid 0.0004157164 
## ... Similar to previous best
## Run 2 stress 0.06790021 
## ... New best solution
## ... Procrustes: rmse 0.000633473  max resid 0.00325028 
## ... Similar to previous best
## Run 3 stress 0.06790339 
## ... Procrustes: rmse 0.0007255176  max resid 0.003129516 
## ... Similar to previous best
## Run 4 stress 0.068052 
## ... Procrustes: rmse 0.00390033  max resid 0.02072565 
## Run 5 stress 0.06803883 
## ... Procrustes: rmse 0.003840723  max resid 0.02086128 
## Run 6 stress 0.06790335 
## ... Procrustes: rmse 0.0007275566  max resid 0.003131965 
## ... Similar to previous best
## Run 7 stress 0.06801183 
## ... Procrustes: rmse 0.002914615  max resid 0.02069643 
## Run 8 stress 0.06790354 
## ... Procrustes: rmse 0.000754077  max resid 0.003168094 
## ... Similar to previous best
## Run 9 stress 0.06799474 
## ... Procrustes: rmse 0.004747574  max resid 0.02118683 
## Run 10 stress 0.06790331 
## ... Procrustes: rmse 0.0007196867  max resid 0.00312038 
## ... Similar to previous best
## Run 11 stress 0.0679002 
## ... New best solution
## ... Procrustes: rmse 0.000380921  max resid 0.0006825981 
## ... Similar to previous best
## Run 12 stress 0.06790336 
## ... Procrustes: rmse 0.0006337674  max resid 0.003362514 
## ... Similar to previous best
## Run 13 stress 0.06803865 
## ... Procrustes: rmse 0.003728318  max resid 0.0211827 
## Run 14 stress 0.06801183 
## ... Procrustes: rmse 0.002904344  max resid 0.02100181 
## Run 15 stress 0.06790308 
## ... Procrustes: rmse 0.0006532177  max resid 0.003449188 
## ... Similar to previous best
## Run 16 stress 0.0680118 
## ... Procrustes: rmse 0.002906004  max resid 0.02100542 
## Run 17 stress 0.06790341 
## ... Procrustes: rmse 0.0007298839  max resid 0.003563363 
## ... Similar to previous best
## Run 18 stress 0.06789993 
## ... New best solution
## ... Procrustes: rmse 0.0002847404  max resid 0.0005102431 
## ... Similar to previous best
## Run 19 stress 0.06801179 
## ... Procrustes: rmse 0.002905255  max resid 0.02078559 
## Run 20 stress 0.06803869 
## ... Procrustes: rmse 0.003825535  max resid 0.02106927 
## *** Best solution repeated 1 times
```

```
beta_custom_norm_NMDS_elli_w(ps.cr.f, Color = "Type", Group = "Type")
```

```
## Square root transformation
## Wisconsin double standardization
## Run 0 stress 0.05288792 
## Run 1 stress 0.05288789 
## ... New best solution
## ... Procrustes: rmse 0.00003022964  max resid 0.0001114721 
## ... Similar to previous best
## Run 2 stress 0.08997526 
## Run 3 stress 0.07679123 
## Run 4 stress 0.05288764 
## ... New best solution
## ... Procrustes: rmse 0.0001196251  max resid 0.0006860119 
## ... Similar to previous best
## Run 5 stress 0.05288787 
## ... Procrustes: rmse 0.0001189985  max resid 0.000681166 
## ... Similar to previous best
## Run 6 stress 0.05288788 
## ... Procrustes: rmse 0.0001206722  max resid 0.0006851817 
## ... Similar to previous best
## Run 7 stress 0.05288765 
## ... Procrustes: rmse 0.00007611304  max resid 0.0001475476 
## ... Similar to previous best
## Run 8 stress 0.09284606 
## Run 9 stress 0.05288761 
## ... New best solution
## ... Procrustes: rmse 0.00001897237  max resid 0.00006366463 
## ... Similar to previous best
## Run 10 stress 0.0528879 
## ... Procrustes: rmse 0.0001173259  max resid 0.0006868257 
## ... Similar to previous best
## Run 11 stress 0.05288761 
## ... New best solution
## ... Procrustes: rmse 0.0000210739  max resid 0.00004576 
## ... Similar to previous best
## Run 12 stress 0.08923648 
## Run 13 stress 0.05288789 
## ... Procrustes: rmse 0.0001207154  max resid 0.0006901036 
## ... Similar to previous best
## Run 14 stress 0.05288763 
## ... Procrustes: rmse 0.00002606269  max resid 0.00006170425 
## ... Similar to previous best
## Run 15 stress 0.05288793 
## ... Procrustes: rmse 0.0001235248  max resid 0.0006932123 
## ... Similar to previous best
## Run 16 stress 0.05292753 
## ... Procrustes: rmse 0.0009497226  max resid 0.005993469 
## ... Similar to previous best
## Run 17 stress 0.0528879 
## ... Procrustes: rmse 0.0001233393  max resid 0.0006967316 
## ... Similar to previous best
## Run 18 stress 0.05292745 
## ... Procrustes: rmse 0.0009409889  max resid 0.005897167 
## ... Similar to previous best
## Run 19 stress 0.05288787 
## ... Procrustes: rmse 0.0001165102  max resid 0.0006840957 
## ... Similar to previous best
## Run 20 stress 0.09319418 
## *** Best solution repeated 8 times
```

```
# ps.anc.f@otu_table %>%  
  # data.frame()
```

```
beta_custom_norm_NMDS_elli_w_species <- function(ps, seed = 7888, normtype="vst", Color="What", Group="Repeat"){
  require(phyloseq)
  require(ggplot2)
  require(ggpubr)
  library(ggforce)
  
  ps@otu_table[ps@otu_table < 0] <- 0
  ordination.b <- ordinate(ps, "NMDS", "bray")
  mds <- as.data.frame(ordination.b$points)
  p  <-  plot_ordination(ps,
                         ordination.b,
                         type="species",
                         color = Color,
                         title="NMDS - Bray-Curtis",
                         # title=NULL,
                         axes = c(1,2) ) + 
    theme_bw() + 
    theme(text = element_text(size = 10)) + 
    geom_point(size = 3) +
    annotate("text",
    x=min(mds$MDS1) + abs(min(mds$MDS1))/4,
    y=max(mds$MDS2),
    label=paste0("Stress -- ", round(ordination.b$stress, 3))) +
    # geom_mark_ellipse(aes_string(group = Group, label = Group),
    #                   label.fontsize = 10,
    #                   label.buffer = unit(2, "mm"),
    #                   label.minwidth = unit(5, "mm"),
    #                   con.cap = unit(0.1, "mm"),
    #                   con.colour='gray') +
    theme(legend.position = "none") +
    scale_colour_viridis_d(option = "magma", 
                       aesthetics = "color", 
                       begin = 0, 
                       end = 0.8)
  
  return(p)
}
print('All data without normalisation - samples')
```

```
## [1] "All data without normalisation - samples"
```

```
beta_custom_norm_NMDS_elli_w(ps.f2, Color = "Type", Group = "Type")
```

```
## Square root transformation
## Wisconsin double standardization
## Run 0 stress 0.07228812 
## Run 1 stress 0.1295749 
## Run 2 stress 0.1722082 
## Run 3 stress 0.07228812 
## ... New best solution
## ... Procrustes: rmse 0.000002739895  max resid 0.00001041138 
## ... Similar to previous best
## Run 4 stress 0.08291397 
## Run 5 stress 0.07231191 
## ... Procrustes: rmse 0.001696986  max resid 0.01124713 
## Run 6 stress 0.1720177 
## Run 7 stress 0.1824404 
## Run 8 stress 0.1295749 
## Run 9 stress 0.1555837 
## Run 10 stress 0.1295744 
## Run 11 stress 0.07228812 
## ... New best solution
## ... Procrustes: rmse 0.000003083987  max resid 0.000008447897 
## ... Similar to previous best
## Run 12 stress 0.1332776 
## Run 13 stress 0.1249558 
## Run 14 stress 0.07228812 
## ... New best solution
## ... Procrustes: rmse 0.000004479461  max resid 0.00002645979 
## ... Similar to previous best
## Run 15 stress 0.1824256 
## Run 16 stress 0.07231191 
## ... Procrustes: rmse 0.001694612  max resid 0.0112246 
## Run 17 stress 0.07231191 
## ... Procrustes: rmse 0.001696386  max resid 0.01124307 
## Run 18 stress 0.07287333 
## Run 19 stress 0.182326 
## Run 20 stress 0.08475573 
## *** Best solution repeated 1 times
```

```
print('Clr transformation - samples - seen the difference in library size differencies')
```

```
## [1] "Clr transformation - samples - seen the difference in library size differencies"
```

```
beta_custom_norm_NMDS_elli_w(ps.f2.clr, Color = "Type", Group = "Type")
```

```
## Run 0 stress 0.103213 
## Run 1 stress 0.103213 
## ... Procrustes: rmse 0.000002082615  max resid 0.000009673123 
## ... Similar to previous best
## Run 2 stress 0.1031827 
## ... New best solution
## ... Procrustes: rmse 0.003028406  max resid 0.01129787 
## Run 3 stress 0.1031827 
## ... Procrustes: rmse 0.000003749846  max resid 0.00002278174 
## ... Similar to previous best
## Run 4 stress 0.1713079 
## Run 5 stress 0.1215347 
## Run 6 stress 0.1826783 
## Run 7 stress 0.1703779 
## Run 8 stress 0.1215347 
## Run 9 stress 0.1739705 
## Run 10 stress 0.1390072 
## Run 11 stress 0.1400439 
## Run 12 stress 0.1032131 
## ... Procrustes: rmse 0.003026459  max resid 0.01127783 
## Run 13 stress 0.1685913 
## Run 14 stress 0.1215347 
## Run 15 stress 0.1993888 
## Run 16 stress 0.103213 
## ... Procrustes: rmse 0.003025565  max resid 0.01126895 
## Run 17 stress 0.1733321 
## Run 18 stress 0.1690759 
## Run 19 stress 0.1544427 
## Run 20 stress 0.190039 
## *** Best solution repeated 1 times
```

```
print('Clr transformation - species')
```

```
## [1] "Clr transformation - species"
```

```
beta_custom_norm_NMDS_elli_w_species(ps.f2.clr, Color = "Type", Group = "Type")
```

```
## Run 0 stress 0.103213 
## Run 1 stress 0.1216268 
## Run 2 stress 0.1031827 
## ... New best solution
## ... Procrustes: rmse 0.003026727  max resid 0.01128572 
## Run 3 stress 0.1398861 
## Run 4 stress 0.1544427 
## Run 5 stress 0.1758735 
## Run 6 stress 0.1633073 
## Run 7 stress 0.1031829 
## ... Procrustes: rmse 0.00006664842  max resid 0.0004443611 
## ... Similar to previous best
## Run 8 stress 0.1031828 
## ... Procrustes: rmse 0.00008827703  max resid 0.0006266914 
## ... Similar to previous best
## Run 9 stress 0.1031991 
## ... Procrustes: rmse 0.001905151  max resid 0.009848086 
## ... Similar to previous best
## Run 10 stress 0.1730947 
## Run 11 stress 0.1399037 
## Run 12 stress 0.1747909 
## Run 13 stress 0.1702673 
## Run 14 stress 0.1390587 
## Run 15 stress 0.1031836 
## ... Procrustes: rmse 0.0003781877  max resid 0.00272133 
## ... Similar to previous best
## Run 16 stress 0.1031827 
## ... Procrustes: rmse 0.000007456936  max resid 0.00005172567 
## ... Similar to previous best
## Run 17 stress 0.1620625 
## Run 18 stress 0.1880639 
## Run 19 stress 0.1215908 
## Run 20 stress 0.1702136 
## *** Best solution repeated 5 times
```

```
ps.vst.mod <- ps.anc.f
ps.vst.mod@sam_data <- ps.vst.mod@sam_data %>% 
  data.frame() %>% 
  mutate(Repeat = paste0(Group, "_", sapply(str_split(row.names(.), '\\.', 3), function(x) x[[3]]))
                         ) %>% 
  sample_data()

data3 <- ps.vst.mod@otu_table@.Data %>% 
  as.data.frame()

rownames(data3) <- as.character(ps.vst.mod@sam_data$Repeat)
powers <-  c(seq(from = 1, to=10, by=0.5), seq(from = 11, to=20, by=1))
unregister()
sft3 <-  pickSoftThreshold(data3, powerVector = powers, verbose = 5, networkType = "signed hybrid")
```

```
## pickSoftThreshold: will use block size 716.
##  pickSoftThreshold: calculating connectivity for given powers...
##    ..working on genes 1 through 716 of 716
##    Power SFT.R.sq   slope truncated.R.sq mean.k. median.k. max.k.
## 1    1.0   0.7060  2.3600          0.788  123.00    126.00  177.0
## 2    1.5   0.8590  1.8000          0.840   96.60     93.80  142.0
## 3    2.0   0.8100  1.0600          0.909   79.00     75.50  132.0
## 4    2.5   0.5500  0.7180          0.932   66.60     63.70  124.0
## 5    3.0   0.1070  0.2300          0.663   57.40     55.30  117.0
## 6    3.5   0.0102 -0.0625          0.542   50.40     48.80  112.0
## 7    4.0   0.2170 -0.2790          0.584   44.80     42.80  106.0
## 8    4.5   0.4660 -0.4230          0.700   40.20     37.50  102.0
## 9    5.0   0.6180 -0.5270          0.749   36.50     33.20   97.6
## 10   5.5   0.6850 -0.5940          0.763   33.30     29.40   93.8
## 11   6.0   0.7330 -0.6690          0.756   30.60     26.00   90.3
## 12   6.5   0.6870 -0.7370          0.685   28.30     23.20   87.1
## 13   7.0   0.7320 -0.7520          0.725   26.30     20.90   84.2
## 14   7.5   0.8050 -0.7720          0.816   24.50     19.00   81.5
## 15   8.0   0.8140 -0.8090          0.814   23.00     17.30   78.9
## 16   8.5   0.8690 -0.8100          0.884   21.60     15.80   76.5
## 17   9.0   0.8910 -0.8260          0.902   20.30     14.40   74.3
## 18   9.5   0.8970 -0.8410          0.903   19.20     13.20   72.2
## 19  10.0   0.8860 -0.8610          0.885   18.20     12.10   70.3
## 20  11.0   0.7770 -0.9020          0.733   16.40     10.40   66.7
## 21  12.0   0.8010 -0.9000          0.767   14.90      8.92   63.5
## 22  13.0   0.9120 -0.8860          0.922   13.60      7.79   60.6
## 23  14.0   0.9330 -0.9100          0.948   12.50      6.74   57.9
## 24  15.0   0.9520 -0.9230          0.981   11.60      5.91   55.5
## 25  16.0   0.9490 -0.9480          0.973   10.70      5.14   53.2
## 26  17.0   0.9460 -0.9410          0.973   10.00      4.52   51.2
## 27  18.0   0.9300 -0.9700          0.952    9.33      4.00   49.3
## 28  19.0   0.9010 -0.9840          0.921    8.74      3.58   47.5
## 29  20.0   0.9020 -0.9930          0.923    8.20      3.17   45.8
```

```
plot(sft3$fitIndices[,1], -sign(sft3$fitIndices[,3])*sft3$fitIndices[,2], xlab="Soft Threshold (power)",ylab="Scale Free Topology Model Fit,signed R^2",type="n", main = paste("Scale independence"))
text(sft3$fitIndices[,1], -sign(sft3$fitIndices[,3])*sft3$fitIndices[,2], labels=powers,cex=0.9,col="red")
abline(h=0.9,col="salmon")
```

13

```
detachAllPackages()
library(WGCNA)


net3 <- WGCNA::blockwiseModules(data3,
                          power=13,
                          TOMType="signed",
                          networkType="signed hybrid",
                          nThreads=15)

library(phyloseq)
library(tidyverse)
library(ggpubr)
library(ampvis2)
library(heatmaply)
library(WGCNA)
library(phyloseq)
library(ggtree)
library(tidyverse)
library(KneeArrower)

mergedColors2 <- net3$colors

plotDendroAndColors(
  net3$dendrograms[[1]],
  mergedColors2[net3$blockGenes[[1]]],
  "Module colors",
  dendroLabels = FALSE,
  hang = 0.03,
  addGuide = TRUE,
  guideHang = 0.05)
```

```
ids <- ps.anc.f@sam_data %>% 
  data.frame() %>%
  rownames_to_column("ID") %>% 
  # filter(Region == "Yakutsk") %>%
  pull(ID)
  

agra_data <- ps.anc.f@sam_data %>% 
  data.frame() %>% 
  select_if(., is.numeric) %>% 
  select( -c("long", "lat")) %>% 
  relocate(pH, P2O5) %>% 
  relocate(Ni, .after = N.NO3) %>% 
  relocate(CN, .after = Cd)

# mergedColors2 %>% unique()
nOTUs <- ncol(data3)
nSamples <- nrow(data3)

# Recalculate MEs with color labels
MEs0 <- moduleEigengenes(data3, mergedColors2)$eigengenes
MEs <- orderMEs(MEs0)

names(MEs) <- substring(names(MEs), 3)
names(MEs) <- c("black", "blue","green", "pink", "red", "turq.", "brown", "yellow", "grey")  

moduleTraitCor <- cor(MEs, agra_data, use = "p")
moduleTraitPvalue = corPvalueStudent(moduleTraitCor, nSamples)

# PLOT
sizeGrWindow(10,6)
textMatrix <- paste(signif(moduleTraitCor, 2), "\n(", format.pval(moduleTraitPvalue, digits = 2, eps = 0.001, nsmall = 3), ")", sep = "")
dim(textMatrix) <- dim(moduleTraitCor)
par(mar = c(4, 10, 2, 2))

# Display the correlation values within a heatmap
labeledHeatmap(Matrix = moduleTraitCor, 
               xLabels = names(agra_data),
               yLabels = names(MEs), 
               ySymbols = names(MEs), 
               font.lab.y = 2,
               font.lab.x = 2,
               # yLabelsPosition = "right",
               cex.lab.y = 0.85,
               colorLabels = FALSE, 
               colors = blueWhiteRed(50),
               textMatrix = textMatrix, 
               setStdMargins = FALSE,
               cex.text = 0.8, 
               zlim = c(-1,1), 
               plotLegend = FALSE,
               main = paste("Correlation of the WGCNA clusters with soil characteristics"))
```

```
vegan::inertcomp(cca_w_varstab_asv, display = c("species"), proportional = TRUE)
```

```
vegan::goodness(cca_w_varstab_asv, display='species', statistic = c("explained"), model='cca', proportional=TRUE, summarize=TRUE)
```

## 0.10 cca

### 0.10.1 groups

Select “groups” from WGCNA clusters

PH - caucasian - “brown”, “yellow”  
ME - arctic - “black”, “blue”, “green”, “pink”, “red”  
CN - antarctic - “turquoise”  
“grey” cluster dropped

```
library(ggrepel)

ps.rel  <-  phyloseq::transform_sample_counts(ps.cr, function(x) x / sum(x) * 100)

clust.ph <- net3$colors[net3$colors %in% c("brown", "yellow")]
clust.me <- net3$colors[net3$colors %in% c("black", "blue", "green", "pink", "red")]
clust.cn <- net3$colors[net3$colors %in% c("turquoise")]
clust.drop <- net3$colors[net3$colors %in% c("grey")]

ps.ph <- prune_taxa(names(clust.ph), ps.anc.f)
ps.me <- prune_taxa(names(clust.me), ps.anc.f)
ps.cn <- prune_taxa(names(clust.cn), ps.anc.f)
ps.drop <- prune_taxa(names(clust.drop), ps.anc.f)

tx.cn <- ps.cn %>% 
  tax_table() %>% 
  as.data.frame() %>% 
  add_column(Group = rep("Antarctic", length(taxa_names(ps.cn)))) %>% 
  add_column(abnd = taxa_sums(ps.rel)[taxa_names(ps.cn)])

tx.me <- ps.me %>% 
  tax_table() %>% 
  as.data.frame() %>% 
  add_column(Group = rep("Arctic", length(taxa_names(ps.me)))) %>% 
  add_column(abnd = taxa_sums(ps.rel)[taxa_names(ps.me)])

tx.ph <- ps.ph %>% 
  tax_table() %>% 
  as.data.frame() %>% 
  add_column(Group = rep("Caucasus", length(taxa_names(ps.ph)))) %>% 
  add_column(abnd = taxa_sums(ps.rel)[taxa_names(ps.ph)])

tx <- rbind(tx.cn, tx.me, tx.ph)

# physeq <- ps.anc.f
physeq <- ps.cr.f

veganifyOTU <- function(physeq){
  require(phyloseq)
  if(taxa_are_rows(physeq)){physeq <- t(physeq)}
  return(as(otu_table(physeq), "matrix"))
}

otus.ps.vegan <- veganifyOTU(physeq)
rownames(otus.ps.vegan) <- physeq@sam_data$Group
metadata <- as(sample_data(physeq), "data.frame")


cca_w_varstab_asv <- vegan::cca(otus.ps.vegan ~  pH + N.NH4 + TOC + P2O5 + Cu + Pb + Zn + Cd + Ni + N.NO3 + TOC + TON + CN + K2O, data=metadata)

tx.ids  <- tx %>% 
  rownames_to_column("ID") %>% 
  mutate(Score = "sites")

cca.meta <-  cca_w_varstab_asv$CCA$biplot %>% 
  as.data.frame() %>% 
  dplyr::select(c("CCA1", "CCA2")) %>% 
  rownames_to_column("ID") %>% 
  mutate(Score = "biplot")


list.pe <- summary(cca_w_varstab_asv)$cont$importance %>% 
  as.data.frame() %>% 
  dplyr::select(c("CCA1", "CCA2")) %>% 
  dplyr::slice(2L) %>% 
  t() %>% 
  as.vector()

fdat_amazing <- as.data.frame(cca_w_varstab_asv$CCA$v) %>%
  select(c("CCA1", "CCA2")) %>% 
  rownames_to_column("ID") %>% 
  right_join(tx.ids, by = join_by(ID)) %>% 
  mutate(label_size = 0) %>% 
  bind_rows(cca.meta) %>% 
  mutate(Group = as.factor(Group))

cca_clust <- ggplot(fdat_amazing) + 
  geom_point(data = fdat_amazing %>% dplyr::filter(Score == "sites"), 
             mapping = aes(x=CCA1, 
                           y=CCA2, 
                           color=Group, 
                           size=abnd), 
             alpha=0.5) + 
  geom_segment(data = fdat_amazing %>% dplyr::filter(Score == "biplot"), 
               aes(x = 0, 
                   xend = CCA1, 
                   y = 0, 
                   yend = CCA2), 
               alpha=0.8, 
               color = "red", 
               arrow = arrow(angle = 3)) +
  geom_text_repel(data = fdat_amazing %>% dplyr::filter(Score == "biplot"),
                  aes(x=CCA1, 
                      y=CCA2, 
                      label= ID), 
                  size=5) + 
  xlab(paste0(round(list.pe[1], 3)*100, "% CCA1")) +
  ylab(paste0(round(list.pe[2], 3)*100, "% CCA2")) +
  grids(linetype = "dashed") +
  geom_vline(xintercept = 0, size = 0.75, color = "#737373", alpha=0.5) +
  geom_hline(yintercept = 0, size = 0.75, color = "#737373", alpha=0.5) +
  theme(legend.position = "none", 
        panel.background = element_rect(fill = "white", colour = "grey50")) +
  scale_colour_viridis_d(option = "magma", 
                       aesthetics = "color", 
                       begin = 0.2, 
                       end = 0.8)  +
  labs(title = "B. ASVs - WGCNA clusters")

cca_clust
```

### 0.10.2 samples

```
# physeq <- ps.anc.f
physeq <- ps.cr.f
# physeq <- rarefy_even_depth(physeq, rngseed = 1312)

otus.ps.vegan <- veganifyOTU(physeq)
rownames(otus.ps.vegan) <- physeq@sam_data$Type
metadata <- as(sample_data(physeq), "data.frame") 

cca <- vegan::cca(otus.ps.vegan ~  pH + N.NH4 + TOC + P2O5 + Cu + Pb + Zn + Cd + Ni + N.NO3 + TOC + TON + CN + K2O, data=metadata)

biplot <- as.data.frame(cca$CCA$biplot)
wa <- as.data.frame(cca$CCA$wa)

biplot <- rownames_to_column(biplot, "Label") %>% 
  add_column(Score = rep("biplot", length(rownames(biplot)))) %>% 
  mutate(
  Group = rep("biplot", length(rownames(biplot))),
  Type = rep("biplot", length(rownames(biplot))),
  Region = rep("biplot", length(rownames(biplot)))
)

wa <- rownames_to_column(wa, "Label") %>% 
  add_column(Score = rep("sites", length(rownames(wa)))) %>% mutate(
  Type = metadata$Type,
  Group = metadata$Group,
  Region = metadata$Region
)

fdat_amazing <- rbind(biplot, wa) 

fdat_amazing <- fdat_amazing %>%
  mutate(label_size = ifelse(Score == "biplot", 4.5, 3)) %>% 
  mutate_if(is.character, as.factor)

list.pe <- summary(cca_w_varstab_asv)$cont$importance %>% 
  as.data.frame() %>% 
  select(c("CCA1", "CCA2")) %>% 
  dplyr::slice(2L) %>% 
  t() %>% 
  as.vector()

cca_sampl <- ggplot(fdat_amazing) + 
  geom_vline(xintercept = 0, size = 0.75, color = "#737373", alpha=0.5) +
  geom_hline(yintercept = 0, size = 0.75, color = "#737373", alpha=0.5) +
  geom_point(data = fdat_amazing %>% 
               dplyr::filter(Score == "sites"), mapping = aes(x=CCA1, y=CCA2, colour = factor(Score))) + 
  geom_segment(data = fdat_amazing %>% 
                 dplyr::filter(Score == "biplot"), 
               aes(x = 0, xend = CCA1, y = 0, yend = CCA2),
               size = 0.6, 
               alpha=0.8, 
               color = "red",
               arrow = arrow(angle = 3))  + 
  ggforce::geom_mark_ellipse(data = fdat_amazing %>% 
                                      dplyr::filter(Score == "sites"),
                             aes(x=CCA1, y=CCA2, group = Type, label = Type, col = 'grey'),
                  # label.fontsize = 12,
                  label.buffer = unit(2, "mm"),
                  label.minwidth = unit(5, "mm"),
                  con.cap = unit(0.1, "mm"),
                  con.colour='salmon',
                  label.colour='salmon') +
  ggrepel::geom_text_repel(data = fdat_amazing %>% 
                 dplyr::filter(Score == "biplot"), aes(x=CCA1, y=CCA2, label= Label), size=5) +
  xlab(paste0(round(list.pe[1], 3)*100, "% CCA1")) +
  ylab(paste0(round(list.pe[2], 3)*100, "% CCA2")) +
  grids(linetype = "dashed") +
  theme(legend.position = "none", 
        panel.background = element_rect(fill = "white", colour = "grey50")) +
  labs(title = "A. Sites")

 
cca_sampl
```

testing for terms

```
anova(cca, by='terms')
```

```
## Permutation test for cca under reduced model
## Terms added sequentially (first to last)
## Permutation: free
## Number of permutations: 999
## 
## Model: cca(formula = otus.ps.vegan ~ pH + N.NH4 + TOC + P2O5 + Cu + Pb + Zn + Cd + Ni + N.NO3 + TOC + TON + CN + K2O, data = metadata)
##          Df ChiSquare       F Pr(>F)    
## pH        1   0.67320 25.6680  0.001 ***
## N.NH4     1   0.64057 24.4236  0.001 ***
## TOC       1   0.24647  9.3974  0.001 ***
## P2O5      1   0.23159  8.8301  0.001 ***
## Cu        1   0.54239 20.6804  0.001 ***
## Pb        1   0.10756  4.1011  0.001 ***
## Zn        1   0.38958 14.8538  0.001 ***
## Cd        1   0.23059  8.7921  0.001 ***
## Ni        1   0.24839  9.4705  0.001 ***
## N.NO3     1   0.12215  4.6575  0.001 ***
## TON       1   0.12760  4.8653  0.001 ***
## CN        1   0.07524  2.8688  0.004 ** 
## K2O       1   0.10050  3.8320  0.001 ***
## Residual 54   1.41628                   
## ---
## Signif. codes:  0 '***' 0.001 '**' 0.01 '*' 0.05 '.' 0.1 ' ' 1
```

testing for margins

```
anova(cca, by='margin')
```

```
## Permutation test for cca under reduced model
## Marginal effects of terms
## Permutation: free
## Number of permutations: 999
## 
## Model: cca(formula = otus.ps.vegan ~ pH + N.NH4 + TOC + P2O5 + Cu + Pb + Zn + Cd + Ni + N.NO3 + TOC + TON + CN + K2O, data = metadata)
##          Df ChiSquare       F Pr(>F)    
## pH        1   0.07392  2.8184  0.002 ** 
## N.NH4     1   0.10188  3.8844  0.001 ***
## TOC       1   0.15590  5.9440  0.001 ***
## P2O5      1   0.28451 10.8478  0.001 ***
## Cu        1   0.13911  5.3038  0.001 ***
## Pb        1   0.08311  3.1688  0.002 ** 
## Zn        1   0.15514  5.9152  0.001 ***
## Cd        1   0.21783  8.3056  0.001 ***
## Ni        1   0.15306  5.8358  0.001 ***
## N.NO3     1   0.11948  4.5557  0.001 ***
## TON       1   0.14925  5.6905  0.001 ***
## CN        1   0.07153  2.7272  0.003 ** 
## K2O       1   0.10050  3.8320  0.001 ***
## Residual 54   1.41628                   
## ---
## Signif. codes:  0 '***' 0.001 '**' 0.01 '*' 0.05 '.' 0.1 ' ' 1
```

```
p.cca <- ggpubr::ggarrange(cca_sampl, cca_clust, legend = FALSE)

ggpubr::annotate_figure(p.cca, 
                        top = text_grob("cca - cryoconites", 
                                        color = "black", 
                                        face = "bold", 
                                        size = 12,
                                        hjust = 2.5),
               fig.lab.pos = "top.left")
```

### clusters

```
library(ggrepel)

tx.all <- ps.anc.f %>% 
  tax_table() %>% 
  as.data.frame() %>% 
  mutate(Group = net3$colors) %>% 
  add_column(abnd = taxa_sums(ps.rel)[taxa_names(ps.anc.f)]) 
# %>% 
#   filter(Group == "")

otus.ps.vegan <- veganifyOTU(physeq)
rownames(otus.ps.vegan) <- ps.anc.f@sam_data$Group
metadata <- as(sample_data(ps.anc.f), "data.frame") 

cca_w_varstab_asv <- vegan::cca(otus.ps.vegan ~  pH + N.NH4 + TOC + P2O5 + Cu + Pb + Zn + Cd + Ni + N.NO3 + TON + CN + K2O, data=metadata)

tx.ids  <- tx.all %>% 
  rownames_to_column("ID") %>% 
  mutate(Score = "sites")

cca.meta <-  cca_w_varstab_asv$CCA$biplot %>% 
  as.data.frame() %>% 
  select(c("CCA1", "CCA2")) %>% 
  rownames_to_column("ID") %>% 
  mutate(Score = "biplot")


fdat_amazing <- as.data.frame(cca_w_varstab_asv$CCA$v) %>%
  select(c("CCA1", "CCA2")) %>% 
  rownames_to_column("ID") %>% 
  right_join(tx.ids, by = join_by(ID)) %>% 
  mutate(label_size = 0) %>% 
  bind_rows(cca.meta)

list.pe <- summary(cca_w_varstab_asv)$cont$importance %>% 
  as.data.frame() %>% 
  select(c("CCA1", "CCA2")) %>% 
  dplyr::slice(2L) %>% 
  t() %>% 
  as.vector()

ggplot(fdat_amazing) + 
  geom_point(data = fdat_amazing %>% dplyr::filter(Score == "sites"), 
             mapping = aes(x=CCA1, 
                           y=CCA2, 
                           color=Group, 
                           size=abnd), 
             alpha=0.5) + 
  geom_segment(data = fdat_amazing %>% dplyr::filter(Score == "biplot"), 
               aes(x = 0, 
                   xend = CCA1, 
                   y = 0, 
                   yend = CCA2), 
               alpha=0.8, 
               color = "red", 
               arrow = arrow(angle = 3)) +
  geom_text_repel(data = fdat_amazing %>% dplyr::filter(Score == "biplot"),
                  aes(x=CCA1, 
                      y=CCA2, 
                      label= ID), 
                  size=6) + 
  xlab(paste0(round(list.pe[1], 3)*100, "% CCA1")) +
  ylab(paste0(round(list.pe[2], 3)*100, "% CCA2")) +
  grids(linetype = "dashed") +
  geom_vline(xintercept = 0, size = 0.75, color = "#737373", alpha=0.5) +
  geom_hline(yintercept = 0, size = 0.75, color = "#737373", alpha=0.5) +
  theme(legend.position = "top", 
        panel.background = element_rect(fill = "white", colour = "grey50")) +
  scale_colour_identity()
```

phylotypes(ASVs level) - without any specific filtration/normalisation at all  
only top 50 ASVs based on relative abundance

```
library(ggrepel)

physeq <- ps.cr
ps.varstab <- physeq

veganifyOTU <- function(physeq){
  require(phyloseq)
  if(taxa_are_rows(physeq)){physeq <- t(physeq)}
  return(as(otu_table(physeq), "matrix"))
}

otus.ps.vegan <- veganifyOTU(physeq)
metadata <- as(sample_data(physeq), "data.frame") 
cca_w_varstab_asv <- vegan::cca(otus.ps.vegan ~  pH + N.NH4 + TOC + P2O5 + Cu + Pb + Zn + Cd + Ni + N.NO3 + TON + CN + K2O, data=metadata)

wa <- as.data.frame(cca_w_varstab_asv$CCA$v) %>% 
  rownames_to_column("ID")

taxa.pruned <- as.data.frame(ps.varstab@tax_table@.Data) %>%
  rownames_to_column("ID")

taxa.pruned$taxa <- ifelse(is.na(taxa.pruned$Genus),
                            ifelse(is.na(taxa.pruned$Family),
                            ifelse(is.na(taxa.pruned$Order), 
                            ifelse(is.na(taxa.pruned$Class), taxa.pruned$Phylum, taxa.pruned$Class) , taxa.pruned$Order), taxa.pruned$Family), taxa.pruned$Genus)

taxa.pruned[taxa.pruned == "Burkholderia-Caballeronia-Paraburkholderia"] <- "Burkholderia"
taxa.pruned[taxa.pruned == "Allorhizobium-Neorhizobium-Pararhizobium-Rhizobium"] <- "Pararhizobium"

taxa.pruned$taxa2 <- ifelse(is.na(taxa.pruned$Species),
                            with(taxa.pruned, paste0(taxa)),
                            with(taxa.pruned, paste0(taxa, " ", Species )))
taxa.pruned$phylum <- ifelse(taxa.pruned$Phylum == "Proteobacteria", with(taxa.pruned, paste0(taxa.pruned$Class)), with(taxa.pruned, paste0(taxa.pruned$Phylum)))
# taxa.pruned$Label <- paste0(taxa.pruned$ID, "_", taxa.pruned$taxa2)
taxa.pruned$Label <- taxa.pruned$taxa2

wa <- full_join(wa, taxa.pruned, by="ID") %>% 
  mutate(Score = "sites")

#For the top 50 most abundant taxa, skip if use des res
sw <- summarize_all(as.data.frame(ps.varstab@otu_table), sum)
head_asv <- as_tibble(t(sw), rownames = "ID") %>% 
  arrange(desc(V1)) %>% top_n(n = 50) %>% 
  pull(ID)

wa <- filter(wa, ID %in% head_asv)

biplot <- as.data.frame(cca_w_varstab_asv$CCA$biplot)
biplot <- rownames_to_column(biplot, "Label") %>% 
  add_column(Score = rep("biplot", length(rownames(biplot))))

fdat_amazing <- plyr::rbind.fill(biplot, wa) %>% 
  mutate(Label = as.factor(Label))
fdat_amazing <- fdat_amazing %>%
  mutate(label_size = ifelse(Score == "biplot", 8, 5))

list.pe <- summary(cca_w_varstab_asv)$cont$importance %>% 
  as.data.frame() %>% 
  select(c("CCA2", "CCA2")) %>% 
  dplyr::slice(2L) %>% 
  t() %>% 
  as.vector()

p.cca.species <- ggplot(fdat_amazing %>% filter(Score %in% c("sites","biplot"))) + 
  geom_point(data = fdat_amazing %>% 
               dplyr::filter(Score == "sites"), mapping = aes(x=CCA1, y=CCA2)) + 
  geom_segment(data = fdat_amazing %>% 
                 dplyr::filter(Score == "biplot"), aes(x = 0, xend = CCA1, y = 0, yend = CCA2), alpha=0.8, color = "red", arrow = arrow(angle = 3)) +
  geom_text_repel(aes(x=CCA1, y=CCA2, label = Label, colour = phylum), 
                  force=3, 
                  force_pull=4,
                  max.iter = 100000,
                  max.time = 3,
                  max.overlaps = 20,
                  size=fdat_amazing$label_size) + 
  xlab(paste0(round(list.pe[1], 3)*100, "% CCA1")) +
  ylab(paste0(round(list.pe[2], 3)*100, "% CCA2")) +
  grids(linetype = "dashed") +
  geom_vline(xintercept = 0, size = 0.75, color = "#737373", alpha=0.5) +
  geom_hline(yintercept = 0, size = 0.75, color = "#737373", alpha=0.5) +
  theme(legend.position = "top", panel.background = element_rect(fill = "white", colour = "grey50"))

p.cca.species
```

```
clust.ph <- net3$colors[net3$colors %in% c("brown", "yellow")]
clust.green <- net3$colors[net3$colors %in% c("green")]

clust.cn <- net3$colors[net3$colors %in% c("turquoise")]
clust.drop <- net3$colors[net3$colors %in% c("grey")]

ps.ph <- prune_taxa(names(clust.ph), ps.anc.f)
ps.me <- prune_taxa(names(clust.me), ps.anc.f)
ps.cn <- prune_taxa(names(clust.cn), ps.anc.f)
ps.drop <- prune_taxa(names(clust.drop), ps.anc.f)

clust.green <- net3$colors[net3$colors %in% c("brown")]
ps.green <- prune_taxa(names(clust.green), ps.anc.f)
amp.green <- phyloseq_to_ampvis2(ps.green)
# amp_heatmap(amp.green,
#             tax_show = 30,
#             tax_aggregate = "OTU",
#             tax_add = "Genus",
#             normalise=FALSE, 
#             plot_values_size = 2, 
#             facet_by = "Region",
#             showRemainingTaxa = TRUE)

amp.gray <- phyloseq_to_ampvis2(ps.drop)

print('Brown cluster')
```

```
## [1] "Brown cluster"
```

```
amp_heatmap(amp.green,
            tax_show = 30,
            tax_aggregate = "OTU",
            tax_add = "Genus",
            group_by = "Type",
            normalise=FALSE, 
            plot_values_size = 2, 
            facet_by = "Region",
            showRemainingTaxa = TRUE)
```

```
# cca_w_varstab_asv$CA$v %>% rowSums() %>% as.data.frame() %>% 
  # arrange(desc(.))
```

## 0.11 treeplot

same, but in table format

```
tx %>% 
  rownames_to_column("ID") %>% 
  mutate(Phylum = case_when(Phylum %in% "Pseudomonadota" ~ Class,
                            !Phylum %in% "Pseudomonadota" ~ Phylum) %>% as.factor()) %>% 
  select(c("ID", "Genus", "Species", "Phylum", "Group", "abnd")) %>% 
  group_by(ID, Genus, Species, Phylum, Group ) %>%
  mutate(Genus = str_replace_na(Genus, replacement = ' ') %>% as.factor()) %>% 
  summarise(area = sum(abnd)) %>% 
  mutate_if(is.character, as.factor) %>% 
  drop_na()  %>%
  mutate(area = round(area, 2)) %>% 
  pivot_wider(names_from = Group, values_from = area)  %>% 
  DT::datatable(caption = "Groups based on WGCNA clusters")
```

Based on ANCOM-BC real abundance score

```
# merge_samples(ps.anc.f, "Region") %>% 
#   otu_table() %>%
#   data.frame()

cluster_map <- as.data.frame(net3$colors) %>% 
  mutate(big_clusters = with(., case_when(
    net3$colors %in% c("brown", "yellow") ~ 'PH',
    net3$colors %in% c("black", "blue", "green", "pink", "red") ~ 'ME',
    net3$colors %in% c("turquoise") ~ 'CN',
    net3$colors %in% c("grey") ~ 'NON'
  )))


ps.anc.f.clust <- ps.anc.f

ps.anc.f.clust@tax_table <- ps.anc.f@tax_table %>% 
  as.data.frame() %>% 
  cbind2(cluster_map) %>%
  as.matrix() %>% 
  tax_table()

ps.rel  <-  phyloseq::transform_sample_counts(ps.f2, function(x) x / sum(x) * 100)

tx <- ps.anc.f.clust %>% 
  tax_table() %>% 
  as.data.frame() %>% 
  add_column(abnd = taxa_sums(ps.anc.f.clust)) 


tx %>% 
  mutate(Phylum = case_when(Phylum %in% "Pseudomonadota" ~ Class,
                            !Phylum %in% "Pseudomonadota" ~ Phylum) %>% as.factor()) %>% 
  select(c("Genus", "Phylum", "big_clusters", "abnd")) %>% 
  group_by(Genus, Phylum, big_clusters ) %>%
  mutate(Genus = str_replace_na(Genus, replacement = ' ') %>% as.factor()) %>% 
  summarise(area = sum(abnd)) %>% 
  mutate_if(is.character, as.factor) %>% 
  drop_na() %>% 
  mutate(big_clusters=forcats::fct_relevel(big_clusters, c("CN" ,"ME","PH"))) %>% 
  filter(big_clusters != 'NON') %>% 
  ggplot(aes(area = area, 
             subgroup = Phylum, 
             label = Genus,
             fill = big_clusters)) +
  treemapify::geom_treemap() +
  treemapify::geom_treemap_subgroup_border(color = "white") +
  treemapify::geom_treemap_subgroup_text(place = "centre", 
                                         grow = T, 
                                         alpha = 0.7, 
                                         colour = "black", 
                                         fontface = "italic", 
                                         min.size = 2) +
  treemapify::geom_treemap_text(colour = "white", 
                                place = "topleft", 
                                grow = T, 
                                reflow = T, 
                                layout = 'squarified',
                                min.size = 4) +
  scale_colour_viridis_d(option = "magma", 
                         aesthetics = "fill", 
                         begin = 0.4, 
                         end = 0.8) +
  theme(legend.position="bottom")
```

Based on raw counts of reads - the prevalence of Cyanobacteria could be seen

```
# merge_samples(ps.anc.f, "Region") %>% 
#   otu_table() %>%
#   data.frame()

cluster_map <- as.data.frame(net3$colors) %>% 
  mutate(big_clusters = with(., case_when(
    net3$colors %in% c("brown", "yellow") ~ 'PH',
    net3$colors %in% c("black", "blue", "green", "pink", "red") ~ 'ME',
    net3$colors %in% c("turquoise") ~ 'CN',
    net3$colors %in% c("grey") ~ 'NON'
  )))


ps.anc.f.clust <- ps.anc.f

ps.anc.f.clust@tax_table <- ps.anc.f@tax_table %>% 
  as.data.frame() %>% 
  cbind2(cluster_map) %>%
  as.matrix() %>% 
  tax_table()


tx <- ps.anc.f.clust %>% 
  tax_table() %>% 
  as.data.frame() %>% 
  add_column(abnd = taxa_sums(ps.f2)[taxa_names(ps.anc.f.clust)]) 


tx %>% 
  mutate(Phylum = case_when(Phylum %in% "Pseudomonadota" ~ Class,
                            !Phylum %in% "Pseudomonadota" ~ Phylum) %>% as.factor()) %>% 
  select(c("Genus", "Phylum", "big_clusters", "abnd")) %>% 
  group_by(Genus, Phylum, big_clusters ) %>%
  mutate(Genus = str_replace_na(Genus, replacement = ' ') %>% as.factor()) %>% 
  summarise(area = sum(abnd)) %>% 
  mutate_if(is.character, as.factor) %>% 
  drop_na() %>% 
  mutate(big_clusters=forcats::fct_relevel(big_clusters, c("CN" ,"ME","PH"))) %>% 
  filter(big_clusters != 'NON') %>% 
  ggplot(aes(area = area, 
             subgroup = Phylum, 
             label = Genus,
             fill = big_clusters)) +
  treemapify::geom_treemap() +
  treemapify::geom_treemap_subgroup_border(color = "white") +
  treemapify::geom_treemap_subgroup_text(place = "centre", 
                                         grow = T, 
                                         alpha = 0.7, 
                                         colour = "black", 
                                         fontface = "italic", 
                                         min.size = 2) +
  treemapify::geom_treemap_text(colour = "white", 
                                place = "topleft", 
                                grow = T, 
                                reflow = T, 
                                layout = 'squarified',
                                min.size = 4) +
  scale_colour_viridis_d(option = "magma", 
                         aesthetics = "fill", 
                         begin = 0.4, 
                         end = 0.8) +
  theme(legend.position="bottom")
```

### 0.11.1 Data export - rel abundance for clusters - type and region

```
ps.f2.regm <- ps.f2 %>%
  merge_samples('Region')

ps.f2.regm.rel <- phyloseq::transform_sample_counts(ps.f2.regm, function(x) x / sum(x) * 100)
ps.f2.regm.rel.clust <- prune_taxa(taxa_names(ps.anc.f.clust), ps.f2.regm.rel)

# ps.anc.f.clust@tax_table <- ps.anc.f@tax_table %>% 
#   as.data.frame() %>% 
#   cbind2(cluster_map) %>%
#   as.matrix() %>% 
#   tax_table()
  
# ps.f2.regm.rel.clust %>% 
#   phyloseq::psmelt()

# region_rel_abund <-
  
  
region_rel_abund <- ps.f2.regm.rel.clust %>% 
  phyloseq::psmelt() %>% 
  dplyr::select(c('OTU','Abundance', 'Sample')) %>% 
  pivot_wider(names_from = Sample, values_from = Abundance) %>% 
  column_to_rownames('OTU')


ps.f2.tpm <- ps.f2 %>%
  merge_samples('Type')

ps.f2.tpm.rel <- phyloseq::transform_sample_counts(ps.f2.tpm, function(x) x / sum(x) * 100)
ps.f2.tpm.rel.clust <- prune_taxa(taxa_names(ps.anc.f.clust), ps.f2.tpm.rel)

type_rel_abund <- ps.f2.tpm.rel.clust %>% 
  phyloseq::psmelt() %>% 
  dplyr::select(c('OTU','Abundance',  'Sample')) %>% 
  pivot_wider(names_from = Sample, values_from = Abundance) %>% 
  column_to_rownames('OTU')

rel_out <- tx %>% 
  cbind2(region_rel_abund) %>% 
  cbind2(type_rel_abund) %>% 
  dplyr::select(-abnd)

rel_out %>% 
  DT::datatable(caption = "Groups based on WGCNA clusters")
```

```
# par <- parrottx %>% 
#   select(c('big_clusters', 'arctic', 'antarctic', 'caucasus')) %>% 
#   pivot_longer(-big_clusters, names_to = 'Region', values_to = 'Abnd')  %>% 
#   mutate_if(is.character, as.factor) %>% 
#   group_by(big_clusters, Region) %>% 
#   summarise_all(sum)
# 
# par
# 
# ggplot(data = par, aes(x = "", y = Abnd, fill = Region )) + 
#     geom_bar(stat = "identity", position = position_fill()) +
#     geom_text(aes(label = Region), position = position_fill(vjust = 0.5)) +
#     coord_polar(theta = "y") +
#     facet_wrap(~ big_clusters)  +
#     theme(axis.title.x = element_blank(),
#           axis.title.y = element_blank()) + 
#     theme(legend.position='bottom') + 
#     guides(fill=guide_legend(nrow=2, byrow=TRUE))
```

## 0.12 Permanova

By Type - 700 ASVs ANCOMBC sample fraction normalisation

```
dist <- phyloseq::distance(ps.anc.f, "euclidean")
metadata <- as(sample_data(ps.anc.f@sam_data), "data.frame")
vegan::adonis2(dist ~ Type, data = metadata)
```

```
## Permutation test for adonis under reduced model
## Terms added sequentially (first to last)
## Permutation: free
## Number of permutations: 999
## 
## vegan::adonis2(formula = dist ~ Type, data = metadata)
##          Df SumOfSqs      R2      F Pr(>F)    
## Type      5    93528 0.78874 46.296  0.001 ***
## Residual 62    25051 0.21126                  
## Total    67   118578 1.00000                  
## ---
## Signif. codes:  0 '***' 0.001 '**' 0.01 '*' 0.05 '.' 0.1 ' ' 1
```

By Type - rarefyed full data

```
dist <- phyloseq::distance(ps.f2.r, "euclidean")
metadata <- as(sample_data(ps.f2.r@sam_data), "data.frame")
vegan::adonis2(dist ~ Type, data = metadata)
```

```
## Permutation test for adonis under reduced model
## Terms added sequentially (first to last)
## Permutation: free
## Number of permutations: 999
## 
## vegan::adonis2(formula = dist ~ Type, data = metadata)
##          Df  SumOfSqs      R2      F Pr(>F)    
## Type      5 110303903 0.65186 23.218  0.001 ***
## Residual 62  58909004 0.34814                  
## Total    67 169212907 1.00000                  
## ---
## Signif. codes:  0 '***' 0.001 '**' 0.01 '*' 0.05 '.' 0.1 ' ' 1
```

By Type - clr full data

```
dist <- phyloseq::distance(ps.f2.clr, "euclidean")
metadata <- as(sample_data(ps.f2.clr@sam_data), "data.frame")
vegan::adonis2(dist ~ Type, data = metadata)
```

```
## Permutation test for adonis under reduced model
## Terms added sequentially (first to last)
## Permutation: free
## Number of permutations: 999
## 
## vegan::adonis2(formula = dist ~ Type, data = metadata)
##          Df SumOfSqs      R2      F Pr(>F)    
## Type      5    12701 0.32818 6.0572  0.001 ***
## Residual 62    26001 0.67182                  
## Total    67    38703 1.00000                  
## ---
## Signif. codes:  0 '***' 0.001 '**' 0.01 '*' 0.05 '.' 0.1 ' ' 1
```

By Region - 700 ASVs ANCOMBC sample fraction normalisation

```
dist <- phyloseq::distance(ps.anc.f, "euclidean")
metadata <- as(sample_data(ps.anc.f@sam_data), "data.frame")
vegan::adonis2(dist ~ Region, data = metadata)
```

```
## Permutation test for adonis under reduced model
## Terms added sequentially (first to last)
## Permutation: free
## Number of permutations: 999
## 
## vegan::adonis2(formula = dist ~ Region, data = metadata)
##          Df SumOfSqs      R2      F Pr(>F)    
## Region    2    61829 0.52142 35.409  0.001 ***
## Residual 65    56749 0.47858                  
## Total    67   118578 1.00000                  
## ---
## Signif. codes:  0 '***' 0.001 '**' 0.01 '*' 0.05 '.' 0.1 ' ' 1
```

By Region - clr full data

```
dist <- phyloseq::distance(ps.f2.clr, "euclidean")
metadata <- as(sample_data(ps.f2.clr@sam_data), "data.frame")
vegan::adonis2(dist ~ Region, data = metadata)
```

```
## Permutation test for adonis under reduced model
## Terms added sequentially (first to last)
## Permutation: free
## Number of permutations: 999
## 
## vegan::adonis2(formula = dist ~ Region, data = metadata)
##          Df SumOfSqs      R2      F Pr(>F)    
## Region    2     6481 0.16746 6.5372  0.001 ***
## Residual 65    32221 0.83254                  
## Total    67    38703 1.00000                  
## ---
## Signif. codes:  0 '***' 0.001 '**' 0.01 '*' 0.05 '.' 0.1 ' ' 1
```

By full chemestry - 700 ASVs ANCOMBC sample fraction normalisation

```
dist <- phyloseq::distance(ps.anc.f, "euclidean")
metadata <- as(sample_data(ps.anc.f@sam_data), "data.frame")
vegan::adonis2(dist ~ pH + N.NH4 + TOC + P2O5 + Pb + Zn + Cd + Ni + N.NO3 + TOC, data = metadata)
```

```
## Permutation test for adonis under reduced model
## Terms added sequentially (first to last)
## Permutation: free
## Number of permutations: 999
## 
## vegan::adonis2(formula = dist ~ pH + N.NH4 + TOC + P2O5 + Pb + Zn + Cd + Ni + N.NO3 + TOC, data = metadata)
##          Df SumOfSqs      R2       F Pr(>F)    
## pH        1    20125 0.16972 46.2966  0.001 ***
## N.NH4     1    22046 0.18592 50.7170  0.001 ***
## TOC       1     4260 0.03593  9.8004  0.001 ***
## P2O5      1     6056 0.05107 13.9316  0.001 ***
## Pb        1    11794 0.09946 27.1326  0.001 ***
## Zn        1     7394 0.06235 17.0086  0.001 ***
## Cd        1     8806 0.07427 20.2585  0.001 ***
## Ni        1    11726 0.09889 26.9764  0.001 ***
## N.NO3     1     1158 0.00977  2.6642  0.022 *  
## Residual 58    25212 0.21262                   
## Total    67   118578 1.00000                   
## ---
## Signif. codes:  0 '***' 0.001 '**' 0.01 '*' 0.05 '.' 0.1 ' ' 1
```

By full chemestry - clr full data

```
dist <- phyloseq::distance(ps.f2.clr, "euclidean")
metadata <- as(sample_data(ps.f2.clr@sam_data), "data.frame")
vegan::adonis2(dist ~ pH + N.NH4 + TOC + P2O5 + Pb + Zn + Cd + Ni + N.NO3 + TOC, data = metadata)
```

```
## Permutation test for adonis under reduced model
## Terms added sequentially (first to last)
## Permutation: free
## Number of permutations: 999
## 
## vegan::adonis2(formula = dist ~ pH + N.NH4 + TOC + P2O5 + Pb + Zn + Cd + Ni + N.NO3 + TOC, data = metadata)
##          Df SumOfSqs      R2      F Pr(>F)    
## pH        1     2475 0.06396 6.0374  0.001 ***
## N.NH4     1     2874 0.07427 7.0103  0.001 ***
## TOC       1     1209 0.03125 2.9498  0.001 ***
## P2O5      1     1296 0.03350 3.1620  0.001 ***
## Pb        1     1753 0.04530 4.2759  0.001 ***
## Zn        1     1500 0.03876 3.6591  0.001 ***
## Cd        1     1245 0.03216 3.0360  0.001 ***
## Ni        1     1911 0.04937 4.6606  0.001 ***
## N.NO3     1      658 0.01700 1.6047  0.021 *  
## Residual 58    23780 0.61443                  
## Total    67    38703 1.00000                  
## ---
## Signif. codes:  0 '***' 0.001 '**' 0.01 '*' 0.05 '.' 0.1 ' ' 1
```

Reduced chemestry

```
dist <- phyloseq::distance(ps.anc.f, "euclidean")
metadata <- as(sample_data(ps.anc.f@sam_data), "data.frame")
vegan::adonis2(dist ~ pH + N.NH4 + Cd , data = metadata)
```

```
## Permutation test for adonis under reduced model
## Terms added sequentially (first to last)
## Permutation: free
## Number of permutations: 999
## 
## vegan::adonis2(formula = dist ~ pH + N.NH4 + Cd, data = metadata)
##          Df SumOfSqs      R2      F Pr(>F)    
## pH        1    20125 0.16972 22.312  0.001 ***
## N.NH4     1    22046 0.18592 24.442  0.001 ***
## Cd        1    18681 0.15754 20.711  0.001 ***
## Residual 64    57726 0.48682                  
## Total    67   118578 1.00000                  
## ---
## Signif. codes:  0 '***' 0.001 '**' 0.01 '*' 0.05 '.' 0.1 ' ' 1
```

### 0.12.1 Add Type

```
dist <- phyloseq::distance(ps.f2.clr, "euclidean")
metadata <- as(sample_data(ps.f2.clr@sam_data), "data.frame")
vegan::adonis2(dist ~ Type + pH + N.NH4 + TOC + P2O5 + Pb + Zn + Cd + Ni + N.NO3 + TOC, data = metadata)
```

```
## Permutation test for adonis under reduced model
## Terms added sequentially (first to last)
## Permutation: free
## Number of permutations: 999
## 
## vegan::adonis2(formula = dist ~ Type + pH + N.NH4 + TOC + P2O5 + Pb + Zn + Cd + Ni + N.NO3 + TOC, data = metadata)
##          Df SumOfSqs      R2      F Pr(>F)    
## Type      5    12701 0.32818 6.9398  0.001 ***
## pH        1      484 0.01250 1.3217  0.104    
## N.NH4     1      988 0.02553 2.6990  0.001 ***
## TOC       1      802 0.02073 2.1914  0.001 ***
## P2O5      1      974 0.02516 2.6601  0.001 ***
## Pb        1      416 0.01076 1.1378  0.230    
## Zn        1     1136 0.02936 3.1042  0.001 ***
## Cd        1      815 0.02105 2.2259  0.001 ***
## Ni        1      537 0.01387 1.4669  0.027 *  
## N.NO3     1      449 0.01160 1.2267  0.153    
## Residual 53    19400 0.50126                  
## Total    67    38703 1.00000                  
## ---
## Signif. codes:  0 '***' 0.001 '**' 0.01 '*' 0.05 '.' 0.1 ' ' 1
```

```
dist <- phyloseq::distance(ps.f2.clr, "euclidean")
metadata <- as(sample_data(ps.f2.clr@sam_data), "data.frame")
vegan::adonis2(dist ~ pH + N.NH4 + TOC + P2O5 + Pb + Zn + Cd + Ni + N.NO3 + TOC + Type, data = metadata)
```

```
## Permutation test for adonis under reduced model
## Terms added sequentially (first to last)
## Permutation: free
## Number of permutations: 999
## 
## vegan::adonis2(formula = dist ~ pH + N.NH4 + TOC + P2O5 + Pb + Zn + Cd + Ni + N.NO3 + TOC + Type, data = metadata)
##          Df SumOfSqs      R2      F Pr(>F)    
## pH        1     2475 0.06396 6.7625  0.001 ***
## N.NH4     1     2874 0.07427 7.8522  0.001 ***
## TOC       1     1209 0.03125 3.3041  0.001 ***
## P2O5      1     1296 0.03350 3.5418  0.001 ***
## Pb        1     1753 0.04530 4.7894  0.001 ***
## Zn        1     1500 0.03876 4.0986  0.001 ***
## Cd        1     1245 0.03216 3.4006  0.001 ***
## Ni        1     1911 0.04937 5.2203  0.001 ***
## N.NO3     1      658 0.01700 1.7975  0.003 ** 
## Type      5     4380 0.11317 2.3931  0.001 ***
## Residual 53    19400 0.50126                  
## Total    67    38703 1.00000                  
## ---
## Signif. codes:  0 '***' 0.001 '**' 0.01 '*' 0.05 '.' 0.1 ' ' 1
```

## 0.13 Packages info

```
sessionInfo()
```

```
## R version 4.3.2 (2023-10-31)
## Platform: x86_64-pc-linux-gnu (64-bit)
## Running under: Ubuntu 20.04.6 LTS
## 
## Matrix products: default
## BLAS:   /usr/lib/x86_64-linux-gnu/blas/libblas.so.3.9.0 
## LAPACK: /usr/lib/x86_64-linux-gnu/lapack/liblapack.so.3.9.0
## 
## locale:
##  [1] LC_CTYPE=en_US.UTF-8       LC_NUMERIC=C              
##  [3] LC_TIME=en_US.UTF-8        LC_COLLATE=en_US.UTF-8    
##  [5] LC_MONETARY=en_US.UTF-8    LC_MESSAGES=en_US.UTF-8   
##  [7] LC_PAPER=en_US.UTF-8       LC_NAME=C                 
##  [9] LC_ADDRESS=C               LC_TELEPHONE=C            
## [11] LC_MEASUREMENT=en_US.UTF-8 LC_IDENTIFICATION=C       
## 
## time zone: Europe/Moscow
## tzcode source: system (glibc)
## 
## attached base packages:
## [1] stats     graphics  grDevices utils     datasets  methods   base     
## 
## other attached packages:
##  [1] ggrepel_0.9.5         KneeArrower_1.0.0     ggtree_3.8.2         
##  [4] heatmaply_1.5.0       viridis_0.6.5         viridisLite_0.4.2    
##  [7] plotly_4.10.4         ampvis2_2.7.28        ggpubr_0.6.0         
## [10] lubridate_1.9.3       forcats_1.0.0         stringr_1.5.1        
## [13] dplyr_1.1.4           purrr_1.0.2           readr_2.1.5          
## [16] tidyr_1.3.1           tibble_3.2.1          ggplot2_3.5.0        
## [19] tidyverse_2.0.0       phyloseq_1.44.0       WGCNA_1.72-5         
## [22] fastcluster_1.2.6     dynamicTreeCut_1.63-1
## 
## loaded via a namespace (and not attached):
##   [1] IRanges_2.34.1                 gld_2.6.6                     
##   [3] nnet_7.3-19                    ggfittext_0.10.2              
##   [5] DT_0.32                        Biostrings_2.68.1             
##   [7] TH.data_1.1-2                  vctrs_0.6.5                   
##   [9] energy_1.7-11                  digest_0.6.35                 
##  [11] png_0.1-8                      proxy_0.4-27                  
##  [13] Exact_3.2                      registry_0.5-1                
##  [15] permute_0.9-7                  MASS_7.3-60.0.1               
##  [17] reshape2_1.4.4                 foreach_1.5.2                 
##  [19] BiocGenerics_0.46.0            withr_3.0.0                   
##  [21] xfun_0.42                      ggfun_0.1.4                   
##  [23] ellipsis_0.3.2                 survival_3.5-8                
##  [25] doRNG_1.8.6                    memoise_2.0.1                 
##  [27] ggbeeswarm_0.7.2               gmp_0.7-4                     
##  [29] systemfonts_1.0.6              tidytree_0.4.6                
##  [31] zoo_1.8-12                     gtools_3.9.5                  
##  [33] DEoptimR_1.1-3                 Formula_1.2-5                 
##  [35] KEGGREST_1.40.1                httr_1.4.7                    
##  [37] rstatix_0.7.2                  rhdf5filters_1.12.1           
##  [39] rhdf5_2.44.0                   rstudioapi_0.15.0             
##  [41] ggVennDiagram_1.5.2            generics_0.1.3                
##  [43] base64enc_0.1-3                S4Vectors_0.38.2              
##  [45] zlibbioc_1.46.0                ScaledMatrix_1.8.1            
##  [47] polyclip_1.10-6                ca_0.71.1                     
##  [49] GenomeInfoDbData_1.2.10        ade4_1.7-22                   
##  [51] doParallel_1.0.17              evaluate_0.23                 
##  [53] S4Arrays_1.0.6                 preprocessCore_1.62.1         
##  [55] hms_1.1.3                      GenomicRanges_1.52.1          
##  [57] irlba_2.3.5.1                  colorspace_2.1-0              
##  [59] readxl_1.4.3                   magrittr_2.0.3                
##  [61] lattice_0.22-5                 robustbase_0.99-2             
##  [63] DECIPHER_2.28.0                scuttle_1.10.3                
##  [65] cowplot_1.1.3                  matrixStats_1.2.0             
##  [67] class_7.3-22                   Hmisc_5.1-2                   
##  [69] pillar_1.9.0                   nlme_3.1-164                  
##  [71] iterators_1.0.14               decontam_1.20.0               
##  [73] compiler_4.3.2                 beachmat_2.16.0               
##  [75] stringi_1.8.3                  biomformat_1.28.0             
##  [77] treemapify_2.5.6               DescTools_0.99.54             
##  [79] TSP_1.2-4                      minqa_1.2.6                   
##  [81] SummarizedExperiment_1.30.2    dendextend_1.17.1             
##  [83] plyr_1.8.9                     crayon_1.5.2                  
##  [85] abind_1.4-5                    scater_1.28.0                 
##  [87] gridGraphics_0.5-1             bit_4.0.5                     
##  [89] mia_1.8.0                      rootSolve_1.8.2.4             
##  [91] sandwich_3.1-0                 codetools_0.2-19              
##  [93] multcomp_1.4-25                BiocSingular_1.16.0           
##  [95] crosstalk_1.2.1                bslib_0.6.1                   
##  [97] e1071_1.7-14                   lmom_3.0                      
##  [99] multtest_2.56.0                MultiAssayExperiment_1.26.0   
## [101] splines_4.3.2                  Rcpp_1.0.12                   
## [103] sparseMatrixStats_1.12.2       cellranger_1.1.0              
## [105] knitr_1.45                     egg_0.4.5                     
## [107] blob_1.2.4                     utf8_1.2.4                    
## [109] lme4_1.1-35.1                  fs_1.6.3                      
## [111] checkmate_2.3.1                DelayedMatrixStats_1.22.6     
## [113] Rdpack_2.6                     expm_0.999-9                  
## [115] gsl_2.1-8                      ggsignif_0.6.4                
## [117] ggplotify_0.1.2                Matrix_1.6-5                  
## [119] tzdb_0.4.0                     tweenr_2.0.3                  
## [121] pkgconfig_2.0.3                tools_4.3.2                   
## [123] cachem_1.0.8                   rbibutils_2.2.16              
## [125] RSQLite_2.3.5                  DBI_1.2.2                     
## [127] numDeriv_2016.8-1.1            signal_1.8-0                  
## [129] impute_1.74.1                  fastmap_1.1.1                 
## [131] rmarkdown_2.26                 scales_1.3.0                  
## [133] grid_4.3.2                     broom_1.0.5                   
## [135] sass_0.4.8                     patchwork_1.2.0               
## [137] carData_3.0-5                  rpart_4.1.23                  
## [139] farver_2.1.1                   mgcv_1.9-1                    
## [141] yaml_2.3.8                     MatrixGenerics_1.12.3         
## [143] foreign_0.8-86                 bayesm_3.1-6                  
## [145] cli_3.6.2                      stats4_4.3.2                  
## [147] webshot_0.5.5                  lifecycle_1.0.4               
## [149] Biobase_2.60.0                 mvtnorm_1.2-4                 
## [151] backports_1.4.1                BiocParallel_1.34.2           
## [153] timechange_0.3.0               gtable_0.3.4                  
## [155] ANCOMBC_2.2.2                  parallel_4.3.2                
## [157] ape_5.7-1                      CVXR_1.0-12                   
## [159] jsonlite_1.8.8                 seriation_1.5.4               
## [161] bitops_1.0-7                   bit64_4.0.5                   
## [163] assertthat_0.2.1               yulab.utils_0.1.4             
## [165] vegan_2.6-4                    BiocNeighbors_1.18.0          
## [167] TreeSummarizedExperiment_2.8.0 jquerylib_0.1.4               
## [169] highr_0.10                     lazyeval_0.2.2                
## [171] htmltools_0.5.7                GO.db_3.17.0                  
## [173] glue_1.7.0                     XVector_0.40.0                
## [175] RCurl_1.98-1.14                treeio_1.24.3                 
## [177] gridExtra_2.3                  boot_1.3-30                   
## [179] igraph_2.0.2                   R6_2.5.1                      
## [181] SingleCellExperiment_1.22.0    Rmpfr_0.9-5                   
## [183] labeling_0.4.3                 cluster_2.1.6                 
## [185] rngtools_1.5.2                 Rhdf5lib_1.22.1               
## [187] aplot_0.2.2                    GenomeInfoDb_1.36.4           
## [189] nloptr_2.0.3                   compositions_2.0-8            
## [191] DirichletMultinomial_1.42.0    DelayedArray_0.26.7           
## [193] tidyselect_1.2.1               vipor_0.4.7                   
## [195] htmlTable_2.4.2                tensorA_0.36.2.1              
## [197] ggforce_0.4.2                  car_3.1-2                     
## [199] AnnotationDbi_1.62.2           rsvd_1.0.5                    
## [201] munsell_0.5.0                  data.table_1.15.2             
## [203] htmlwidgets_1.6.4              RColorBrewer_1.1-3            
## [205] rlang_1.1.3                    lmerTest_3.1-3                
## [207] fansi_1.0.6                    beeswarm_0.4.0
```

## 0.14 Second round

```
beta_custom_norm_NMDS_elli_w(ps.anc.f, Color = "Group", Group = "Group")
```

```
## Run 0 stress 0.06790316 
## Run 1 stress 0.06790309 
## ... New best solution
## ... Procrustes: rmse 0.0001297073  max resid 0.0002323765 
## ... Similar to previous best
## Run 2 stress 0.0679002 
## ... New best solution
## ... Procrustes: rmse 0.0006697249  max resid 0.003179864 
## ... Similar to previous best
## Run 3 stress 0.06790358 
## ... Procrustes: rmse 0.0007609433  max resid 0.003177331 
## ... Similar to previous best
## Run 4 stress 0.06789996 
## ... New best solution
## ... Procrustes: rmse 0.0002999861  max resid 0.0005373441 
## ... Similar to previous best
## Run 5 stress 0.06804586 
## ... Procrustes: rmse 0.003281042  max resid 0.0211396 
## Run 6 stress 0.06790346 
## ... Procrustes: rmse 0.0006402587  max resid 0.003289149 
## ... Similar to previous best
## Run 7 stress 0.06799468 
## ... Procrustes: rmse 0.004732599  max resid 0.02151816 
## Run 8 stress 0.06789998 
## ... Procrustes: rmse 0.000008025259  max resid 0.00002206461 
## ... Similar to previous best
## Run 9 stress 0.0724489 
## Run 10 stress 0.06790018 
## ... Procrustes: rmse 0.0002956927  max resid 0.0005293566 
## ... Similar to previous best
## Run 11 stress 0.0680118 
## ... Procrustes: rmse 0.002901032  max resid 0.02094542 
## Run 12 stress 0.06789995 
## ... New best solution
## ... Procrustes: rmse 0.000005735243  max resid 0.00001690243 
## ... Similar to previous best
## Run 13 stress 0.06789992 
## ... New best solution
## ... Procrustes: rmse 0.0001842  max resid 0.0003338535 
## ... Similar to previous best
## Run 14 stress 0.06803875 
## ... Procrustes: rmse 0.003728348  max resid 0.02094877 
## Run 15 stress 0.07193131 
## Run 16 stress 0.0679002 
## ... Procrustes: rmse 0.0002637468  max resid 0.0004768878 
## ... Similar to previous best
## Run 17 stress 0.06789995 
## ... Procrustes: rmse 0.0001793291  max resid 0.0003253821 
## ... Similar to previous best
## Run 18 stress 0.07225657 
## Run 19 stress 0.06801192 
## ... Procrustes: rmse 0.003005419  max resid 0.02073154 
## Run 20 stress 0.06799469 
## ... Procrustes: rmse 0.004739737  max resid 0.02128688 
## *** Best solution repeated 3 times
```

```
physeq <- ps.anc.f
# physeq <- ps.cr.f
# physeq <- rarefy_even_depth(physeq, rngseed = 1312)

otus.ps.vegan <- veganifyOTU(physeq)
rownames(otus.ps.vegan) <- physeq@sam_data$Type
metadata <- as(sample_data(physeq), "data.frame") 

rda <- vegan::rda(otus.ps.vegan ~  pH + N.NH4 + TOC + P2O5 + Cu + Pb + Zn + Cd + Ni + N.NO3 + TOC + TON + CN + K2O, data=metadata)

biplot <- as.data.frame(rda$CCA$biplot)
wa <- as.data.frame(rda$CCA$wa)

biplot <- rownames_to_column(biplot, "Label") %>% 
  add_column(Score = rep("biplot", length(rownames(biplot)))) %>% 
  mutate(
  Group = rep("biplot", length(rownames(biplot))),
  Type = rep("biplot", length(rownames(biplot))),
  Region = rep("biplot", length(rownames(biplot)))
)

wa <- rownames_to_column(wa, "Label") %>% 
  add_column(Score = rep("sites", length(rownames(wa)))) %>% mutate(
  Type = metadata$Type,
  Group = metadata$Group,
  Region = metadata$Region
)

fdat_amazing <- rbind(biplot, wa) 

fdat_amazing <- fdat_amazing %>%
  mutate(label_size = ifelse(Score == "biplot", 4.5, 3)) %>% 
  mutate_if(is.character, as.factor)

list.pe <- summary(rda)$cont$importance %>% 
  as.data.frame() %>% 
  select(c("RDA1", "RDA2")) %>% 
  dplyr::slice(2L) %>% 
  t() %>% 
  as.vector()

rda_sampl <- ggplot(fdat_amazing) + 
  geom_vline(xintercept = 0, size = 0.75, color = "#737373", alpha=0.5) +
  geom_hline(yintercept = 0, size = 0.75, color = "#737373", alpha=0.5) +
  geom_point(data = fdat_amazing %>% 
               dplyr::filter(Score == "sites"), mapping = aes(x=RDA1, y=RDA2, colour = factor(Score))) + 
  geom_segment(data = fdat_amazing %>% 
                 dplyr::filter(Score == "biplot"), 
               aes(x = 0, xend = RDA1, y = 0, yend = RDA2),
               size = 0.6, 
               alpha=0.8, 
               color = "red",
               arrow = arrow(angle = 3))  + 
  ggforce::geom_mark_ellipse(data = fdat_amazing %>% 
                                      dplyr::filter(Score == "sites"),
                             aes(x=RDA1, y=RDA2, group = Type, label = Type, col = 'grey'),
                  # label.fontsize = 12,
                  label.buffer = unit(2, "mm"),
                  label.minwidth = unit(5, "mm"),
                  con.cap = unit(0.1, "mm"),
                  con.colour='salmon',
                  label.colour='salmon') +
  ggrepel::geom_text_repel(data = fdat_amazing %>% 
                 dplyr::filter(Score == "biplot"), aes(x=RDA1, y=RDA2, label= Label), size=5) +
  xlab(paste0(round(list.pe[1], 3)*100, "% RDA1")) +
  ylab(paste0(round(list.pe[2], 3)*100, "% RDA2")) +
  grids(linetype = "dashed") +
  theme(legend.position = "none", 
        panel.background = element_rect(fill = "white", colour = "grey50")) +
  labs(title = "A. Sites")

 
rda_sampl
```

```
cca$
biplot
```

```
## NULL
```

```
ps.cr.f
```

```
## phyloseq-class experiment-level object
## otu_table()   OTU Table:         [ 716 taxa and 68 samples ]
## sample_data() Sample Data:       [ 68 samples by 21 sample variables ]
## tax_table()   Taxonomy Table:    [ 716 taxa by 7 taxonomic ranks ]
```

```
physeq <- ps.cr.f
# physeq <- ps.cr.f
physeq <- rarefy_even_depth(physeq, rngseed = 1312)

otus.ps.vegan <- veganifyOTU(physeq)
rownames(otus.ps.vegan) <- physeq@sam_data$Type
metadata <- as(sample_data(physeq), "data.frame") 

cca <- vegan::cca(otus.ps.vegan ~ pH + N.NH4 + TOC + P2O5 + Cu + Pb + Zn + Cd + Ni + N.NO3 + TOC + TON + CN + K2O, data=metadata)

biplot <- as.data.frame(cca$CCA$biplot)
wa <- as.data.frame(cca$CCA$wa)

biplot <- rownames_to_column(biplot, "Label") %>% 
  add_column(Score = rep("biplot", length(rownames(biplot)))) %>% 
  mutate(
  Group = rep("biplot", length(rownames(biplot))),
  Type = rep("biplot", length(rownames(biplot))),
  Region = rep("biplot", length(rownames(biplot)))
)

wa <- rownames_to_column(wa, "Label") %>% 
  add_column(Score = rep("sites", length(rownames(wa)))) %>% mutate(
  Type = metadata$Type,
  Group = metadata$Group,
  Region = metadata$Region
)

fdat_amazing <- rbind(biplot, wa) 

fdat_amazing <- fdat_amazing %>%
  mutate(label_size = ifelse(Score == "biplot", 4.5, 3)) %>% 
  mutate_if(is.character, as.factor)

list.pe <- summary(cca)$cont$importance %>% 
  as.data.frame() %>% 
  select(c("CCA1", "CCA2")) %>% 
  dplyr::slice(2L) %>% 
  t() %>% 
  as.vector()

cca_sampl <- ggplot(fdat_amazing) + 
  geom_vline(xintercept = 0, size = 0.75, color = "#737373", alpha=0.5) +
  geom_hline(yintercept = 0, size = 0.75, color = "#737373", alpha=0.5) +
  geom_point(data = fdat_amazing %>% 
               dplyr::filter(Score == "sites"), mapping = aes(x=CCA1, y=CCA2, colour = factor(Score))) + 
  geom_segment(data = fdat_amazing %>% 
                 dplyr::filter(Score == "biplot"), 
               aes(x = 0, xend = CCA1, y = 0, yend = CCA2),
               size = 0.6, 
               alpha=0.8, 
               color = "red",
               arrow = arrow(angle = 3))  + 
  ggforce::geom_mark_ellipse(data = fdat_amazing %>% 
                                      dplyr::filter(Score == "sites"),
                             aes(x=CCA1, y=CCA2, group = Type, label = Type, col = 'grey'),
                  # label.fontsize = 12,
                  label.buffer = unit(2, "mm"),
                  label.minwidth = unit(5, "mm"),
                  con.cap = unit(0.1, "mm"),
                  con.colour='salmon',
                  label.colour='salmon') +
  ggrepel::geom_text_repel(data = fdat_amazing %>% 
                 dplyr::filter(Score == "biplot"), aes(x=CCA1, y=CCA2, label= Label), size=5) +
  xlab(paste0(round(list.pe[1], 3)*100, "% CCA1")) +
  ylab(paste0(round(list.pe[2], 3)*100, "% CCA2")) +
  grids(linetype = "dashed") +
  theme(legend.position = "none", 
        panel.background = element_rect(fill = "white", colour = "grey50")) +
  labs(title = "A. Sites")

 
cca_sampl
```

testing for terms

```
paste("AIC=", extractAIC(rda)[2])
```

```
## [1] "AIC= 412.797063577196"
```

```
vegan::vif.cca(cca)
```

```
##         pH      N.NH4        TOC       P2O5         Cu         Pb         Zn 
##  12.571027  96.189881  99.348838   7.380186  28.849194   4.698127  12.536147 
##         Cd         Ni      N.NO3        TON         CN        K2O 
##  20.124464   7.049732   4.390496 109.600798   2.957706 116.485509
```

```
anova(cca, by='axis')
```

```
## Permutation test for cca under reduced model
## Forward tests for axes
## Permutation: free
## Number of permutations: 999
## 
## Model: cca(formula = otus.ps.vegan ~ pH + N.NH4 + TOC + P2O5 + Cu + Pb + Zn + Cd + Ni + N.NO3 + TOC + TON + CN + K2O, data = metadata)
##          Df ChiSquare       F Pr(>F)    
## CCA1      1   0.89825 29.5483  0.001 ***
## CCA2      1   0.76822 25.2710  0.001 ***
## CCA3      1   0.66278 21.8024  0.001 ***
## CCA4      1   0.56283 18.5144  0.001 ***
## CCA5      1   0.21218  6.9799  0.001 ***
## CCA6      1   0.19000  6.2502  0.001 ***
## CCA7      1   0.18014  5.9257  0.001 ***
## CCA8      1   0.11028  3.6276  0.010 ** 
## CCA9      1   0.08044  2.6462  0.114    
## CCA10     1   0.07102  2.3362  0.148    
## CCA11     1   0.05005  1.6464  0.386    
## CCA12     1   0.03706  1.2191  0.531    
## CCA13     1   0.01857  0.6109  0.871    
## Residual 54   1.64157                   
## ---
## Signif. codes:  0 '***' 0.001 '**' 0.01 '*' 0.05 '.' 0.1 ' ' 1
```

```
anova(cca, by='terms')
```

```
## Permutation test for cca under reduced model
## Terms added sequentially (first to last)
## Permutation: free
## Number of permutations: 999
## 
## Model: cca(formula = otus.ps.vegan ~ pH + N.NH4 + TOC + P2O5 + Cu + Pb + Zn + Cd + Ni + N.NO3 + TOC + TON + CN + K2O, data = metadata)
##          Df ChiSquare       F Pr(>F)    
## pH        1   0.68895 22.6633  0.001 ***
## N.NH4     1   0.62811 20.6619  0.001 ***
## TOC       1   0.27438  9.0258  0.001 ***
## P2O5      1   0.23579  7.7565  0.001 ***
## Cu        1   0.55796 18.3542  0.001 ***
## Pb        1   0.12828  4.2199  0.001 ***
## Zn        1   0.44422 14.6127  0.001 ***
## Cd        1   0.20901  6.8753  0.001 ***
## Ni        1   0.24839  8.1707  0.001 ***
## N.NO3     1   0.11297  3.7161  0.001 ***
## TON       1   0.13064  4.2975  0.001 ***
## CN        1   0.07560  2.4870  0.004 ** 
## K2O       1   0.10753  3.5374  0.001 ***
## Residual 54   1.64157                   
## ---
## Signif. codes:  0 '***' 0.001 '**' 0.01 '*' 0.05 '.' 0.1 ' ' 1
```

testing for margins

```
anova(cca)
```

```
## Permutation test for cca under reduced model
## Permutation: free
## Number of permutations: 999
## 
## Model: cca(formula = otus.ps.vegan ~ pH + N.NH4 + TOC + P2O5 + Cu + Pb + Zn + Cd + Ni + N.NO3 + TOC + TON + CN + K2O, data = metadata)
##          Df ChiSquare      F Pr(>F)    
## Model    13    3.8418 9.7214  0.001 ***
## Residual 54    1.6416                  
## ---
## Signif. codes:  0 '***' 0.001 '**' 0.01 '*' 0.05 '.' 0.1 ' ' 1
```

```
anova(cca, by='margin')
```

```
## Permutation test for cca under reduced model
## Marginal effects of terms
## Permutation: free
## Number of permutations: 999
## 
## Model: cca(formula = otus.ps.vegan ~ pH + N.NH4 + TOC + P2O5 + Cu + Pb + Zn + Cd + Ni + N.NO3 + TOC + TON + CN + K2O, data = metadata)
##          Df ChiSquare       F Pr(>F)    
## pH        1   0.07960  2.6184  0.002 ** 
## N.NH4     1   0.10788  3.5489  0.001 ***
## TOC       1   0.14649  4.8190  0.001 ***
## P2O5      1   0.31866 10.4826  0.001 ***
## Cu        1   0.13748  4.5223  0.001 ***
## Pb        1   0.08387  2.7589  0.001 ***
## Zn        1   0.14999  4.9341  0.001 ***
## Cd        1   0.19379  6.3749  0.001 ***
## Ni        1   0.15948  5.2462  0.001 ***
## N.NO3     1   0.10893  3.5833  0.001 ***
## TON       1   0.15209  5.0031  0.001 ***
## CN        1   0.07419  2.4405  0.005 ** 
## K2O       1   0.10753  3.5374  0.001 ***
## Residual 54   1.64157                   
## ---
## Signif. codes:  0 '***' 0.001 '**' 0.01 '*' 0.05 '.' 0.1 ' ' 1
```

removing redundant factors

```
cca.mod <- vegan::cca(otus.ps.vegan ~  pH + N.NH4 + TOC + P2O5 + Pb + Zn + Cd + Ni + N.NO3 + TOC, data=metadata)


vegan::vif.cca(cca.mod)
```

```
##        pH     N.NH4       TOC      P2O5        Pb        Zn        Cd        Ni 
##  4.419954  4.778005  4.392981  5.957802  3.092375  5.021139 10.080666  2.542477 
##     N.NO3 
##  4.238991
```

```
anova(cca.mod, by='margin')
```

```
## Permutation test for cca under reduced model
## Marginal effects of terms
## Permutation: free
## Number of permutations: 999
## 
## Model: cca(formula = otus.ps.vegan ~ pH + N.NH4 + TOC + P2O5 + Pb + Zn + Cd + Ni + N.NO3 + TOC, data = metadata)
##          Df ChiSquare       F Pr(>F)    
## pH        1   0.19543  5.3591  0.001 ***
## N.NH4     1   0.51016 13.9895  0.001 ***
## TOC       1   0.29517  8.0941  0.001 ***
## P2O5      1   0.33293  9.1297  0.001 ***
## Pb        1   0.05531  1.5167  0.092 .  
## Zn        1   0.21688  5.9474  0.001 ***
## Cd        1   0.27862  7.6402  0.001 ***
## Ni        1   0.25091  6.8803  0.001 ***
## N.NO3     1   0.11620  3.1864  0.002 ** 
## Residual 58   2.11510                   
## ---
## Signif. codes:  0 '***' 0.001 '**' 0.01 '*' 0.05 '.' 0.1 ' ' 1
```

```
anova(cca.mod, by='terms')
```

```
## Permutation test for cca under reduced model
## Terms added sequentially (first to last)
## Permutation: free
## Number of permutations: 999
## 
## Model: cca(formula = otus.ps.vegan ~ pH + N.NH4 + TOC + P2O5 + Pb + Zn + Cd + Ni + N.NO3 + TOC, data = metadata)
##          Df ChiSquare       F Pr(>F)    
## pH        1   0.68895 18.8923  0.001 ***
## N.NH4     1   0.62811 17.2239  0.001 ***
## TOC       1   0.27438  7.5240  0.001 ***
## P2O5      1   0.23579  6.4658  0.001 ***
## Pb        1   0.37660 10.3270  0.001 ***
## Zn        1   0.28832  7.9062  0.001 ***
## Cd        1   0.35144  9.6372  0.001 ***
## Ni        1   0.40850 11.2020  0.001 ***
## N.NO3     1   0.11620  3.1864  0.002 ** 
## Residual 58   2.11510                   
## ---
## Signif. codes:  0 '***' 0.001 '**' 0.01 '*' 0.05 '.' 0.1 ' ' 1
```

```
anova(cca.mod, by='axis')
```

```
## Permutation test for cca under reduced model
## Forward tests for axes
## Permutation: free
## Number of permutations: 999
## 
## Model: cca(formula = otus.ps.vegan ~ pH + N.NH4 + TOC + P2O5 + Pb + Zn + Cd + Ni + N.NO3 + TOC, data = metadata)
##          Df ChiSquare       F Pr(>F)    
## CCA1      1   0.87958 24.1197  0.001 ***
## CCA2      1   0.75964 20.8307  0.001 ***
## CCA3      1   0.66051 18.1124  0.001 ***
## CCA4      1   0.55209 15.1392  0.001 ***
## CCA5      1   0.19117  5.2422  0.001 ***
## CCA6      1   0.15423  4.2292  0.002 ** 
## CCA7      1   0.09216  2.5273  0.020 *  
## CCA8      1   0.05107  1.4005  0.351    
## CCA9      1   0.02785  0.7636  0.716    
## Residual 58   2.11510                   
## ---
## Signif. codes:  0 '***' 0.001 '**' 0.01 '*' 0.05 '.' 0.1 ' ' 1
```

```
anova(cca.mod)
```

```
## Permutation test for cca under reduced model
## Permutation: free
## Number of permutations: 999
## 
## Model: cca(formula = otus.ps.vegan ~ pH + N.NH4 + TOC + P2O5 + Pb + Zn + Cd + Ni + N.NO3 + TOC, data = metadata)
##          Df ChiSquare      F Pr(>F)    
## Model     9    3.3683 10.263  0.001 ***
## Residual 58    2.1151                  
## ---
## Signif. codes:  0 '***' 0.001 '**' 0.01 '*' 0.05 '.' 0.1 ' ' 1
```

```
biplot <- as.data.frame(cca.mod$CCA$biplot)
wa <- as.data.frame(cca.mod$CCA$wa)

biplot <- rownames_to_column(biplot, "Label") %>% 
  add_column(Score = rep("biplot", length(rownames(biplot)))) %>% 
  mutate(
  Group = rep("biplot", length(rownames(biplot))),
  Type = rep("biplot", length(rownames(biplot))),
  Region = rep("biplot", length(rownames(biplot)))
)

wa <- rownames_to_column(wa, "Label") %>% 
  add_column(Score = rep("sites", length(rownames(wa)))) %>% mutate(
  Type = metadata$Type,
  Group = metadata$Group,
  Region = metadata$Region
)

fdat_amazing <- rbind(biplot, wa) 

fdat_amazing <- fdat_amazing %>%
  mutate(label_size = ifelse(Score == "biplot", 4.5, 3)) %>% 
  mutate_if(is.character, as.factor) %>% 
  mutate(Label = case_when(Label == "N.NH4" ~ "N.NH[4]",
                        Label == "P2O5" ~ "P[2]*O[5]",
                        Label == "N.NO3" ~ "N.NO[3]",
                        .default = as.character(Label)))

list.pe <- summary(cca.mod)$cont$importance %>% 
  as.data.frame() %>% 
  select(c("CCA1", "CCA2")) %>% 
  dplyr::slice(2L) %>% 
  t() %>% 
  as.vector()

p.cca_sampl <- ggplot(fdat_amazing) + 
  geom_vline(xintercept = 0, size = 0.75, color = "#737373", alpha=0.5) +
  geom_hline(yintercept = 0, size = 0.75, color = "#737373", alpha=0.5) +
  geom_point(data = fdat_amazing %>% 
               dplyr::filter(Score == "sites"), mapping = aes(x=CCA1, y=CCA2, colour = factor(Score))) + 
  geom_segment(data = fdat_amazing %>% 
                 dplyr::filter(Score == "biplot"), 
               aes(x = 0, xend = CCA1, y = 0, yend = CCA2),
               size = 0.6, 
               alpha=0.8, 
               color = "red",
               arrow = arrow(angle = 3))  + 
  ggforce::geom_mark_ellipse(data = fdat_amazing %>% 
                                      dplyr::filter(Score == "sites"),
                             aes(x=CCA1, y=CCA2, group = Type, label = Type, col = 'grey'),
                  # label.fontsize = 12,
                  label.buffer = unit(2, "mm"),
                  label.minwidth = unit(5, "mm"),
                  con.cap = unit(0.1, "mm"),
                  con.colour='salmon',
                  label.colour='salmon') +
  ggrepel::geom_text_repel(data = fdat_amazing %>% 
                 dplyr::filter(Score == "biplot"), 
                 aes(x=CCA1, y=CCA2, label= Label), 
                 size=5,
                 parse = "TRUE") +
  xlab(paste0(round(list.pe[1], 3)*100, "% CCA1")) +
  ylab(paste0(round(list.pe[2], 3)*100, "% CCA2")) +
  grids(linetype = "dashed") +
  theme(legend.position = "none", 
        panel.background = element_rect(fill = "white", colour = "grey50")) +
  labs(title = "A. Sites")

p.cca_sampl
```

rda, new variant

```
physeq <- ps.anc.f
# physeq <- ps.cr.f
# physeq <- rarefy_even_depth(physeq, rngseed = 1312)

otus.ps.vegan <- veganifyOTU(physeq)
rownames(otus.ps.vegan) <- physeq@sam_data$Type
metadata <- as(sample_data(physeq), "data.frame") 

rda <- vegan::rda(otus.ps.vegan ~ pH + N.NH4 + TOC + P2O5 + Pb + Zn + Cd + Ni + N.NO3 + TOC, data=metadata)

biplot <- as.data.frame(rda$CCA$biplot)
wa <- as.data.frame(rda$CCA$wa)

biplot <- rownames_to_column(biplot, "Label") %>% 
  add_column(Score = rep("biplot", length(rownames(biplot)))) %>% 
  mutate(
  Group = rep("biplot", length(rownames(biplot))),
  Type = rep("biplot", length(rownames(biplot))),
  Region = rep("biplot", length(rownames(biplot)))
)


wa <- rownames_to_column(wa, "Label") %>% 
  add_column(Score = rep("sites", length(rownames(wa)))) %>% mutate(
  Type = metadata$Type,
  Group = metadata$Group,
  Region = metadata$Region
)

fdat_amazing <- rbind(biplot, wa) 

fdat_amazing <- fdat_amazing %>%
  mutate(label_size = ifelse(Score == "biplot", 4.5, 3)) %>% 
  mutate_if(is.character, as.factor)


list.pe <- summary(rda)$cont$importance %>% 
  as.data.frame() %>% 
  select(c("RDA1", "RDA2")) %>% 
  dplyr::slice(2L) %>% 
  t() %>% 
  as.vector()

rda_sampl <- ggplot(fdat_amazing) + 
  geom_vline(xintercept = 0, size = 0.75, color = "#737373", alpha=0.5) +
  geom_hline(yintercept = 0, size = 0.75, color = "#737373", alpha=0.5) +
  geom_point(data = fdat_amazing %>% 
               dplyr::filter(Score == "sites"), mapping = aes(x=RDA1, y=RDA2, colour = factor(Score))) + 
  geom_segment(data = fdat_amazing %>% 
                 dplyr::filter(Score == "biplot"), 
               aes(x = 0, xend = RDA1, y = 0, yend = RDA2),
               size = 0.6, 
               alpha=0.8, 
               color = "red",
               arrow = arrow(angle = 3))  + 
  ggforce::geom_mark_ellipse(data = fdat_amazing %>% 
                                      dplyr::filter(Score == "sites"),
                             aes(x=RDA1, y=RDA2, group = Type, label = Type, col = 'grey'),
                  # label.fontsize = 12,
                  label.buffer = unit(2, "mm"),
                  label.minwidth = unit(5, "mm"),
                  con.cap = unit(0.1, "mm"),
                  con.colour='salmon',
                  label.colour='salmon') +
  ggrepel::geom_text_repel(data = fdat_amazing %>% 
                 dplyr::filter(Score == "biplot"), aes(x=RDA1, y=RDA2, label= Label), size=5) +
  xlab(paste0(round(list.pe[1], 3)*100, "% RDA1")) +
  ylab(paste0(round(list.pe[2], 3)*100, "% RDA2")) +
  grids(linetype = "dashed") +
  theme(legend.position = "none", 
        panel.background = element_rect(fill = "white", colour = "grey50")) +
  labs(title = "A. Sites")

 
rda_sampl
```

```
vegan::vif.cca(rda)
```

```
##        pH     N.NH4       TOC      P2O5        Pb        Zn        Cd        Ni 
##  4.419954  4.778005  4.392981  5.957802  3.092375  5.021139 10.080666  2.542477 
##     N.NO3 
##  4.238991
```

```
anova(rda, by='margin')
```

```
## Permutation test for rda under reduced model
## Marginal effects of terms
## Permutation: free
## Number of permutations: 999
## 
## Model: rda(formula = otus.ps.vegan ~ pH + N.NH4 + TOC + P2O5 + Pb + Zn + Cd + Ni + N.NO3 + TOC, data = metadata)
##          Df Variance       F Pr(>F)    
## pH        1    62.42  9.6205  0.001 ***
## N.NH4     1   124.38 19.1703  0.001 ***
## TOC       1    58.85  9.0707  0.001 ***
## P2O5      1    86.48 13.3288  0.001 ***
## Pb        1    15.44  2.3801  0.024 *  
## Zn        1    54.76  8.4410  0.001 ***
## Cd        1    89.93 13.8611  0.001 ***
## Ni        1   104.58 16.1192  0.001 ***
## N.NO3     1    17.29  2.6642  0.027 *  
## Residual 58   376.30                   
## ---
## Signif. codes:  0 '***' 0.001 '**' 0.01 '*' 0.05 '.' 0.1 ' ' 1
```

```
anova(rda, by='terms')
```

```
## Permutation test for rda under reduced model
## Terms added sequentially (first to last)
## Permutation: free
## Number of permutations: 999
## 
## Model: rda(formula = otus.ps.vegan ~ pH + N.NH4 + TOC + P2O5 + Pb + Zn + Cd + Ni + N.NO3 + TOC, data = metadata)
##          Df Variance       F Pr(>F)    
## pH        1   300.37 46.2966  0.001 ***
## N.NH4     1   329.05 50.7170  0.001 ***
## TOC       1    63.58  9.8004  0.001 ***
## P2O5      1    90.39 13.9316  0.001 ***
## Pb        1   176.04 27.1326  0.001 ***
## Zn        1   110.35 17.0086  0.001 ***
## Cd        1   131.44 20.2585  0.001 ***
## Ni        1   175.02 26.9764  0.001 ***
## N.NO3     1    17.29  2.6642  0.021 *  
## Residual 58   376.30                   
## ---
## Signif. codes:  0 '***' 0.001 '**' 0.01 '*' 0.05 '.' 0.1 ' ' 1
```

```
anova(rda, by='axis')
```

```
## Permutation test for rda under reduced model
## Forward tests for axes
## Permutation: free
## Number of permutations: 999
## 
## Model: rda(formula = otus.ps.vegan ~ pH + N.NH4 + TOC + P2O5 + Pb + Zn + Cd + Ni + N.NO3 + TOC, data = metadata)
##          Df Variance       F Pr(>F)    
## RDA1      1   572.83 88.2917  0.001 ***
## RDA2      1   348.99 53.7907  0.001 ***
## RDA3      1   268.64 41.4052  0.001 ***
## RDA4      1   128.98 19.8794  0.001 ***
## RDA5      1    25.59  3.9449  0.001 ***
## RDA6      1    20.75  3.1981  0.004 ** 
## RDA7      1    13.44  2.0723  0.120    
## RDA8      1     9.21  1.4198  0.324    
## RDA9      1     5.09  0.7838  0.689    
## Residual 58   376.30                   
## ---
## Signif. codes:  0 '***' 0.001 '**' 0.01 '*' 0.05 '.' 0.1 ' ' 1
```

```
anova(rda)
```

```
## Permutation test for rda under reduced model
## Permutation: free
## Number of permutations: 999
## 
## Model: rda(formula = otus.ps.vegan ~ pH + N.NH4 + TOC + P2O5 + Pb + Zn + Cd + Ni + N.NO3 + TOC, data = metadata)
##          Df Variance      F Pr(>F)    
## Model     9   1393.5 23.865  0.001 ***
## Residual 58    376.3                  
## ---
## Signif. codes:  0 '***' 0.001 '**' 0.01 '*' 0.05 '.' 0.1 ' ' 1
```

testing for terms

```
vegan::vif.cca(rda)
```

```
##        pH     N.NH4       TOC      P2O5        Pb        Zn        Cd        Ni 
##  4.419954  4.778005  4.392981  5.957802  3.092375  5.021139 10.080666  2.542477 
##     N.NO3 
##  4.238991
```

```
anova(rda)
```

```
## Permutation test for rda under reduced model
## Permutation: free
## Number of permutations: 999
## 
## Model: rda(formula = otus.ps.vegan ~ pH + N.NH4 + TOC + P2O5 + Pb + Zn + Cd + Ni + N.NO3 + TOC, data = metadata)
##          Df Variance      F Pr(>F)    
## Model     9   1393.5 23.865  0.001 ***
## Residual 58    376.3                  
## ---
## Signif. codes:  0 '***' 0.001 '**' 0.01 '*' 0.05 '.' 0.1 ' ' 1
```

```
anova(rda, by='terms')
```

```
## Permutation test for rda under reduced model
## Terms added sequentially (first to last)
## Permutation: free
## Number of permutations: 999
## 
## Model: rda(formula = otus.ps.vegan ~ pH + N.NH4 + TOC + P2O5 + Pb + Zn + Cd + Ni + N.NO3 + TOC, data = metadata)
##          Df Variance       F Pr(>F)    
## pH        1   300.37 46.2966  0.001 ***
## N.NH4     1   329.05 50.7170  0.001 ***
## TOC       1    63.58  9.8004  0.001 ***
## P2O5      1    90.39 13.9316  0.001 ***
## Pb        1   176.04 27.1326  0.001 ***
## Zn        1   110.35 17.0086  0.001 ***
## Cd        1   131.44 20.2585  0.001 ***
## Ni        1   175.02 26.9764  0.001 ***
## N.NO3     1    17.29  2.6642  0.020 *  
## Residual 58   376.30                   
## ---
## Signif. codes:  0 '***' 0.001 '**' 0.01 '*' 0.05 '.' 0.1 ' ' 1
```

testing for margins

```
anova(rda, by='margin')
```

```
## Permutation test for rda under reduced model
## Marginal effects of terms
## Permutation: free
## Number of permutations: 999
## 
## Model: rda(formula = otus.ps.vegan ~ pH + N.NH4 + TOC + P2O5 + Pb + Zn + Cd + Ni + N.NO3 + TOC, data = metadata)
##          Df Variance       F Pr(>F)    
## pH        1    62.42  9.6205  0.001 ***
## N.NH4     1   124.38 19.1703  0.001 ***
## TOC       1    58.85  9.0707  0.001 ***
## P2O5      1    86.48 13.3288  0.001 ***
## Pb        1    15.44  2.3801  0.024 *  
## Zn        1    54.76  8.4410  0.001 ***
## Cd        1    89.93 13.8611  0.001 ***
## Ni        1   104.58 16.1192  0.001 ***
## N.NO3     1    17.29  2.6642  0.015 *  
## Residual 58   376.30                   
## ---
## Signif. codes:  0 '***' 0.001 '**' 0.01 '*' 0.05 '.' 0.1 ' ' 1
```

```
paste("AIC full model =", extractAIC(cca)[2] %>% round(2))
```

```
## [1] "AIC full model = 603.79"
```

```
paste("AIC mod =", extractAIC(cca.mod)[2] %>%  round(2))
```

```
## [1] "AIC mod = 613.02"
```

```
ggarrange(rda_sampl, p.cca_sampl)
```

Combining with the WGCNA data

```
physeq <-  rarefy_even_depth(ps.cr.f, rngseed = 1312)

veganifyOTU <- function(physeq){
  require(phyloseq)
  if(taxa_are_rows(physeq)){physeq <- t(physeq)}
  return(as(otu_table(physeq), "matrix"))
}

otus.ps.vegan <- veganifyOTU(physeq)
rownames(otus.ps.vegan) <- physeq@sam_data$Group
metadata <- as(sample_data(physeq), "data.frame")

cca_w_varstab_asv <- vegan::cca(otus.ps.vegan ~ pH + N.NH4 + TOC + P2O5 + Pb + Zn + Cd + Ni + N.NO3 + TOC, data=metadata)

tx.ids  <- tx %>% 
  rownames_to_column("ID") %>% 
  mutate(Score = "sites")

cca.meta <-  cca_w_varstab_asv$CCA$biplot %>% 
  as.data.frame() %>% 
  select(c("CCA1", "CCA2")) %>% 
  rownames_to_column("ID") %>% 
  mutate(Score = "biplot")

list.pe <-  summary(cca_w_varstab_asv)$cont$importance %>% 
  as.data.frame() %>% 
  select(c("CCA1", "CCA2")) %>% 
  dplyr::slice(2L) %>% 
  t() %>% 
  as.vector()

fdat_amazing <- as.data.frame(cca_w_varstab_asv$CCA$v) %>%
  select(c("CCA1", "CCA2")) %>% 
  rownames_to_column("ID") %>% 
  right_join(tx.ids, by = join_by(ID)) %>% 
  mutate(label_size = 0) %>% 
  bind_rows(cca.meta) %>% 
  mutate(Group = as.factor(big_clusters)) %>% 
  filter(Group != 'NON' | Score == "biplot")  %>% 
  mutate(ID = case_when(ID == "N.NH4" ~ "N.NH[4]",
                        ID == "P2O5" ~ "P[2]*O[5]",
                        ID == "N.NO3" ~ "N.NO[3]",
                        .default = as.character(ID)))

cca_clust <- ggplot(fdat_amazing) + 
  geom_point(data = fdat_amazing %>% dplyr::filter(Score == "sites"), 
             mapping = aes(x=CCA1, 
                           y=CCA2, 
                           color=Group, 
                           size=abnd), 
             alpha=0.5) + 
  geom_segment(data = fdat_amazing %>% dplyr::filter(Score == "biplot"), 
               aes(x = 0, 
                   xend = CCA1, 
                   y = 0, 
                   yend = CCA2), 
               alpha=0.8, 
               color = "red", 
               arrow = arrow(angle = 3)) +
  geom_text_repel(data = fdat_amazing %>% dplyr::filter(Score == "biplot"),
                  aes(x=CCA1, 
                      y=CCA2, 
                      label= ID), 
                  size=5,
                  parse = TRUE) + 
  xlab(paste0(round(list.pe[1], 3)*100, "% CCA1")) +
  ylab(paste0(round(list.pe[2], 3)*100, "% CCA2")) +
  grids(linetype = "dashed") +
  geom_vline(xintercept = 0, size = 0.75, color = "#737373", alpha=0.5) +
  geom_hline(yintercept = 0, size = 0.75, color = "#737373", alpha=0.5) +
  theme(legend.position = "none", 
        panel.background = element_rect(fill = "white", colour = "grey50")) +
  scale_colour_viridis_d(option = "magma", 
                       aesthetics = "color", 
                       begin = 0.2, 
                       end = 0.8)  +
  labs(title = "B. ASVs - WGCNA clusters")

cca_clust
```

```
gridExtra::grid.arrange(p.cca_sampl, 
    cca_clust, 
    ncol = 2, 
    nrow=1,
    widths = c(1, 1))
```

Oh, it’s much worse than before.

```
gridExtra::grid.arrange(p_a1, p_a3, beta_all,
             ncol = 2, nrow = 2, 
             heights = c(1, 1), 
             layout_matrix= rbind(c(1, 2),c(3, 3)))
```

```
sessionInfo()
```

```
## R version 4.3.2 (2023-10-31)
## Platform: x86_64-pc-linux-gnu (64-bit)
## Running under: Ubuntu 20.04.6 LTS
## 
## Matrix products: default
## BLAS:   /usr/lib/x86_64-linux-gnu/blas/libblas.so.3.9.0 
## LAPACK: /usr/lib/x86_64-linux-gnu/lapack/liblapack.so.3.9.0
## 
## locale:
##  [1] LC_CTYPE=en_US.UTF-8       LC_NUMERIC=C              
##  [3] LC_TIME=en_US.UTF-8        LC_COLLATE=en_US.UTF-8    
##  [5] LC_MONETARY=en_US.UTF-8    LC_MESSAGES=en_US.UTF-8   
##  [7] LC_PAPER=en_US.UTF-8       LC_NAME=C                 
##  [9] LC_ADDRESS=C               LC_TELEPHONE=C            
## [11] LC_MEASUREMENT=en_US.UTF-8 LC_IDENTIFICATION=C       
## 
## time zone: Europe/Moscow
## tzcode source: system (glibc)
## 
## attached base packages:
## [1] stats     graphics  grDevices utils     datasets  methods   base     
## 
## other attached packages:
##  [1] ggforce_0.4.2         ggrepel_0.9.5         KneeArrower_1.0.0    
##  [4] ggtree_3.8.2          heatmaply_1.5.0       viridis_0.6.5        
##  [7] viridisLite_0.4.2     plotly_4.10.4         ampvis2_2.7.28       
## [10] ggpubr_0.6.0          lubridate_1.9.3       forcats_1.0.0        
## [13] stringr_1.5.1         dplyr_1.1.4           purrr_1.0.2          
## [16] readr_2.1.5           tidyr_1.3.1           tibble_3.2.1         
## [19] ggplot2_3.5.0         tidyverse_2.0.0       phyloseq_1.44.0      
## [22] WGCNA_1.72-5          fastcluster_1.2.6     dynamicTreeCut_1.63-1
## 
## loaded via a namespace (and not attached):
##   [1] IRanges_2.34.1                 gld_2.6.6                     
##   [3] nnet_7.3-19                    ggfittext_0.10.2              
##   [5] DT_0.32                        Biostrings_2.68.1             
##   [7] TH.data_1.1-2                  vctrs_0.6.5                   
##   [9] energy_1.7-11                  digest_0.6.35                 
##  [11] png_0.1-8                      proxy_0.4-27                  
##  [13] Exact_3.2                      registry_0.5-1                
##  [15] permute_0.9-7                  MASS_7.3-60.0.1               
##  [17] reshape2_1.4.4                 foreach_1.5.2                 
##  [19] BiocGenerics_0.46.0            withr_3.0.0                   
##  [21] xfun_0.42                      ggfun_0.1.4                   
##  [23] ellipsis_0.3.2                 survival_3.5-8                
##  [25] doRNG_1.8.6                    memoise_2.0.1                 
##  [27] ggbeeswarm_0.7.2               gmp_0.7-4                     
##  [29] systemfonts_1.0.6              tidytree_0.4.6                
##  [31] zoo_1.8-12                     gtools_3.9.5                  
##  [33] DEoptimR_1.1-3                 Formula_1.2-5                 
##  [35] KEGGREST_1.40.1                httr_1.4.7                    
##  [37] rstatix_0.7.2                  rhdf5filters_1.12.1           
##  [39] rhdf5_2.44.0                   rstudioapi_0.15.0             
##  [41] ggVennDiagram_1.5.2            generics_0.1.3                
##  [43] base64enc_0.1-3                S4Vectors_0.38.2              
##  [45] zlibbioc_1.46.0                ScaledMatrix_1.8.1            
##  [47] polyclip_1.10-6                ca_0.71.1                     
##  [49] GenomeInfoDbData_1.2.10        ade4_1.7-22                   
##  [51] doParallel_1.0.17              evaluate_0.23                 
##  [53] S4Arrays_1.0.6                 preprocessCore_1.62.1         
##  [55] hms_1.1.3                      GenomicRanges_1.52.1          
##  [57] irlba_2.3.5.1                  colorspace_2.1-0              
##  [59] readxl_1.4.3                   magrittr_2.0.3                
##  [61] lattice_0.22-5                 robustbase_0.99-2             
##  [63] DECIPHER_2.28.0                scuttle_1.10.3                
##  [65] cowplot_1.1.3                  matrixStats_1.2.0             
##  [67] class_7.3-22                   Hmisc_5.1-2                   
##  [69] pillar_1.9.0                   nlme_3.1-164                  
##  [71] iterators_1.0.14               decontam_1.20.0               
##  [73] compiler_4.3.2                 beachmat_2.16.0               
##  [75] stringi_1.8.3                  biomformat_1.28.0             
##  [77] treemapify_2.5.6               DescTools_0.99.54             
##  [79] TSP_1.2-4                      minqa_1.2.6                   
##  [81] SummarizedExperiment_1.30.2    dendextend_1.17.1             
##  [83] plyr_1.8.9                     crayon_1.5.2                  
##  [85] abind_1.4-5                    scater_1.28.0                 
##  [87] gridGraphics_0.5-1             bit_4.0.5                     
##  [89] mia_1.8.0                      rootSolve_1.8.2.4             
##  [91] sandwich_3.1-0                 codetools_0.2-19              
##  [93] multcomp_1.4-25                BiocSingular_1.16.0           
##  [95] crosstalk_1.2.1                bslib_0.6.1                   
##  [97] e1071_1.7-14                   lmom_3.0                      
##  [99] multtest_2.56.0                MultiAssayExperiment_1.26.0   
## [101] splines_4.3.2                  Rcpp_1.0.12                   
## [103] sparseMatrixStats_1.12.2       cellranger_1.1.0              
## [105] knitr_1.45                     egg_0.4.5                     
## [107] blob_1.2.4                     utf8_1.2.4                    
## [109] lme4_1.1-35.1                  fs_1.6.3                      
## [111] checkmate_2.3.1                DelayedMatrixStats_1.22.6     
## [113] Rdpack_2.6                     expm_0.999-9                  
## [115] gsl_2.1-8                      ggsignif_0.6.4                
## [117] ggplotify_0.1.2                Matrix_1.6-5                  
## [119] tzdb_0.4.0                     tweenr_2.0.3                  
## [121] pkgconfig_2.0.3                tools_4.3.2                   
## [123] cachem_1.0.8                   rbibutils_2.2.16              
## [125] RSQLite_2.3.5                  DBI_1.2.2                     
## [127] numDeriv_2016.8-1.1            signal_1.8-0                  
## [129] impute_1.74.1                  fastmap_1.1.1                 
## [131] rmarkdown_2.26                 scales_1.3.0                  
## [133] grid_4.3.2                     broom_1.0.5                   
## [135] sass_0.4.8                     patchwork_1.2.0               
## [137] carData_3.0-5                  rpart_4.1.23                  
## [139] farver_2.1.1                   mgcv_1.9-1                    
## [141] yaml_2.3.8                     MatrixGenerics_1.12.3         
## [143] foreign_0.8-86                 bayesm_3.1-6                  
## [145] cli_3.6.2                      stats4_4.3.2                  
## [147] webshot_0.5.5                  lifecycle_1.0.4               
## [149] Biobase_2.60.0                 mvtnorm_1.2-4                 
## [151] backports_1.4.1                BiocParallel_1.34.2           
## [153] timechange_0.3.0               gtable_0.3.4                  
## [155] ANCOMBC_2.2.2                  parallel_4.3.2                
## [157] ape_5.7-1                      CVXR_1.0-12                   
## [159] jsonlite_1.8.8                 seriation_1.5.4               
## [161] bitops_1.0-7                   bit64_4.0.5                   
## [163] assertthat_0.2.1               yulab.utils_0.1.4             
## [165] vegan_2.6-4                    BiocNeighbors_1.18.0          
## [167] TreeSummarizedExperiment_2.8.0 jquerylib_0.1.4               
## [169] highr_0.10                     lazyeval_0.2.2                
## [171] htmltools_0.5.7                GO.db_3.17.0                  
## [173] glue_1.7.0                     XVector_0.40.0                
## [175] RCurl_1.98-1.14                treeio_1.24.3                 
## [177] gridExtra_2.3                  boot_1.3-30                   
## [179] igraph_2.0.2                   R6_2.5.1                      
## [181] SingleCellExperiment_1.22.0    Rmpfr_0.9-5                   
## [183] labeling_0.4.3                 cluster_2.1.6                 
## [185] rngtools_1.5.2                 Rhdf5lib_1.22.1               
## [187] aplot_0.2.2                    GenomeInfoDb_1.36.4           
## [189] nloptr_2.0.3                   compositions_2.0-8            
## [191] DirichletMultinomial_1.42.0    DelayedArray_0.26.7           
## [193] tidyselect_1.2.1               vipor_0.4.7                   
## [195] htmlTable_2.4.2                tensorA_0.36.2.1              
## [197] car_3.1-2                      AnnotationDbi_1.62.2          
## [199] rsvd_1.0.5                     munsell_0.5.0                 
## [201] data.table_1.15.2              htmlwidgets_1.6.4             
## [203] RColorBrewer_1.1-3             rlang_1.1.3                   
## [205] lmerTest_3.1-3                 fansi_1.0.6                   
## [207] beeswarm_0.4.0
```
