# Supplementary material for: Linking the composition of cryoconite prokaryotic communities in the Arctic, Antarctic, and Central Caucasus with their chemical characteristics
Source: Sci Rep. 2024 Jul 9;14:15838. doi: 10.1038/s41598-024-64452-3 (PMC11233692; doi:10.1038/s41598-024-64452-3)
Supplement: Supplementary file 2 — Supplementary Information 2. [file 41598_2024_64452_MOESM2_ESM.pptx]

## Slide 1
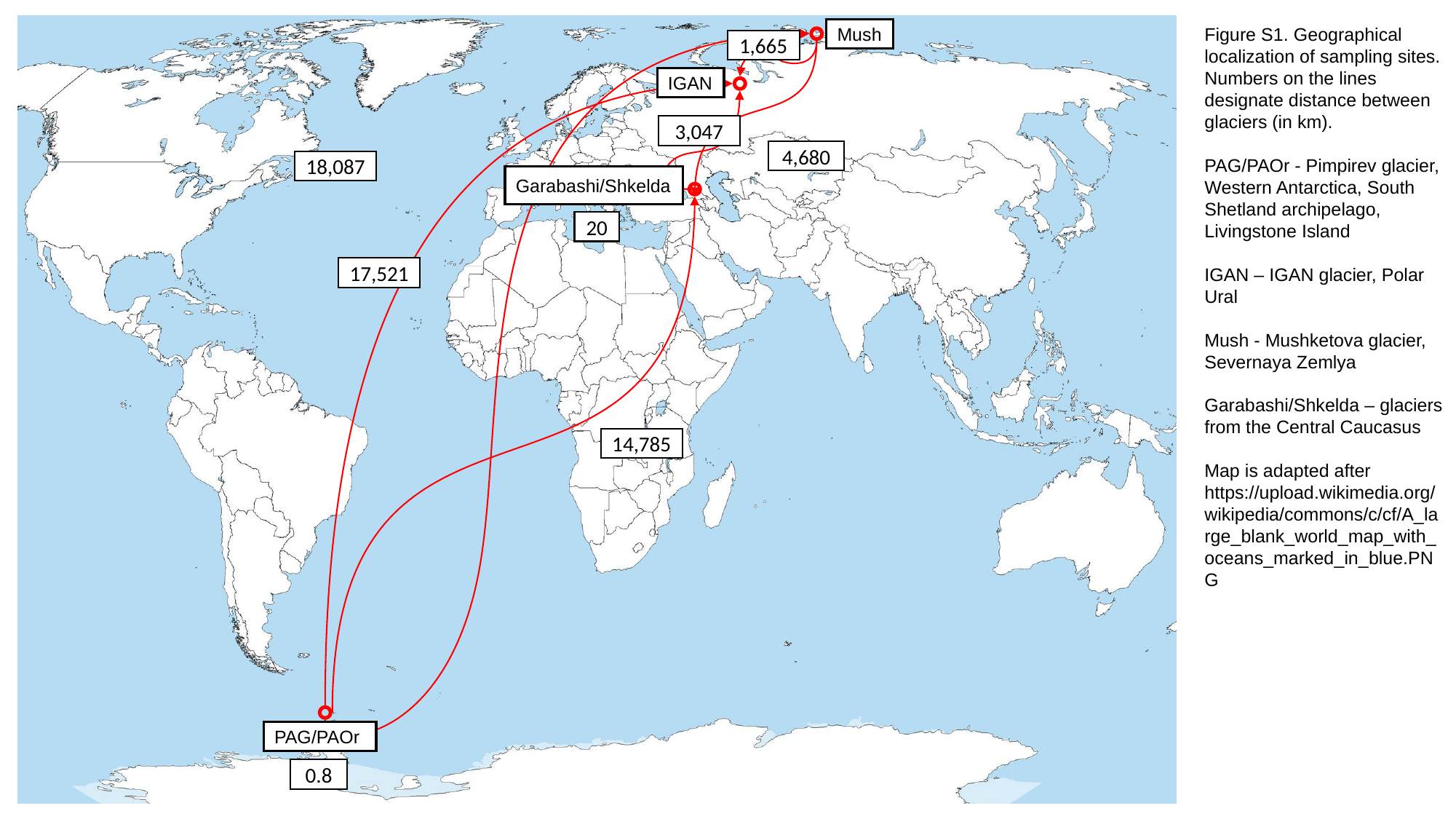

Figure S1. Geographical localization of sampling sites. Numbers on the lines designate distance between glaciers (in km).
PAG/PAOr - Pimpirev glacier, Western Antarctica, South Shetland archipelago, Livingstone Island
IGAN – IGAN glacier, Polar Ural
Mush - Mushketova glacier, Severnaya Zemlya
Garabashi/Shkelda – glaciers from the Central Caucasus
Map is adapted after https://upload.wikimedia.org/wikipedia/commons/c/cf/A_large_blank_world_map_with_oceans_marked_in_blue.PNG
Mush
1,665
IGAN
3,047
4,680
18,087
Garabashi/Shkelda
20
17,521
14,785
PAG/PAOr
0.8

## Slide 2
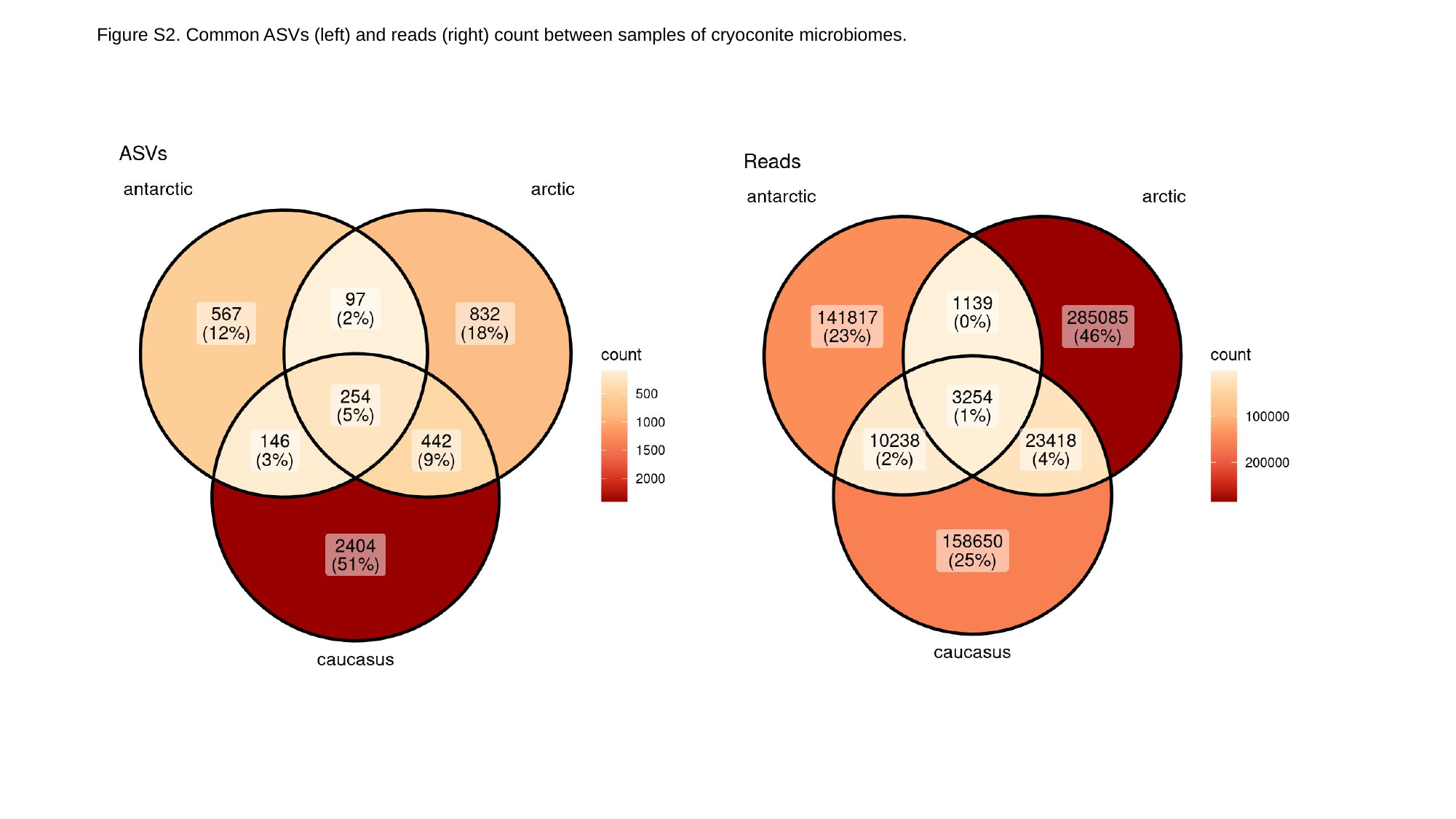

Figure S2. Common ASVs (left) and reads (right) count between samples of cryoconite microbiomes.

## Slide 3
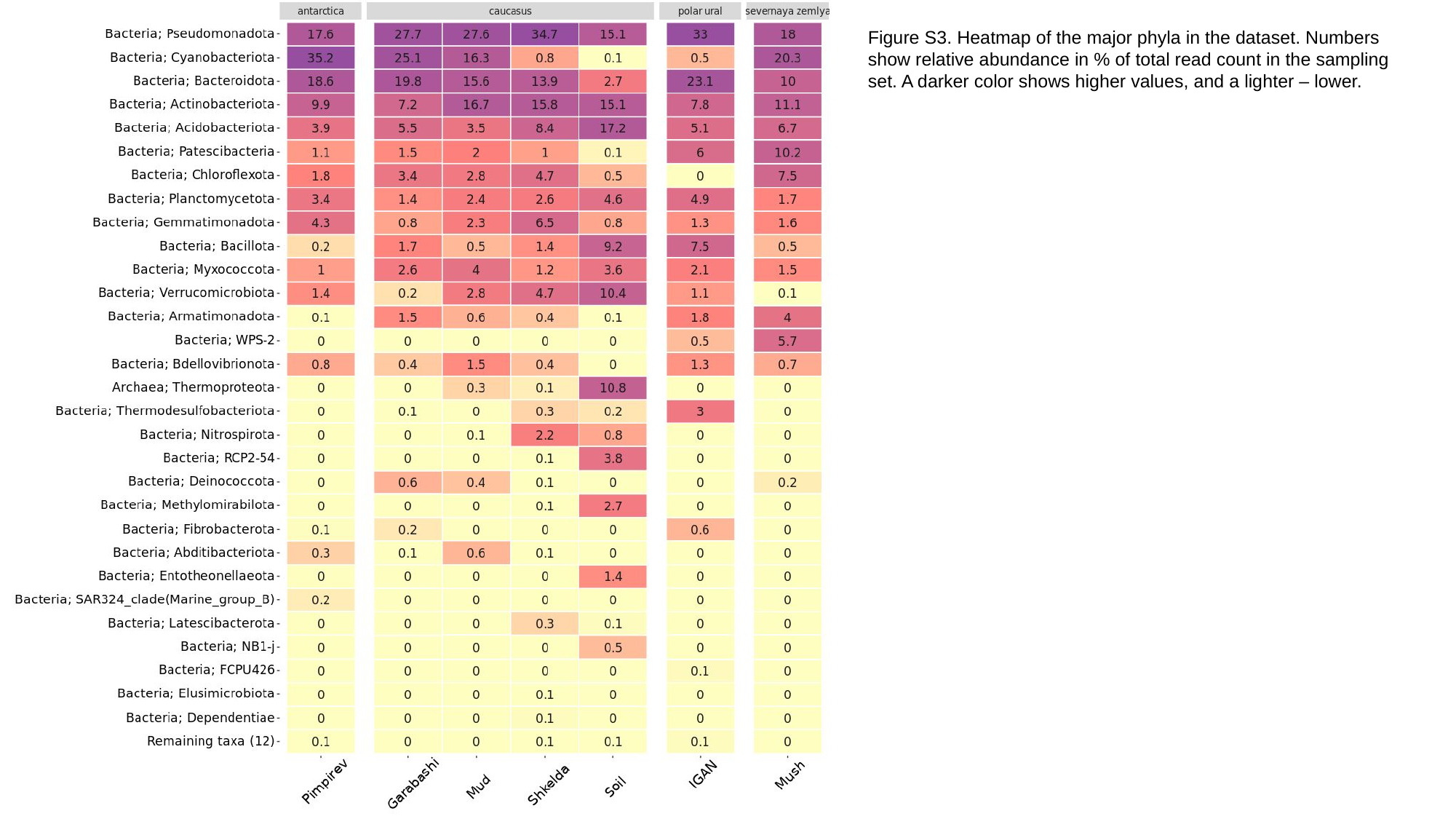

Figure S3. Heatmap of the major phyla in the dataset. Numbers show relative abundance in % of total read count in the sampling set. A darker color shows higher values, and a lighter – lower.

## Slide 4
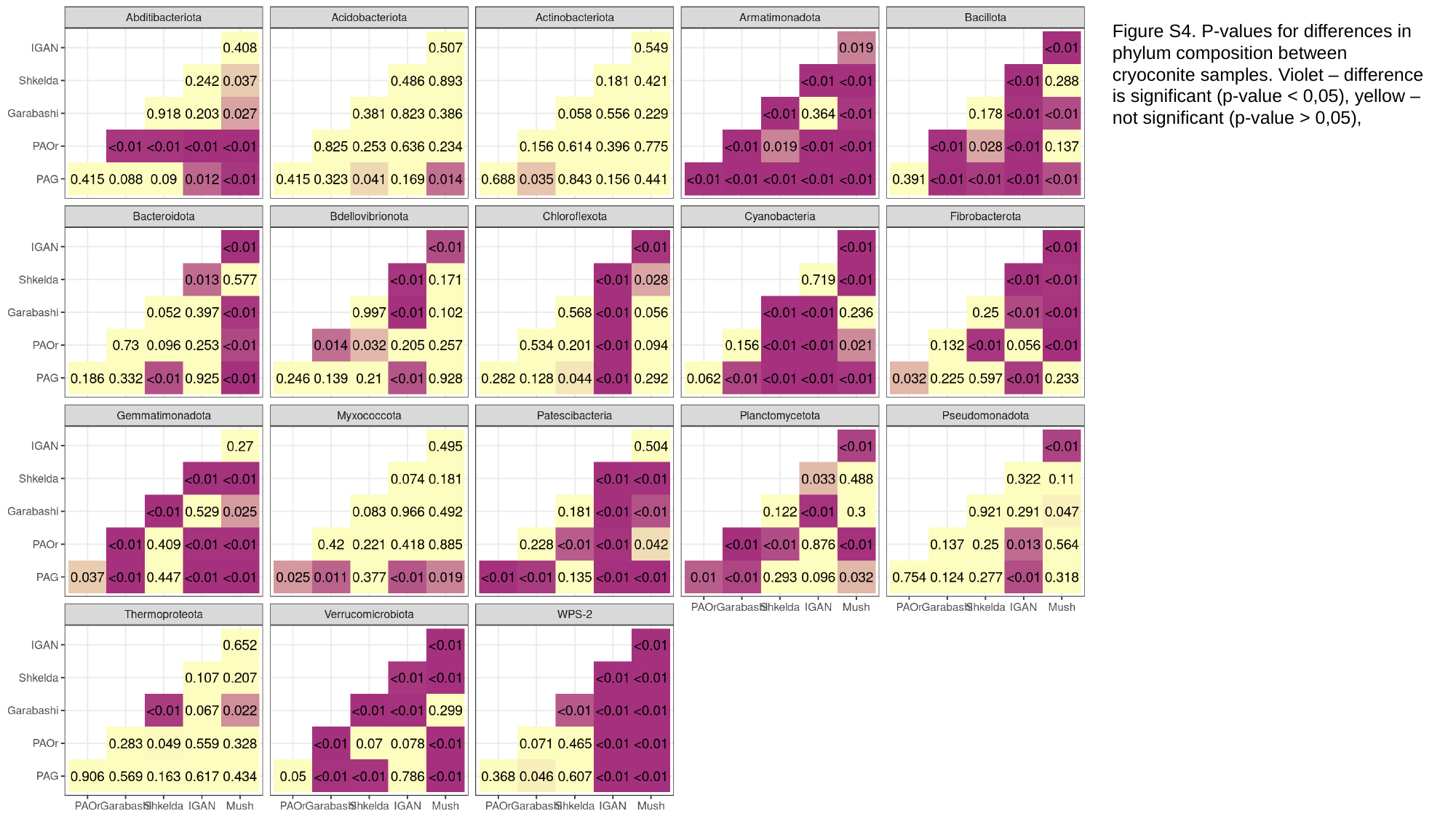

Figure S4. P-values for differences in phylum composition between cryoconite samples. Violet – difference is significant (p-value < 0,05), yellow – not significant (p-value > 0,05),

## Slide 5
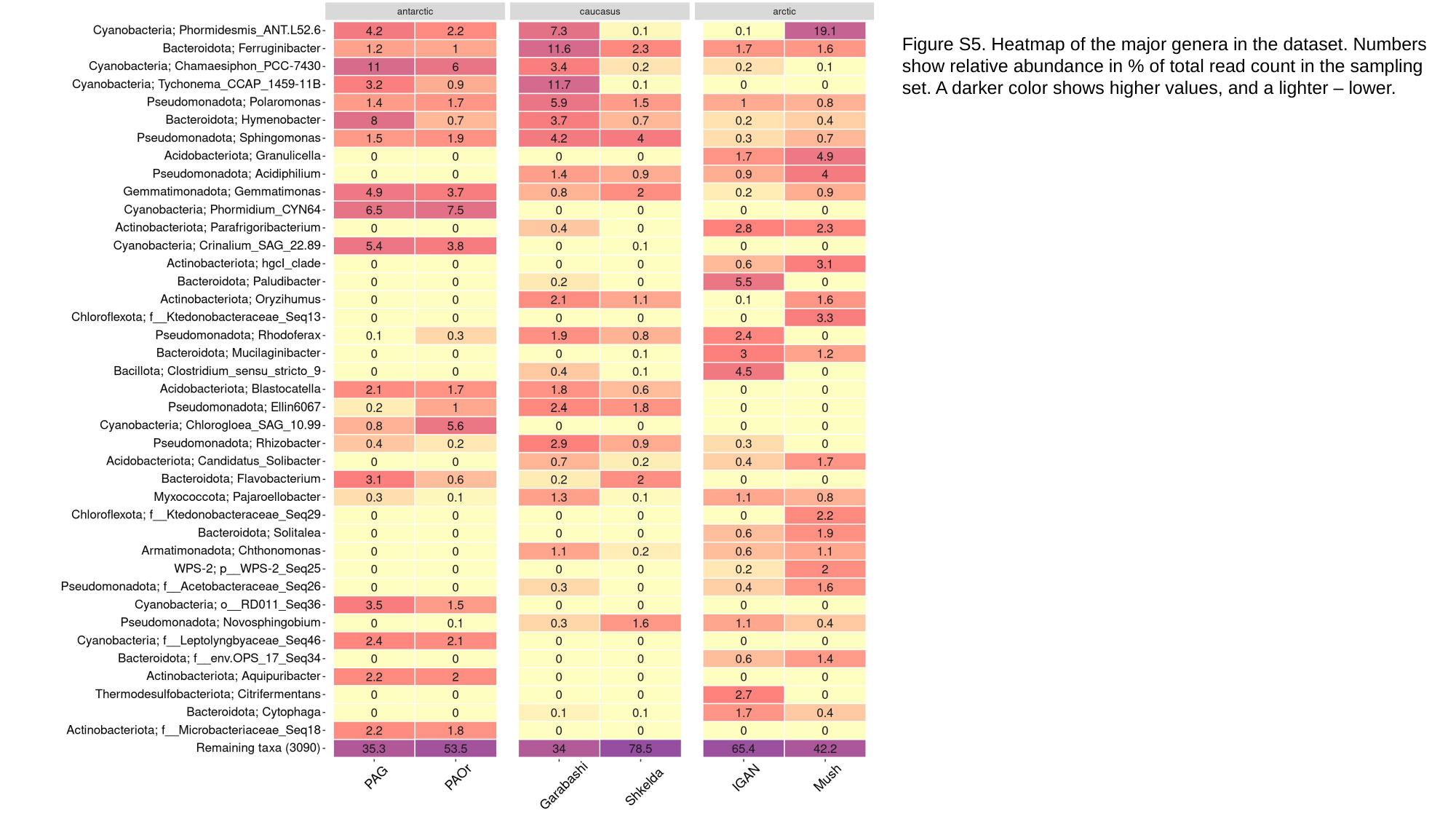

Figure S5. Heatmap of the major genera in the dataset. Numbers show relative abundance in % of total read count in the sampling set. A darker color shows higher values, and a lighter – lower.

## Slide 6
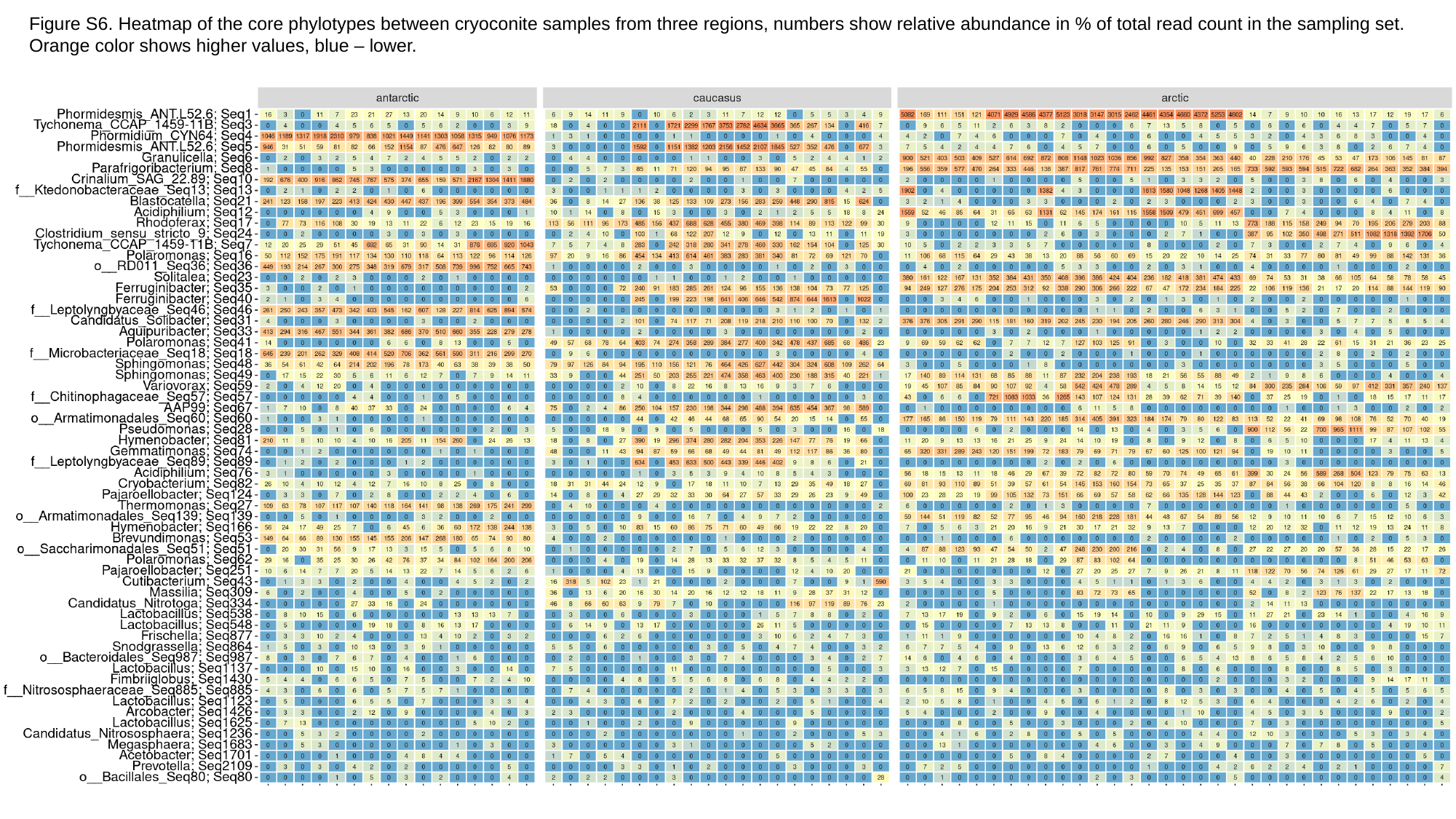

Figure S6. Heatmap of the core phylotypes between cryoconite samples from three regions, numbers show relative abundance in % of total read count in the sampling set. Orange color shows higher values, blue – lower.

## Slide 7
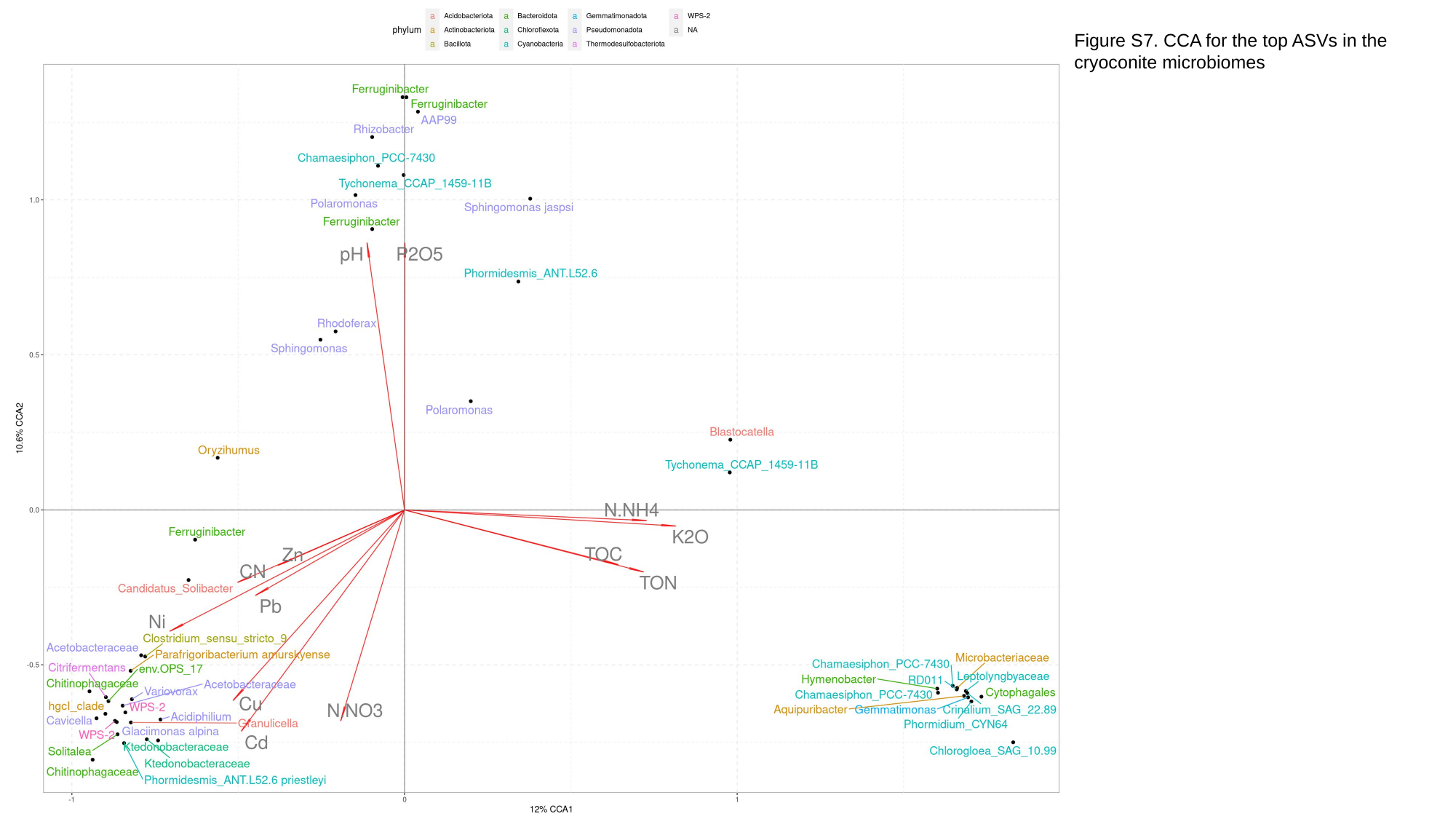

Figure S7. CCA for the top ASVs in the cryoconite microbiomes

## Slide 8
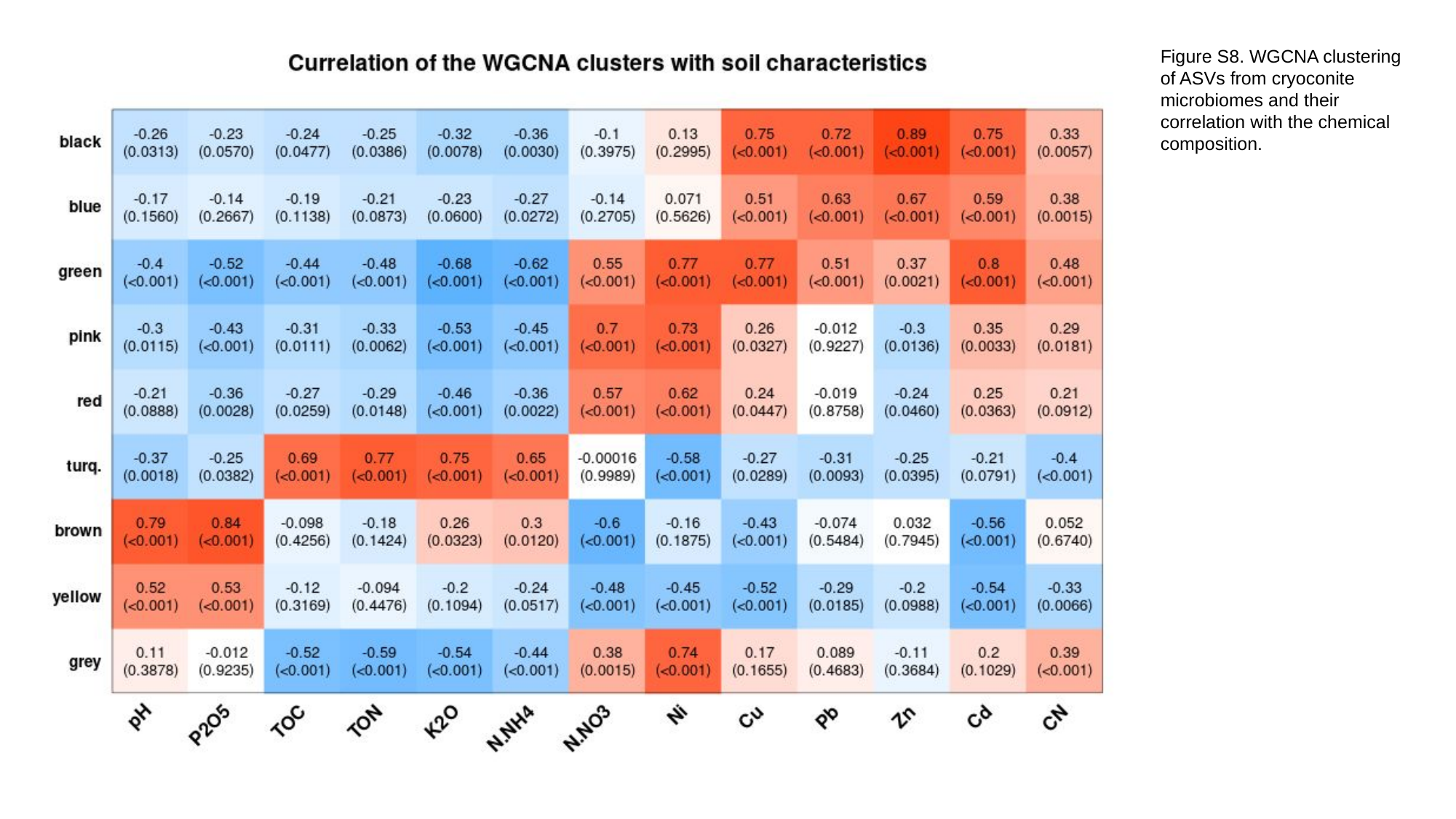

Figure S8. WGCNA clustering of ASVs from cryoconite microbiomes and their correlation with the chemical composition.

## Slide 9
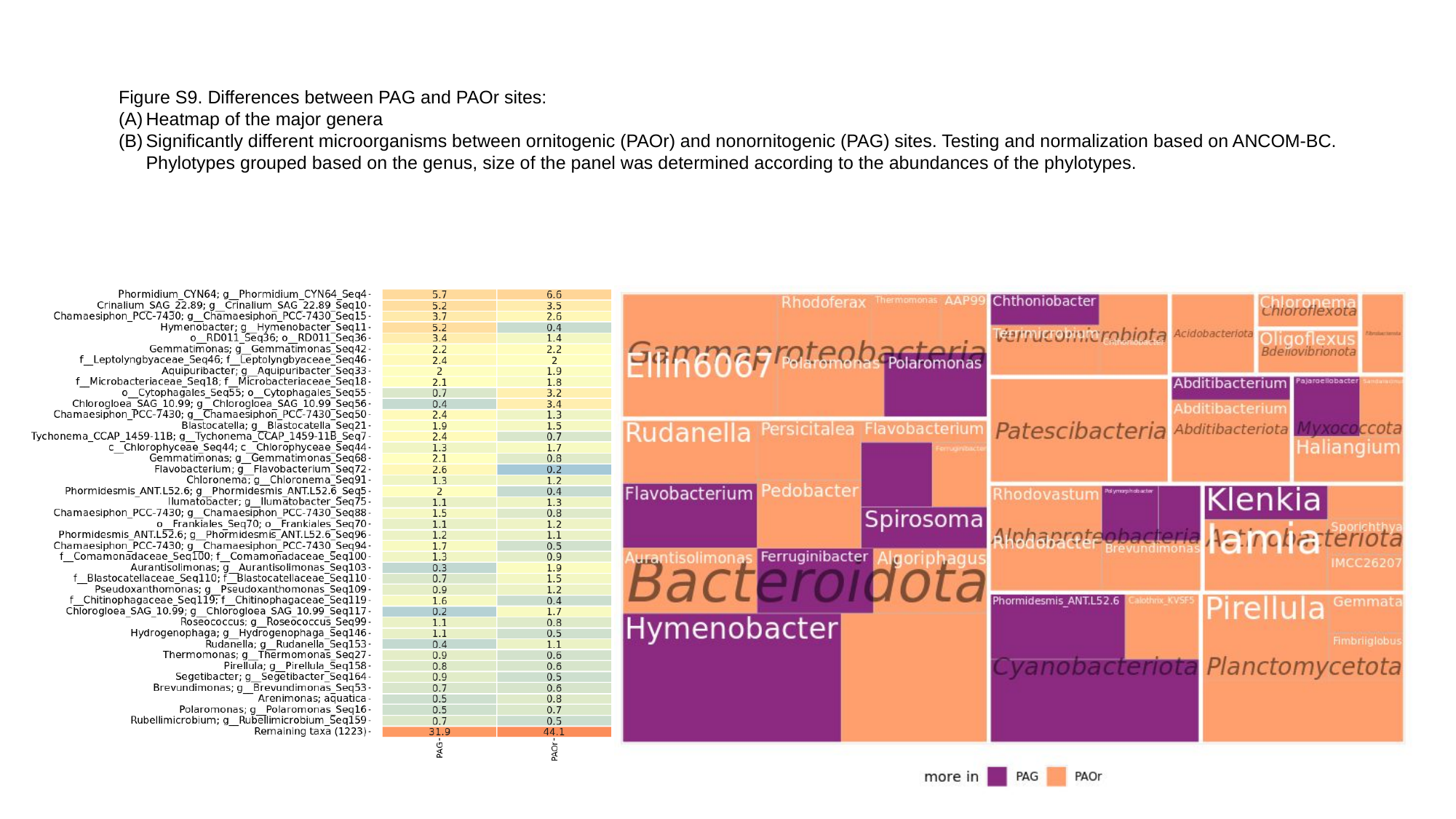

Figure S9. Differences between PAG and PAOr sites:
Heatmap of the major genera
Significantly different microorganisms between ornitogenic (PAOr) and nonornitogenic (PAG) sites. Testing and normalization based on ANCOM-BC. Phylotypes grouped based on the genus, size of the panel was determined according to the abundances of the phylotypes.

## Slide 10
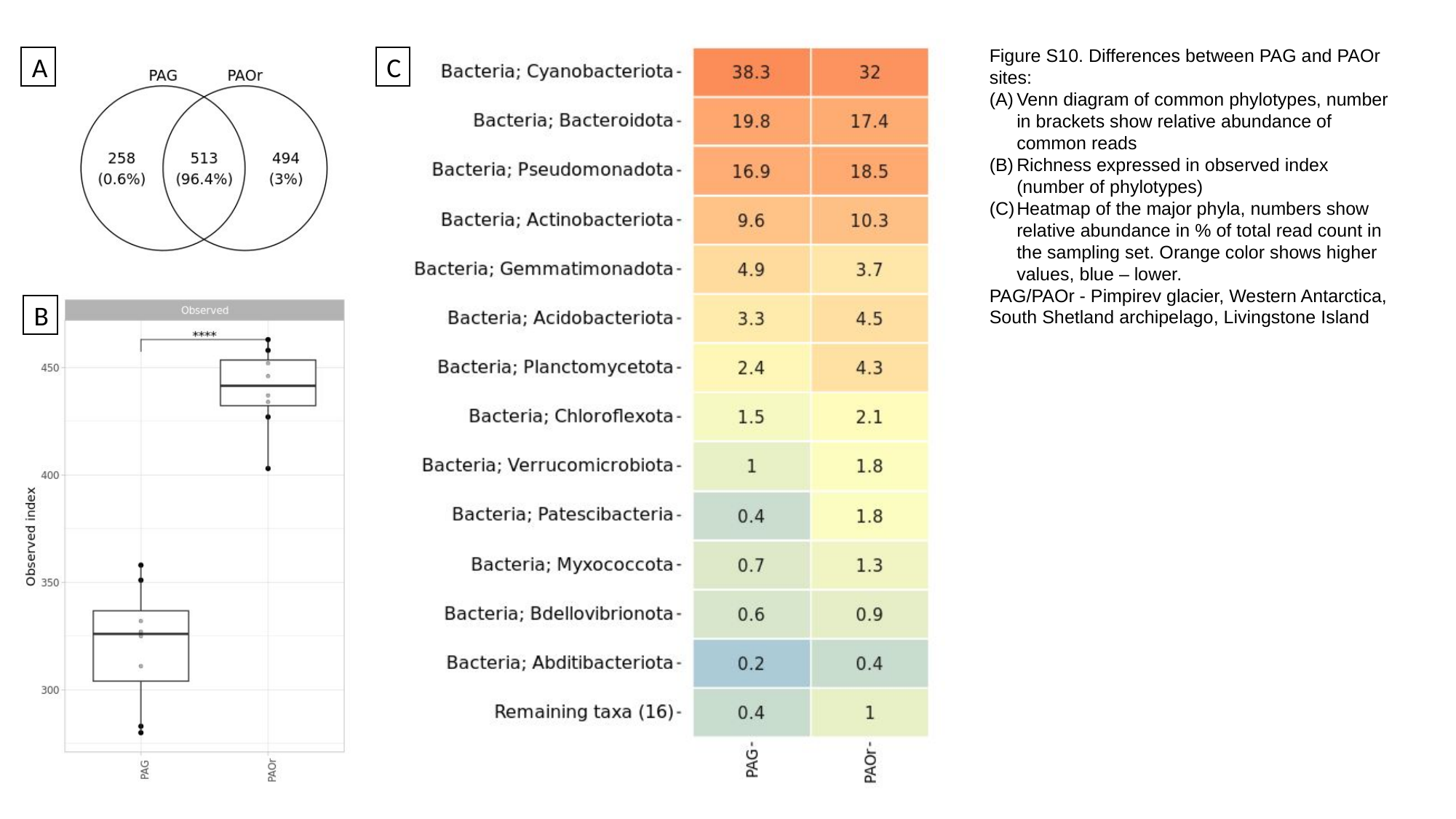

Figure S10. Differences between PAG and PAOr sites:
Venn diagram of common phylotypes, number in brackets show relative abundance of common reads
Richness expressed in observed index (number of phylotypes)
Heatmap of the major phyla, numbers show relative abundance in % of total read count in the sampling set. Orange color shows higher values, blue – lower.
PAG/PAOr - Pimpirev glacier, Western Antarctica, South Shetland archipelago, Livingstone Island
A
C
B
